# Supplementary material for: Stage Transitions in Lucilia sericata and Phormia regina (Diptera: Calliphoridae) and Implications for Forensic Science
Source: Insects. 2023 Mar 25;14(4):315. doi: 10.3390/insects14040315 (PMC10145051; doi:10.3390/insects14040315)
Supplement: Supplementary file 1 [file insects-14-00315-s001.zip › insects-2291479-supplementary.pdf]

Supplementary Materials S1

Development data for *Lucilia sericata* (from Excel).

Transitions at 50% by Dose Analysis							
Temp		Hours to Stage Transition					
Mean	SE	E-L1	E-L2	E-L3f	E-L3m	E-P	E-A
10.4	0.51	155.0	317.3	451.5	683.8	1380.2	3798.4
12.7	0.18	32.3	136.1	230.5	431.3	1970.6	2891.0
15.1	0.14	51.7	137.6	179.1	313.8	754.0	1708.5
17.5	0.12	45.6	85.4	136.6	237.2	326.4	760.8
20.1	0.09	28.0	57.9	92.1	189.3	217.0	553.4
22.5	0.10	22.1	45.7	77.5	141.1	202.4	424.6
25.0	0.06	16.9	38.4	62.3	111.3	167.8	370.2
27.5	0.06	11.8	30.9	46.5	124.3	157.6	344.5
30.0	0.05	11.4	24.6	41.6	86.7	139.3	297.2
32.5	0.02	9.7	19.2	35.6	84.5	156.8	308.4

Temp		Hours in Stage					
Mean	SE	Egg	L1	L2	L3f	L3m	P
10.4	0.51	155.0	162.3	134.2	232.3	696.3	2418.3
12.7	0.18	32.3	103.7	94.4	200.8	1539.3	920.4
15.1	0.14	51.7	85.9	41.5	134.7	440.2	954.5
17.5	0.12	45.6	39.8	51.2	100.6	89.2	434.4
20.1	0.09	28.0	29.9	34.2	97.2	27.7	336.4
22.5	0.10	22.1	23.6	31.8	63.6	61.3	222.2
25.0	0.06	16.9	21.5	23.9	49.0	56.4	202.4
27.5	0.06	11.8	19.0	15.6	77.8	33.3	186.9
30.0	0.05	11.4	13.2	17.0	45.1	52.6	157.9
32.5	0.02	9.7	9.5	16.4	48.9	72.3	151.6

Temp		Days to Stage Transition					
Mean	SE	E-L1	E-L2	E-L3f	E-L3m	E-P	E-A
10.4	0.16	6.5	13.2	18.8	28.5	57.5	158.3
12.7	0.13	1.3	5.7	9.6	18.0	82.1	120.5
15.1	0.15	2.2	5.7	7.5	13.1	31.4	71.2
17.5	0.09	1.9	3.6	5.7	9.9	13.6	31.7
20.1	0.1	1.2	2.4	3.8	7.9	9.0	23.1
22.5	0.90	0.9	1.9	3.2	5.9	8.4	17.7
25.0	0.08	0.7	1.6	2.6	4.6	7.0	15.4
27.5	0.07	0.5	1.3	1.9	5.2	6.6	14.4
30.0	0.23	0.5	1.0	1.7	3.6	5.8	12.4
32.5	0.07	0.4	0.8	1.5	3.5	6.5	12.8

Temp		Days in Stage					
Mean	SE	Egg	L1	L2	L3f	L3m	P
10.4	0.16	6.5	6.8	5.6	9.7	29.0	100.8
12.7	0.13	1.3	4.3	3.9	8.4	64.1	38.3
15.1	0.15	2.2	3.6	1.7	5.6	18.3	39.8
17.5	0.09	1.9	1.7	2.1	4.2	3.7	18.1
20.1	0.1	1.2	1.2	1.4	4.0	1.2	14.0
22.5	0.90	0.9	1.0	1.3	2.7	2.6	9.3
25.0	0.08	0.7	0.9	1.0	2.0	2.4	8.4
27.5	0.07	0.5	0.8	0.7	3.2	1.4	7.8
30.0	0.23	0.5	0.5	0.7	1.9	2.2	6.6
32.5	0.07	0.4	0.4	0.7	2.0	3.0	6.3

Temp		1/Days to Stage Transition					
Mean	SE	E-L1	E-L2	E-L3f	E-L3m	E-P	E-A
10.4	0.51	0.155	0.076	0.053	0.035	0.017	0.006
12.7	0.18	0.742	0.176	0.104	0.056	0.012	0.008
15.1	0.14	0.465	0.174	0.134	0.076	0.032	0.014
17.5	0.12	0.526	0.281	0.176	0.101	0.074	0.032
20.1	0.09	0.857	0.414	0.261	0.127	0.111	0.043
22.5	0.10	1.084	0.525	0.310	0.170	0.119	0.057
25.0	0.06	1.418	0.624	0.385	0.216	0.143	0.065
27.5	0.06	2.028	0.778	0.516	0.193	0.152	0.070
30.0	0.05	2.098	0.975	0.577	0.277	0.172	0.081
32.5	0.02	2.470	1.250	0.675	0.284	0.153	0.078

Temp		1/Days in Stage					
Mean	SE	Egg	L1	L2	L3f	L3m	P
10.4	0.51	0.155	0.148	0.179	0.103	0.034	0.010
12.7	0.18	0.742	0.231	0.254	0.120	0.016	0.026
15.1	0.14	0.465	0.279	0.578	0.178	0.055	0.025
17.5	0.12	0.526	0.603	0.469	0.239	0.269	0.055
20.1	0.09	0.857	0.802	0.702	0.247	0.866	0.071
22.5	0.10	1.084	1.017	0.755	0.377	0.392	0.108
25.0	0.06	1.418	1.115	1.006	0.489	0.425	0.119
27.5	0.06	2.028	1.261	1.535	0.308	0.721	0.128
30.0	0.05	2.098	1.823	1.415	0.532	0.456	0.152
32.5	0.02	2.470	2.533	1.465	0.491	0.332	0.158

	Linear Regression Results (from Graph Pad Prism)					
	1/Days to Stage Transition					
	E-L1	E-L2	E-L3f	E-L3m	E-P	E-A
Dev Min (x-intercept):	12.6	10.8	10.5	8.8	10.3	10.7
ADD (1/slope):	8.2	21.3	35.2	82.5	107.5	230.2
Range min:	15.0	15.0	15.0	15.0	17.5	17.5
Range max:	30.0	30.0	30.0	30.0	30.0	30.0

	1/Days in Stage					
	Egg	L1	L2	L3f	L3m	P
Dev Min (x-intercept):	9.5	10.9	9.3	6.6	11.5	10.4
ADD (1/slope):	10.3	11.7	14.3	47.2	29.8	127.9
Range min:	15.0	15.0	15.0	17.5	17.5	17.5
Range max:	30.0	30.0	30.0	30.0	30.0	30.0

Comparison of Transition vs. Stage Results				
	trans	by stage	diff (t-s)	% diff (d/t)
Egg-L1	8.2	9.5	-1.3	-15.2%
L1-L2	21.3	20.4	1.0	4.5%
L2-L3f	35.2	34.6	0.6	1.8%
L3f-L3m	82.5	81.8	0.7	0.9%
L3m-P	107.5	111.6	-4.1	-3.8%
P-A	230.2	239.5	-9.3	-4.0%

From Prism (Linear Regressions): missing values represent non-linearity							
Temp		Transition ADD by 1/Day					
Mean	SE	E-L1	E-L2	E-L3f	E-L3m	E-P	E-A
10.4	0.51						
12.7	0.18	0.14	10.74	21.46	70.59		
15.1	0.14	5.33	24.45	34.36	82.36		
17.5	0.12	9.42	24.00	40.31	86.74	97.81	218.17
20.1	0.09	8.75	22.41	36.95	89.28	88.02	217.35
22.5	0.10	9.18	22.39	39.05	81.06	102.89	210.33
25.0	0.06	8.75	22.75	37.74	75.31	102.40	221.17
27.5	0.06	7.36	21.51	33.07	97.17	112.78	242.08
30.0	0.05	8.31	19.70	33.87	76.76	114.14	239.74
32.5	0.02	8.07	17.36	32.69	83.58		

Temp		Stage ADD by 1/Days					
Mean	SE	Egg	L1	L2	L3f	L3m	P
10.4	0.51						
12.7	0.18	4.3	7.8	13.2	50.8		
15.1	0.14	12.0	14.9	9.9	47.4		
17.5	0.12	15.3	11.0	17.5	45.8	22.3	129.8
20.1	0.09	12.4	11.5	15.3	54.5		136.2
22.5	0.10	12.1	11.5	17.5	42.2	28.1	112.7
25.0	0.06	10.9	12.6	15.6	37.5	31.6	123.4
27.5	0.06	8.9	13.2	11.8	67.7	22.2	133.6
30.0	0.05	9.8	10.5	14.6	44.0	40.5	129.3
32.5	0.02	9.3	8.5	15.8	52.7		

Temp (mean)	Transition ADD by 1/Day					
	E-L1	E-L2	E-L3f	E-L3m	E-P	E-A
10.4						
12.7						
15.1	5.3	24.4	34.4	82.4		
17.5	9.4	24.0	40.3	86.7	97.8	218.2
20.1	8.7	22.4	36.9	89.3	88.0	217.3
22.5	9.2	22.4	39.0	81.1	102.9	210.3
25.0	8.8	22.7	37.7	75.3	102.4	221.2
27.5	7.4	21.5	33.1	97.2	112.8	242.1
30.0	8.3	19.7	33.9	76.8	114.1	239.7
32.5						
**mean**	8.2	22.5	36.5	84.1	103.0	224.8
**SE**	1.3	1.5	2.6	7.1	8.9	11.9
**n**	7	7	7	7	6	6
**Regression ADD**	8	21	35	83	108	230
**% deviation**	-0.8%	5.4%	3.5%	1.9%	-4.2%	-2.3%
**ADD Range min:**	15.0	15.0	15.0	15.0	17.5	17.5
**ADD Range max:**	30.0	30.0	30.0	30.0	30.0	30.0

Temp (mean)	Stage ADD by 1/Days					
	Egg	L1	L2	L3f	L3m	P
10.4						
12.7						
15.1	12.0	14.9	9.9			
17.5	15.3	11.0	17.5	45.8	22.3	129.8
20.1	12.4	11.5	15.3	54.5		136.2
22.5	12.1	11.5	17.5	42.2	28.1	112.7
25.0	10.9	12.6	15.6	37.5	31.6	123.4
27.5	8.9	13.2	11.8	67.7	22.2	133.6
30.0	9.8	10.5	14.6	44.0	40.5	129.3
32.5						
**mean**	11.6	12.2	14.6	48.6	28.9	127.5
**SE**	1.9	1.4	2.6	9.9	6.8	7.7
**n**	7	7	7	6	5	6
**Regression ADD**	10	12	14	47	30	128
**% deviation**	13.3%	4.1%	2.4%	3.1%	-3.0%	-0.3%
**ADD Range min:**	15.0	15.0	15.0	17.5	17.5	17.5
**ADD Range max:**	30.0	30.0	30.0	30.0	30.0	30

Temp	% Time in Stage					
	Egg	L1	L2	L3f	L3m	P
10.4						
12.7	1.1%	3.6%	3.3%	7.0%	53.8%	32.2%
15.1	3.1%	5.2%	2.5%	8.1%	26.6%	57.6%
17.5	6.4%	5.6%	7.2%	14.1%	12.5%	60.7%
20.1	5.3%	5.7%	6.5%	18.5%	5.3%	64.0%
22.5	5.5%	5.9%	7.9%	15.8%	15.2%	55.2%
25.0	4.8%	6.1%	6.8%	13.9%	16.0%	57.3%
27.5	3.6%	5.7%	4.7%	23.4%	10.0%	56.2%
30.0	4.0%	4.6%	5.9%	15.8%	18.4%	55.3%
32.5	3.3%	3.2%	5.5%	16.4%	24.2%	50.8%
**Mean**	4.12%	5.06%	5.58%	14.77%	20.22%	54.37%
**SE**	1.49%	0.98%	1.69%	4.69%	13.42%	8.58%
**CV**	36.14%	19.37%	30.23%	31.76%	66.38%	15.78%

Temp		% Total Development					
Mean	SE	E-L1	E-L2	E-L3f	E-L3m	E-P	E-A
10.4	0.51						
12.7	0.18	0.57%	2.40%	4.07%	7.62%	34.82%	51.08%
15.1	0.14	1.67%	4.45%	5.79%	10.15%	24.38%	55.24%
17.5	0.12	2.95%	5.52%	8.83%	15.34%	21.11%	49.20%
20.1	0.09	2.52%	5.22%	8.30%	17.06%	19.55%	49.87%
22.5	0.10	2.48%	5.13%	8.70%	15.83%	22.71%	47.63%
25.0	0.06	2.26%	5.13%	8.31%	14.85%	22.37%	49.36%
27.5	0.06	1.68%	4.39%	6.61%	17.66%	22.39%	48.95%
30.0	0.05	1.94%	4.18%	7.05%	14.71%	23.63%	50.43%
32.5	0.02	1.61%	3.18%	5.89%	13.98%	25.94%	51.02%
	**Mean**	1.97%	4.40%	7.06%	14.13%	24.10%	50.31%
	**SE**	0.66%	0.97%	1.53%	3.06%	4.16%	2.02%
	**CV**	33.39%	22.13%	21.65%	21.69%	17.28%	4.02%

Temp		ADD Temp * Days to Stage Transition					
Mean	SE	E-L1	E-L2	E-L3f	E-L3m	E-P	E-A
10.4	0.51	66.9					
12.7	0.18	14.0	1.0	1.8	3.3	15.1	22.1
15.1	0.14	22.3	0.8	1.0	1.8	4.3	9.7
17.5	0.12	19.7	0.4	0.7	1.2	1.6	3.8
20.1	0.09	12.1	0.2	0.3	0.7	0.8	2.1
22.5	0.10	9.6	0.2	0.3	0.6	0.8	1.7
25.0	0.06	7.3	0.1	0.2	0.3	0.5	1.0
27.5	0.06	5.1	0.1	0.1	0.3	0.4	0.8
30.0	0.05	4.9	0.1	0.1	0.2	0.3	0.7
32.5	0.02	4.2	0.0	0.0	0.1	0.1	0.2

Temp		ADD Temp * Days in Stage					
Mean	SE	Egg	L1	L2	L3f	L3m	P
10.4	0.51						
12.7	0.18	0.2	0.8	0.7	1.5	11.8	7.0
15.1	0.14	0.3	0.5	0.2	0.8	2.5	5.4
17.5	0.12	0.2	0.2	0.3	0.5	0.4	2.2
20.1	0.09	0.1	0.1	0.1	0.4	0.1	1.3
22.5	0.10	0.1	0.1	0.1	0.3	0.2	0.9
25.0	0.06	0.0	0.1	0.1	0.1	0.2	0.5
27.5	0.06	0.0	0.0	0.0	0.2	0.1	0.4
30.0	0.05	0.0	0.0	0.0	0.1	0.1	0.4
32.5	0.02	0.0	0.0	0.0	0.0	0.0	0.1

For Deming Regressions

Temp		Temp * Days to Stage Transition					
Mean	SE	E-L1	E-L2	E-L3f	E-L3m	E-P	E-A
10.4	0.51	3.3	6.8	9.7	14.6	29.5	81.2
12.7	0.18	0.2	1.0	1.8	3.3	15.1	22.1
15.1	0.14	0.3	0.8	1.0	1.8	4.3	9.7
17.5	0.12	0.2	0.4	0.7	1.2	1.6	3.8
20.1	0.09	0.1	0.2	0.3	0.7	0.8	2.1
22.5	0.10	0.1	0.2	0.3	0.6	0.8	1.7
25.0	0.06	0.0	0.1	0.2	0.3	0.5	1.0
27.5	0.06	0.0	0.1	0.1	0.3	0.4	0.8
30.0	0.05	0.0	0.1	0.1	0.2	0.3	0.7
32.5	0.02	0.0	0.0	0.0	0.1	0.1	0.2

Temp		Temp * Days in Stage					
Mean	SE	Egg	L1	L2	L3f	L3m	P
10.4	0.51	3.3	3.5	2.9	5.0	14.9	51.7
12.7	0.18	0.2	0.8	0.7	1.5	11.8	7.0
15.1	0.14	0.3	0.5	0.2	0.8	2.5	5.4
17.5	0.12	0.2	0.2	0.3	0.5	0.4	2.2
20.1	0.09	0.1	0.1	0.1	0.4	0.1	1.3
22.5	0.10	0.1	0.1	0.1	0.3	0.2	0.9
25.0	0.06	0.0	0.1	0.1	0.1	0.2	0.5
27.5	0.06	0.0	0.0	0.0	0.2	0.1	0.4
30.0	0.05	0.0	0.0	0.0	0.1	0.1	0.4
32.5	0.02	0.0	0.0	0.0	0.0	0.0	0.1

Data for Prism polynomial fit for egg development

Temp	Egg
10.4	0.155
12.7	0.742
15.1	0.465
17.5	0.526
20.1	0.857
22.5	1.084
25.0	1.418
27.5	2.028
30.0	2.098
32.5	1.743

Supplementary Materials S2

Datasets and statistical analysis summary of *Lucilia sericata* nonlinear regressions for percent in stage by temperature. Regression equations are :

Gaussian equation (a standard normal curve):

$$y = a \exp\left[-\frac{1}{2}\left(\frac{x-b}{c}\right)^2\right]$$

Modified Gaussian equation (a form of Gaussian curve with a plateau at 100%):

$$y = a \exp\left[-\frac{1}{2}\left(\frac{|x-b|}{c}\right)^d\right]$$

Cumulative Gaussian equation (a form of the Gaussian curve used for adults, to model a sigmoidal increase to a plateau):

$$y = \frac{a}{2}\left[1 + erf\left(\frac{x-b}{\sqrt{2}c}\right)\right]$$

Reversed cumulative Gaussian equation (a form of the cumulative Gaussian equation used for eggs, to model a sigmoidal decrease from a plateau):

$$y = \frac{a}{2}\left[1 - erf\left(\frac{x-b}{\sqrt{2}c}\right)\right]$$

where x = development time (hours) and y = percent in stage. Where “interrupted” is indicated on a regression, data were insufficient to fit a curve (typically this means we obtained too few points in measuring portions of the transition curve). Where “ambiguous” is indicated on a regression, the program could not reach a final solution by iterative (numerical) methods. The analysis is from GraphPad Prism 6.0 which uses the Marquardt and Levenberg approach for non-linear regression.

L. sericata 10.0° C.

***L. sericata* 10.0° C**

Dataset

Time	egg Mean	egg SE	L1 Mean	L1 SE	L2 Mean	L2 SE	L3f Mean	L3f SE	L3m Mean	L3m SE	P Mean	P SE	A Mean	A SE
0.0	100	0	0	0	0	0	0	0	0	0	0	0	0	0
35.1	100	0	0	0	0	0	0	0	0	0	0	0	0	0
70.2	84.5	26.8	15.5	26.8	0	0	0	0	0	0	0	0	0	0
105.3	66.3	17.5	33.7	17.5	0	0	0	0	0	0	0	0	0	0
140.3	64.7	32	35.3	32	0	0	0	0	0	0	0	0	0	0
175.2	43.1	27.1	56.9	27.1	0	0	0	0	0	0	0	0	0	0
231.2	0	0	76.9	40	23.1	40	0	0	0	0	0	0	0	0
287.2	0	0	50	50	50	50	0	0	0	0	0	0	0	0
343.1	0	0	50	50	50	50	0	0	0	0	0	0	0	0
399.1	0	0	0	0	50	50	50	50	0	0	0	0	0	0
455.0	0	0	0	0	38.1	44.2	61.9	44.2	0	0	0	0	0	0
534.0	0	0	0	0	55	45.6	45	45.6	0	0	0	0	0	0
613.0	0	0	0	0	0	0	100	0	0	0	0	0	0	0
692.1	0	0	0	0	0	0	7.1	7.1	92.9	7.1	0	0	0	0
771.1	0	0	0	0	0	0	0	0	100	0	0	0	0	0
850.1	0	0	0	0	0	0	0	0	100	0	0	0	0	0
989.0	0	0	0	0	0	0	0	0	87.5	0	0	0	0	0
1128.0	0	0	0	0	0	0	0	0	50	50	50	50	0	0
1267.0	0	0	0	0	0	0	0	0	66.7	33.3	33.3	33.3	0	0
1406.0	0	0	0	0	0	0	0	0	87.5	12.5	12.5	12.5	0	0
1545.0	0	0	0	0	0	0	0	0	0	0	100	0	0	0
1880.0	0	0	0	0	0	0	0	0	60	40	20	20	20	20
2215.1	0	0	0	0	0	0	0	0	100	0	0	0	0	0
2885.1	0	0	0	0	0	0	0	0	0	0	100	0	0	0
3220.0	0	0	0	0	0	0	0	0	0	0	50	50	50	50
3747.0	0	0	0	0	0	0	0	0	0	0	88.9	15.7	11.1	15.7
4273.0	0	0	0	0	0	0	0	0	0	0	0	0	100	0
4273.0	0	0	0	0	0	0	0	0	0	0	0	0	100	0

Gaussian

	L1	L2	L3f	L3m	P
**Best-fit values**					
Amplitude	72.04	55.44	76.81	96.94	152
Mean	233.8	401.9	542.9	1015	1650
SD	88.9	124.6	107.2	297.2	114.4
**Std. Error**					
Amplitude	3.839	4.469	9.41	18.36	129.7
Mean	5.077	12.42	14.37	61.26	65.94
SD	5.115	11.74	14.25	61.21	48.25
**95% Confidence Intervals**					
Amplitude	64.13 to 79.95	46.24 to 64.65	57.43 to 96.19	59.11 to 134.8	-115.1 to 419.2
Mean	223.4 to 244.3	376.3 to 427.5	513.2 to 572.5	889.3 to 1142	1514 to 1786
SD	78.36 to 99.44	100.4 to 148.8	77.83 to 136.5	171.1 to 423.3	15.03 to 213.8
**Goodness of Fit**					
df	25	25	25	25	25
R square	0.9455	0.8722	0.7742	0.482	0.1177
Absolute SS	716.5	1275	3594	23150	23985
Sy.x	5.353	7.143	11.99	30.43	30.97
**Constraints**					
SD	SD > 0.0	SD > 0.0	SD > 0.0	SD > 0.0	SD > 0.0
Number of points					
Analyzed	28	28	28	28	28

Modified Gaussian

	L1	L3f
	Ambiguous	Ambiguous
**Best-fit values**		
a	35.37	~ 100.0
b	~ -551.8	~ 613.0
c	~ 943.8	~ 1.000
d	~ 435.2	~ 1.000
**Std. Error**		
a	5.36	~ 3.391e+011
b	~ 5.623e+021	~ 6.402e+029
c	~ 5.624e+021	~ 3.540e+030
d	~ 2.654e+021	~ 8.101e+029
**95% Confidence Intervals**		
a	24.30 to 46.43	(Very wide)
b	(Very wide)	(Very wide)
c	(Very wide)	(Very wide)
d	(Very wide)	(Very wide)
**Goodness of Fit**		
df	24	24
R square	0.5807	0.4719
Absolute SS	5516	8407
Sy.x	15.16	18.72
**Constraints**		
c	c > 0.0	c > 0.0
d	d > 1.000	d > 1.000
Number of points		
Analyzed	28	28

Cumulative Gaussian

	A
**Best-fit values**	
Mean	3757
SD	511.2
**Std. Error**	
Mean	105.4
SD	136.2
**95% Confidence Intervals**	
Mean	3540 to 3974
SD	231.1 to 791.3
**Goodness of Fit**	
df	26
R square	0.8213
Absolute SS	3609
Sy.x	11.78
Number of points	
Analyzed	28

Reversed Cumulative Gaussian

	egg
**Best-fit values**	
Mean	151.6
SD	66.18
**Std. Error**	
Mean	3.696
SD	5.504
**95% Confidence Intervals**	
Mean	144.0 to 159.2
SD	54.86 to 77.49
**Goodness of Fit**	
df	26
R square	0.9875
Absolute SS	375
Sy.x	3.798
**Runs test**	
Points above curve	4
Points below curve	10
Number of runs	4
P value (runs test)	0.0679
Deviation from Model	Not Significant
Number of points	
Analyzed	28

L. sericata 12.5° C.

***L. sericata* 12.5° C**														
	Dataset													
Time	egg		L1		L2		L3f		L3m		P		A	
	Mean	SE	Mean	SE	Mean	SE	Mean	SE	Mean	SE	Mean	SE	Mean	SE
0.0	100	0	0	0	0	0	0	0	0	0	0	0	0	0
11.0	100	0	0	0	0	0	0	0	0	0	0	0	0	0
46.2	22.1	22.3	77.9	22.3	0	0	0	0	0	0	0	0	0	0
81.1	22.6	10.6	77.4	10.6	0	0	0	0	0	0	0	0	0	0
116.2	16.4	8.2	46.4	35.8	37.2	37.2	0	0	0	0	0	0	0	0
151.1	29.2	29.2	2.1	3.6	68.8	27.9	0	0	0	0	0	0	0	0
207.1	0	0	2.3	3.9	72.7	42.2	25	43.3	0	0	0	0	0	0
263.1	0	0	0	0	12.7	15.9	87.3	15.9	0	0	0	0	0	0
319.1	0	0	0	0	27.5	42.1	72.5	42.1	0	0	0	0	0	0
375.1	0	0	0	0	6.3	10.8	93.8	10.8	0	0	0	0	0	0
431.1	0	0	0	0	0	0	52.1	48	47.9	48	0	0	0	0
510.1	0	0	0	0	0	0	0	0	100	0	0	0	0	0
589.0	0	0	0	0	0	0	0	0	100	0	0	0	0	0
668.1	0	0	0	0	0	0	0	0	100	0	0	0	0	0
747.1	0	0	0	0	0	0	0	0	100	0	0	0	0	0
826.1	0	0	0	0	0	0	0	0	95.9	4.3	4.1	4.3	0	0
965.1	0	0	0	0	0	0	0	0	86	1.7	14	1.7	0	0
1104.0	0	0	0	0	0	0	0	0	85.8	15.3	14.2	15.3	0	0
1243.0	0	0	0	0	0	0	0	0	96.5	3.5	3.5	3.5	0	0
1382.0	0	0	0	0	0	0	0	0	89.7	7.4	10.3	7.4	0	0
1521.0	0	0	0	0	0	0	0	0	71.3	33.7	23.3	24.7	5.4	9.3
1856.1	0	0	0	0	0	0	0	0	64.4	28.9	34.2	26.6	1.4	2.4
2191.1	0	0	0	0	0	0	0	0	14.1	24.4	69.4	20.3	16.5	16.8
2526.0	0	0	0	0	0	0	0	0	10.3	9.1	73	6.6	16.7	10.2
2861.0	0	0	0	0	0	0	0	0	11.7	13.6	48.6	15.7	39.7	24.9
3196.0	0	0	0	0	0	0	0	0	0	0	0	0	100	0
3196.0	0	0	0	0	0	0	0	0	0	0	0	0	100	0
3196.0	0	0	0	0	0	0	0	0	*12.500**	*21.700**	0	0	100	0
3196.0	0	0	0	0	0	0	0	0	*0.000**	*0.000**	0	0	100	0
3196.0	0	0	0	0	0	0	0	0	*5.600**	*7.900**	0	0	100	0
3196.0	0	0	0	0	0	0	0	0	*13.200**	*15.400**	0	0	100	0

Gaussian	L1	L2	L3f	L3m	P
**Best-fit values**					
Amplitude	90.67	86.28	95.07	109.6	79.6
Mean	72.97	179.8	333.8	1090	2335
SD	32.45	45.25	84	475.9	394.3
**Std. Error**					
Amplitude	4.541	5.954	4.958	10.14	5.412
Mean	1.879	3.078	5.201	57	30.16
SD	1.78	3.431	5.317	53.18	24.63
**95% Confidence Intervals**					
Amplitude	81.37 to 99.97	74.08 to 98.47	84.92 to 105.2	88.64 to 130.5	68.52 to 90.68
Mean	69.13 to 76.82	173.5 to 186.1	323.2 to 344.5	972.2 to 1207	2273 to 2397
SD	28.81 to 36.10	38.22 to 52.27	73.11 to 94.88	366.2 to 585.7	343.8 to 444.7
**Goodness of Fit**					
df	28	28	28	24	28
R square	0.9493	0.9199	0.9438	0.7831	0.8959
Absolute SS	651.2	859.3	1207	10971	1244
Sy.x	4.822	5.54	6.565	21.38	6.667
**Constraints**					
SD	SD > 0.0	SD > 0.0	SD > 0.0	SD > 0.0	SD > 0.0
Number of points					
Analyzed	31	31	31	27	31

Modified Gaussian	L1	L2	L3f	L3m
	Ambiguous	Ambiguous	Ambiguous	Ambiguous
**Best-fit values**				
a	77.65	~ 72.70	~ 93.80	92.52
b	~ 66.09	~ 207.1	~ 375.1	~ 1145
c	~ 50.07	~ 1.000	~ 1.000	~ 712.6
d	~ 42.12	~ 1.000	~ 1.000	~ 164.8
**Std. Error**				
a	0.4265	~ 5.021e+012	~ 1.992e+017	2.446
b	~ 3.055e+014	~ 4.554e+011	~ 1.951e+014	~ 2.108e+006
c	~ 3.096e+014	~ 2.230e+018	~ 2.551e+022	~ 162266
d	~ 5.174e+015	~ 5.541e+017	~ 6.338e+021	~ 2.692e+008
**95% Confidence Intervals**				
a	76.77 to 78.53	(Very wide)	(Very wide)	87.46 to 97.58
b	(Very wide)	(Very wide)	(Very wide)	(Very wide)
c	(Very wide)	(Very wide)	(Very wide)	(Very wide)
d	(Very wide)	(Very wide)	(Very wide)	(Very wide)
**Goodness of Fit**				
df	27	27	27	23
R square	0.9992	0.3403	0.2453	0.9755
Absolute SS	9.825	7075	16217	1239
Sy.x	0.6032	16.19	24.51	7.339
**Constraints**				
c	c > 0.0	c > 0.0	c > 0.0	c > 0.0
d	d > 1.000	d > 1.000	d > 1.000	d > 1.000
Number of points				
Analyzed	31	31	31	27

Cumulative Gaussian	A
**Best-fit values**	
Mean	2892
SD	124.1
**Std. Error**	
Mean	16.74
SD	42.29
**95% Confidence Intervals**	
Mean	2858 to 2926
SD	37.65 to 210.6
**Goodness of Fit**	
df	29
R square	0.9877
Absolute SS	580.2
Sy.x	4.473
Number of points	
Analyzed	31

Reversed Cumulative Gaussian	egg
**Best-fit values**	
Mean	39.5
SD	8.719
**Std. Error**	
Mean	64.9
SD	84.38
**95% Confidence Intervals**	
Mean	-93.22 to 172.2
SD	-163.8 to 181.3
**Goodness of Fit**	
Degrees of Freedom	29
R square	0.9159
Absolute SS	1632
Sy.x	7.503
**Runs test**	
Points above curve	5
Points below curve	1
Number of runs	3
P value (runs test)	1
Deviation from Model	Not Significant
Number of points	
Analyzed	31

L. sericata 15.0° C.

***L. sericata* 15.0° C**														
Dataset														
Time	egg		L1		L2		L3f		L3m		P		A	
	Mean	SE	Mean	SE	Mean	SE	Mean	SE	Mean	SE	Mean	SE	Mean	SE
0.0	100	0	0	0	0	0	0	0	0	0	0	0	0	0
17.2	100	0	0	0	0	0	0	0	0	0	0	0	0	0
34.2	100	0	0	0	0	0	0	0	0	0	0	0	0	0
51.2	51.8	19.3	48.2	19.3	0	0	0	0	0	0	0	0	0	0
68.2	6.8	11.8	93.2	11.8	0	0	0	0	0	0	0	0	0	0
97.3	0	0	100	0	0	0	0	0	0	0	0	0	0	0
113.2	0	0	100	0	0	0	0	0	0	0	0	0	0	0
141.1	0	0	4.2	7.2	95.8	7.2	0	0	0	0	0	0	0	0
169.1	0	0	0	0	72.9	42.2	27.1	42.2	0	0	0	0	0	0
197.1	0	0	0	0	16.2	4.6	83.8	4.6	0	0	0	0	0	0
225.1	0	0	0	0	0	0	100	0	0	0	0	0	0	0
264.1	0	0	0	0	0	0	100	0	0	0	0	0	0	0
303.1	0	0	0	0	0	0	93.8	10.8	6.3	10.8	0	0	0	0
318.0	0	0	0	0	0	0	25.9	29.2	74.1	29.2	0	0	0	0
381.1	0	0	0	0	0	0	0	0	100	0	0	0	0	0
444.1	0	0	0	0	0	0	0	0	95	8.7	5	8.7	0	0
491.1	0	0	0	0	0	0	0	0	97	4.3	3	4.3	0	0
564.6	0	0	0	0	0	0	0	0	83.7	12.1	16.3	12.1	0	0
633.0	0	0	0	0	0	0	0	0	61.5	19.9	38.5	19.9	0	0
704.1	0	0	0	0	0	0	0	0	65.7	14.3	34.3	14.3	0	0
775.0	0	0	0	0	0	0	0	0	95	8.7	5	8.7	0	0
942.2	0	0	0	0	0	0	0	0	56.3	39.9	15.1	1.1	28.6	40.4
1108.9	0	0	0	0	0	0	0	0	38.5	24.5	34.9	21.8	26.6	16.6
1277.6	0	0	0	0	0	0	0	0	32.6	20.5	56.2	24.7	11.2	14.9
1443.0	0	0	0	0	0	0	0	0	30.7	26.7	64.1	24.2	5.3	9.1
1610.1	0	0	0	0	0	0	0	0	23.9	23.9	51.5	21.9	24.6	29.5
1873.0	0	0	0	0	0	0	0	0	6.3	10.8	9.4	16.2	84.4	16.2
2135.0	0	0	0	0	0	0	0	0	0	0	0	0	100	0
2398.1	0	0	0	0	0	0	0	0	0	0	0	0	100	0
2664.5	0	0	0	0	0	0	0	0	0	0	0	0	100	0
2664.5	0	0	0	0	0	0	0	0	0	0	0	0	100	0

Gaussian						Modified Gaussian					Cumulative Gaussian		Reversed Cumulative Gaussian	
	L1	L2	L3f	L3m	P		L1	L2	L3f	L3m		A		egg
								Ambiguous						Ambiguous
Best-fit values						Best-fit values					Best-fit values		Best-fit values	
Amplitude	120.7	122.6	116.1	92.69	55.07	a	98.92	~ 95.80	94.63	81.77	Mean	1715	Mean	~ 61.40
Mean	89.52	152.8	249.3	675.2	1348	b	91.24	~ 141.1	243.5	708.8	SD	161.3	SD	~ 0.5224
SD	26.55	16.29	50.55	269.3	390.2	c	37.81	~ 1.001	71.33	398.2	Std. Error		Std. Error	
Std. Error						d	6.512	~ 1.000	21.86	87.09	Mean	29.42	Mean	~
Amplitude	5.753	6.717	6.388	10.25	5.366	Std. Error					SD	34	SD	~
Mean	1.304	0.515	2.785	40.7	47.87	a	0.8761	~ 45925	1.474	4.74	95% Confidence Intervals		95% Confidence Intervals	
SD	1.427	1.097	3.031	33.95	41.7	b	0.6863	~ 2.439e+006	0.185	2.277	Mean	1655 to 1775	Mean	(Very wide)
95% Confidence Intervals						c	0.9975	~ 1.617e+008	2.008	3.096	SD	91.77 to 230.8	SD	(Very wide)
Amplitude	109.0 to 132.5	108.8 to 136.4	103.0 to 129.2	71.70 to 113.7	44.08 to 66.06	d	0.9219		14.22	55.7	Goodness of Fit		Goodness of Fit	
Mean	86.85 to 92.19	151.8 to 153.9	243.6 to 255.0	591.9 to 758.6	1250 to 1446	95% Confidence Intervals					df	29	df	29
SD	23.63 to 29.48	14.04 to 18.53	44.34 to 56.76	199.7 to 338.8	304.8 to 475.6	a	97.12 to 100.7	(Very wide)	91.61 to 97.66	72.05 to 91.50	R square	0.9573	R square	0.9171
Goodness of Fit						b	89.83 to 92.65	(Very wide)	243.1 to 243.9	704.2 to 713.5	Absolute SS	1646	Absolute SS	2369
df	28	28	28	28	28	c	35.76 to 39.86	(Very wide)	67.21 to 75.45	391.8 to 404.5	Sy.x	7.533	Sy.x	9.039
R square	0.9608	0.9841	0.9433	0.6969	0.7889	d	4.620 to 8.404		1.000 to 51.04	1.000 to 201.4			Runs test	
Absolute SS	1065	217.2	1770	12469	2291	Goodness of Fit					Number of points		Points above curve	1
Sy.x	6.168	2.785	7.951	21.1	9.046	df	27	27	27	27	Analyzed	31	Points below curve	1
Constraints						R square	0.9992	0.5915	0.9944	0.882			Number of runs	2
SD	SD > 0.0	SD > 0.0	SD > 0.0	SD > 0.0	SD > 0.0	Absolute SS	22.16	5577	174.9	4852			P value (runs test)	1
						Sy.x	0.906	14.37	2.545	13.41			Deviation from Model	Not Significant
Number of points						Constraints								
Analyzed	31	31	31	31	31	c	c > 0.0	c > 0.0	c > 0.0	c > 0.0			Number of points	
						d	d > 1.000	d > 1.000	d > 1.000	d > 1.000			Analyzed	31
						Number of points								
						Analyzed	31	31	31	31				

L. sericata 17.5° C.

***L. sericata* 17.5° C**														
Dataset														
Time	egg		L1		L2		L3f		L3m		P		A	
	Mean	SE	Mean	SE	Mean	SE	Mean	SE	Mean	SE	Mean	SE	Mean	SE
0.0	100	0	0	0	0	0	0	0	0	0	0	0	0	0
12.1	100	0	0	0	0	0	0	0	0	0	0	0	0	0
24.2	100	0	0	0	0	0	0	0	0	0	0	0	0	0
36.2	55.8	21.5	44.2	21.5	0	0	0	0	0	0	0	0	0	0
48.3	54.8	19.4	45.2	19.4	0	0	0	0	0	0	0	0	0	0
60.2	65	28.3	35	28.3	0	0	0	0	0	0	0	0	0	0
79.1	0	0	91.2	15.3	8.8	15.3	0	0	0	0	0	0	0	0
98.2	0	0	1.4	2.4	98.6	2.4	0	0	0	0	0	0	0	0
117.1	0	0	0	0	93.8	10.8	6.3	10.8	0	0	0	0	0	0
136.1	0	0	0	0	53.1	47.1	46.9	47.1	0	0	0	0	0	0
155.1	0	0	0	0	0	0	100	0	0	0	0	0	0	0
181.1	0	0	0	0	0	0	100	0	0	0	0	0	0	0
207.1	0	0	0	0	0	0	93.8	10.8	6.3	10.8	0	0	0	0
233.1	0	0	0	0	0	0	57.1	31.6	42.9	31.6	0	0	0	0
259.1	0	0	0	0	0	0	17.5	20.5	82.5	20.5	0	0	0	0
285.2	0	0	0	0	0	0	0	0	97.2	4.8	2.8	4.8	0	0
333.1	0	0	0	0	0	0	0	0	30.4	34.2	69.6	34.2	0	0
381.1	0	0	0	0	0	0	0	0	26.4	35.7	73.6	35.7	0	0
429.1	0	0	0	0	0	0	0	0	6.3	10.8	93.8	10.8	0	0
477.1	0	0	0	0	0	0	0	0	10.7	11.8	89.3	11.8	0	0
525.1	0	0	0	0	0	0	0	0	29.9	31.8	70.1	31.8	0	0
635.0	0	0	0	0	0	0	0	0	5.8	6.1	94.2	6.1	0	0
745.1	0	0	0	0	0	0	0	0	6.7	9.4	49	38.4	44.3	38
855.0	0	0	0	0	0	0	0	0	16.7	16.7	0	0	83.3	16.7
965.1	0	0	0	0	0	0	0	0	0	0	0	0	100	0
1075.0	0	0	0	0	0	0	0	0	3.6	6.2	0	0	96.4	6.2
1268.2	0	0	0	0	0	0	0	0	0	0	0	0	100	0
1427.0	0	0	0	0	0	0	0	0	0	0	0	0	100	0
1603.0	0	0	0	0	0	0	0	0	0	0	0	0	100	0
1780.8	0	0	0	0	0	0	0	0	0	0	0	0	100	0
1923.5	0	0	0	0	0	0	0	0	0	0	0	0	100	0

Gaussian					
	L1	L2	L3	L3m	P
Best-fit values					
Amplitude	69.53	110.8	112.5	98.06	100.7
Mean	68.86	111.1	185.6	280.5	539.1
SD	20.41	18.08	37.58	35.81	151.5
Std. Error					
Amplitude	8.814	4.498	4.179	7.248	7.289
Mean	2.773	0.8477	1.534	3.514	13.25
SD	2.853	0.8529	1.524	3.061	11.74
95% Confidence Intervals					
Amplitude	51.48 to 87.58	101.6 to 120.0	104.0 to 121.1	83.21 to 112.9	85.80 to 115.7
Mean	63.18 to 74.54	109.3 to 112.8	182.4 to 188.7	273.3 to 287.7	512.0 to 566.2
SD	14.57 to 26.26	16.34 to 19.83	34.45 to 40.70	29.54 to 42.08	127.4 to 175.5
Goodness of Fit					
df	28	28	28	28	28
R square	0.7551	0.9671	0.9707	0.8773	0.8875
Absolute SS	2944	636.9	845.5	2069	3795
Sy.x	10.25	4.769	5.495	8.596	11.64
Constraints					
SD	SD > 0.0	SD > 0.0	SD > 0.0	SD > 0.0	SD > 0.0
Number of points					
Analyzed	31	31	31	31	31

Modified Gaussian					
	L1	L2	L3	L3m	P
	Hit constraint	Ambiguous			
Best-fit values					
a	119.3	96.2	101.5	102.6	94.2
b	73.47	~ 108.9	186.6	279.7	414.9
c	7.883	~ 26.94	45.85	31.39	9.889
d	~ 1.000	~ 15.67	3.631	1.556	1
Std. Error					
a	87.66	0.6647	2.419	12.06	3149
b	2.543	~ 2892	0.7942	4.164	101.2
c	11	~ 3539	1.185	8.701	463.5
d		~ 35868	0.3222	0.515	19.93
95% Confidence Intervals					
a	-60.58 to 299.2	94.84 to 97.56	96.58 to 106.5	77.81 to 127.3	-6368 to 6556
b	68.26 to 78.69	(Very wide)	184.9 to 188.2	271.2 to 288.3	207.1 to 622.6
c	0.0 to 30.45	(Very wide)	43.42 to 48.28	13.54 to 49.25	0.0 to 961.1
d		(Very wide)	2.970 to 4.292	1.000 to 2.613	1.000 to 41.90
Goodness of Fit					
df	27	27	27	27	27
R square	0.7916	0.9994	0.9902	0.8789	0.005956
Absolute SS	2506	11.52	282.1	2043	33539
Sy.x	9.633	0.6532	3.232	8.698	35.24
Constraints					
c	c > 0.0	c > 0.0	c > 0.0	c > 0.0	c > 0.0
d	d > 1.000	d > 1.000	d > 1.000	d > 1.000	d > 1.000
Number of points					
Analyzed	31	31	31	31	31

Cumulative Gaussian	
	A
Best-fit values	
Mean	764.2
SD	82.17
Std. Error	
Mean	2.771
SD	4.003
95% Confidence Intervals	
Mean	758.6 to 769.9
SD	73.99 to 90.36
Goodness of Fit	
df	29
R square	0.9988
Absolute SS	69.74
Sy.x	1.551
Number of points	
Analyzed	31

Reversed Cumulative Gaussian	
	egg
Best-fit values	
Mean	54.12
SD	24.41
Std. Error	
Mean	2.495
SD	3.671
95% Confidence Intervals	
Mean	49.02 to 59.22
SD	16.90 to 31.92
Goodness of Fit	
df	29
R square	0.9555
Absolute SS	1470
Sy.x	7.12
Runs test	
Points above curve	4
Points below curve	10
Number of runs	4
P value (runs test)	0.0679
Deviation from Model	Not Significant
Number of points	
Analyzed	31

L. sericata 20.0° C.

L. sericata 20.0° C

Dataset

Time	egg Mean	egg SE	L1 Mean	L1 SE	L2 Mean	L2 SE	L3f Mean	L3f SE	L3m Mean	L3m SE	P Mean	P SE	A Mean	A SE
0.0	100	0	0	0	0	0	0	0	0.000*	0.000*	0	0	0	0
9.1	100	0	0	0	0	0	0	0	0.000*	0.000*	0	0	0	0
18.1	100	0	0	0	0	0	0	0	0.000*	0.000*	0	0	0	0
27.3	71.4	17.7	28.6	17.7	0	0	0	0	0.000*	0.000*	0	0	0	0
36.1	77.3	20.7	22.7	20.7	0	0	0	0	0.000*	0.000*	0	0	0	0
45.2	65.8	23.5	34.2	23.5	0	0	0	0	0.000*	0.000*	0	0	0	0
59.1	0	0	12.5	21.7	87.5	21.7	0	0	0.000*	0.000*	0	0	0	0
73.2	0	0	0	0	100	0	0	0	0.000*	0.000*	0	0	0	0
87.1	0	0	0	0	88.7	15.5	11.3	15.5	0.000*	0.000*	0	0	0	0
101.1	0	0	0	0	3.1	5.4	96.9	5.4	0.000*	0.000*	0	0	0	0
115.1	0	0	0	0	0	0	100	0	0.000*	0.000*	0	0	0	0
135.1	0	0	0	0	0	0	100	0	0.000*	0.000*	0	0	0	0
155.1	0	0	0	0	0	0	100	0	0.000*	0.000*	0	0	0	0
175.1	0	0	0	0	0	0	100	0	0	0	0	0	0	0
195.0	0	0	0	0	0	0	9.4	16.2	90.6	16.2	0	0	0	0
215.0	0	0	0	0	0	0	0	0	87.5	21.7	12.5	21.7	0	0
251.0	0	0	0	0	0	0	0	0	0	0	100	0	0	0
287.0	0	0	0	0	0	0	0	0	0	0	100	0	0	0
323.0	0	0	0	0	0	0	0	0	0.000*	0.000*	100	0	0	0
359.0	0	0	0	0	0	0	0	0	0.000*	0.000*	100	0	0	0
395.0	0	0	0	0	0	0	0	0	0.000*	0.000*	100	0	0	0
479.0	0	0	0	0	0	0	0	0	0.000*	0.000*	100	0	0	0
563.0	0	0	0	0	0	0	0	0	0.000*	0.000*	10	17.3	90	17.3
647.0	0	0	0	0	0	0	0	0	0.000*	0.000*	0	0	100	0
731.0	0	0	0	0	0	0	0	0	0.000*	0.000*	3.7	3.7	96.3	3.7
815.0	0	0	0	0	0	0	0	0	0.000*	0.000*	0	0	100	0
815.0	0	0	0	0	0	0	0	0	0.000*	0.000*	0	0	100	0
815.0	0	0	0	0	0	0	0	0	0.000*	0.000*	0	0	100	0
815.0	0	0	0	0	0	0	0	0	0.000*	0.000*	0	0	100	0
815.0	0	0	0	0	0	0	0	0	0.000*	0.000*	0	0	100	0
815.0	0	0	0	0	0	0	0	0	0.000*	0.000*	0	0	100	0

Gaussian

	L1	L2	L3	L3m Interrupted	P
**Best-fit values**					
Amplitude	32.59	115.4	120.7	1147	118.2
Mean	41.17	73.37	139.4	205	378.8
SD	13.08	14.37	33.21	4.423	102.6
**Std. Error**					
Amplitude	2.206	6.131	8.594		8.421
Mean	1.131	0.8848	2.578		9.715
SD	1.069	0.8646	2.58		8.316
**95% Confidence Intervals**					
Amplitude	28.08 to 37.11	102.8 to 127.9	103.1 to 138.3		101.0 to 135.5
Mean	38.85 to 43.49	71.56 to 75.18	134.1 to 144.7		358.9 to 398.7
SD	10.89 to 15.27	12.60 to 16.14	27.92 to 38.49		85.57 to 119.6
**Goodness of Fit**					
df	28	28	28		28
R square	0.9065	0.9438	0.899		0.8859
Absolute SS	219.7	1294	4139		5435
Sy.x	2.801	6.799	12.16		13.93
**Constraints**					
SD	SD > 0.0	SD > 0.0	SD > 0.0	SD > 0.0	SD > 0.0
Number of points					
Analyzed	31	31	31	5	31

Modified Gaussian

	L1 Ambiguous	L2	L3	L3m Interrupted	P Ambiguous
**Best-fit values**					
a	28.5	99.94	100.1	9583	100
b	~ 39.28	73.35	140.9	205	~ 388.9
c	~ 19.60	18.42	48.43	1.069	~ 171.4
d	~ 43.39	5.038	14.03	~ 1.000	~ 96.83
**Std. Error**					
a	1.107	0.3725	0.03356		0.3184
b	~ 6.665e+006	0.06358	0.007575		~ 86811
c	~ 8.810e+006	0.1011	0.03018		~ 2.476e+006
d	~ 4.270e+008	0.07768	0.07961		~ 9.178e+007
**95% Confidence Intervals**					
a	26.23 to 30.77	99.18 to 100.7	100.0 to 100.2		99.35 to 100.7
b	(Very wide)	73.22 to 73.48	140.9 to 140.9		(Very wide)
c	(Very wide)	18.21 to 18.63	48.37 to 48.50		(Very wide)
d	(Very wide)	4.879 to 5.197	13.87 to 14.20		(Very wide)
**Goodness of Fit**					
df	27	27	27		27
R square	0.9718	0.9998	1		0.9997
Absolute SS	66.14	3.75	0.1103		13.69
Sy.x	1.565	0.3727	0.06391		0.7121
**Constraints**					
c	c > 0.0	c > 0.0	c > 0.0	c > 0.0	c > 0.0
d	d > 1.000	d > 1.000	d > 1.000	d > 1.000	d > 1.000
Number of points					
Analyzed	31	31	31	5	31

Cumulative Gaussian

	A Ambiguous
**Best-fit values**	
Mean	~ 546.2
SD	~ 13.09
**Std. Error**	
Mean	~ 23306
SD	~ 18184
**95% Confidence Intervals**	
Mean	(Very wide)
SD	(Very wide)
**Goodness of Fit**	
df	29
R square	0.9998
Absolute SS	13.69
Sy.x	0.6871
Number of points	
Analyzed	31

Reversed Cumulative Gaussian

	egg
**Best-fit values**	
Mean	45.36
SD	13.15
**Std. Error**	
Mean	1.263
SD	1.78
**95% Confidence Intervals**	
Mean	42.78 to 47.95
SD	9.504 to 16.79
**Goodness of Fit**	
df	29
R square	0.9765
Absolute SS	867
Sy.x	5.468
**Runs test**	
Points above curve	5
Points below curve	7
Number of runs	4
P value (runs test)	0.0758
Deviation from Model	Not Significant
Number of points	
Analyzed	31

L. sericata 22.5 C.

L. sericata 22.5° C

Time	egg		L1		L2		L3f		L3m		P		A	
	Mean	SE	Mean	SE	Mean	SE	Mean	SE	Mean	SE	Mean	SE	Mean	SE
0.0	100	0	0	0	0	0	0	0	0	0	0	0	0	0
7.1	100	0	0	0	0	0	0	0	0	0	0	0	0	0
14.2	95	5	5	5	0	0	0	0	0	0	0	0	0	0
21.2	64.6	20.5	35.4	20.5	0	0	0	0	0	0	0	0	0	0
28.2	2.5	4.3	97.5	4.3	0	0	0	0	0	0	0	0	0	0
35.1	8.3	14.4	91.7	14.4	0	0	0	0	0	0	0	0	0	0
46.1	0	0	28.6	30.9	71.4	30.9	0	0	0	0	0	0	0	0
57.1	0	0	0	0	100	0	0	0	0	0	0	0	0	0
68.1	0	0	0	0	100	0	0	0	0	0	0	0	0	0
79.1	0	0	0	0	35.3	24.9	64.7	24.9	0	0	0	0	0	0
90.1	0	0	0	0	5.2	5.3	94.8	5.3	0	0	0	0	0	0
106.1	0	0	0	0	0	0	100	0	0	0	0	0	0	0
122.1	0	0	0	0	0	0	100	0	0	0	0	0	0	0
138.1	0	0	0	0	0	0	40.6	26.8	59.4	26.8	0	0	0	0
154.2	0	0	0	0	0	0	33.3	47.1	66.7	47.1	0	0	0	0
170.1	0	0	0	0	0	0	15	15	85	15	0	0	0	0
198.1	0	0	0	0	0	0	21.8	10.9	41.7	31.2	36.5	27.6	0	0
226.1	0	0	0	0	0	0	0	0	5.6	5.6	94.4	5.6	0	0
254.0	0	0	0	0	0	0	0	0	0	0	100	0	0	0
282.0	0	0	0	0	0	0	0	0	0	0	100	0	0	0
310.0	0	0	0	0	0	0	0	0	0	0	100	0	0	0
377.0	0	0	0	0	0	0	0	0	0	0	100	0	0	0
443.5	0	0	0	0	0	0	0	0	0	0	3.3	3.3	96.7	3.3
511.0	0	0	0	0	0	0	0	0	0	0	2	2.8	98	2.8
578.0	0	0	0	0	0	0	0	0	0	0	6.7	4.1	93.3	4.1
651.0	0	0	0	0	0	0	0	0	0	0	0	0	100	0
651.0	0	0	0	0	0	0	0	0	0	0	0	0	100	0
651.0	0	0	0	0	0	0	0	0	0	0	0	0	100	0
651.0	0	0	0	0	0	0	0	0	0	0	0	0	100	0
651.0	0	0	0	0	0	0	0	0	0	0	0	0	100	0
651.0	0	0	0	0	0	0	0	0	0	0	0	0	100	0

Gaussian

	L1	L2	L3	L3m	P
**Best-fit values**					
Amplitude	103.4	113.5	112.1	85.31	118.6
Mean	32.29	60.54	108.7	167.6	308.2
SD	8.003	12.45	23.44	24.89	73.45
**Std. Error**					
Amplitude	1.858	3.709	6.731	3.8	7.091
Mean	0.1982	0.4702	1.533	1.387	5.569
SD	0.1808	0.4637	1.524	1.287	4.951
**95% Confidence Intervals**					
Amplitude	99.63 to 107.2	105.9 to 121.1	98.35 to 125.9	77.53 to 93.09	104.1 to 133.2
Mean	31.88 to 32.69	59.58 to 61.50	105.5 to 111.8	164.8 to 170.5	296.8 to 319.6
SD	7.633 to 8.373	11.50 to 13.40	20.32 to 26.57	22.25 to 27.52	63.31 to 83.59
**Goodness of Fit**					
df	28	28	28	28	28
R square	0.9926	0.9777	0.9243	0.9581	0.9229
Absolute SS	131.9	519	2233	620.4	3144
Sy.x	2.17	4.305	8.931	4.707	10.6
**Constraints**					
SD	SD > 0.0	SD > 0.0	SD > 0.0	SD > 0.0	SD > 0.0
Number of points					
Analyzed	31	31	31	31	31

Modified Gaussian

	L1	L2	L3	L3m	P
	Ambiguous	Ambiguous			
**Best-fit values**					
a	94.6	100	98.83	75.93	100.1
b	~ 33.57	~ 61.49	107.1	166.1	313.5
c	~ 11.85	16.13	28.63	31.12	107.8
d	~ 15.80	~ 8.352	7.323	6.727	10.27
**Std. Error**					
a	1.245	1.035	5.245	1.925	0.7012
b	~ 59.24	~ 6.135	1.448	0.9176	1.122
c	~ 445.1	2.252	1.289	0.684	1.69
d	~ 12091	~ 46.30	6.663	2.862	1.088
**95% Confidence Intervals**					
a	92.05 to 97.15	97.89 to 102.1	88.07 to 109.6	71.98 to 79.88	98.64 to 101.5
b	(Very wide)	(Very wide)	104.2 to 110.1	164.2 to 168.0	311.2 to 315.8
c	(Very wide)	11.51 to 20.75	25.99 to 31.28	29.72 to 32.52	104.3 to 111.3
d	(Very wide)	(Very wide)	1.000 to 21.00	1.000 to 12.60	8.033 to 12.50
**Goodness of Fit**					
df	27	27	27	27	27
R square	0.9977	0.9988	0.9382	0.9866	0.9988
Absolute SS	41.82	27.04	1822	197.9	48.92
Sy.x	1.245	1.001	8.216	2.707	1.346
**Constraints**					
c	c > 0.0	c > 0.0	c > 0.0	c > 0.0	c > 0.0
d	d > 1.000	d > 1.000	d > 1.000	d > 1.000	d > 1.000
Number of points					
Analyzed	31	31	31	31	31

Cumulative Gaussian

	A
**Best-fit values**	
Mean	~ 424.1
SD	~ 10.53
Std. Error	
Mean	~ 2193
SD	~ 1193
**95% Confidence Intervals**	
Mean	(Very wide)
SD	(Very wide)
**Goodness of Fit**	
df	29
R square	0.9992
Absolute SS	48.89
Sy.x	1.298
Number of points	
Analyzed	31

Reversed Cumulative Gaussian

	egg
**Best-fit values**	
Mean	22.37
SD	3.204
Std. Error	
Mean	0.1757
SD	0.338
**95% Confidence Intervals**	
Mean	22.01 to 22.73
SD	2.513 to 3.895
**Goodness of Fit**	
df	29
R square	0.9969
Absolute SS	89.73
Sy.x	1.759
**Runs test**	
Points above curve	4
Points below curve	3
Number of runs	6
P value (runs test)	0.9714
Deviation from Model	Not Significant
Number of points	
Analyzed	31

L. sericata 25.0° C.

***L. sericata* 25.0° C** — Dataset

Time	egg Mean	egg SE	L1 Mean	L1 SE	L2 Mean	L2 SE	L3f Mean	L3f SE	L3m Mean	L3m SE	P Mean	P SE	A Mean	A SE
0.0	100	0	0	0	0	0	0	0	0	0	0	0	0	0
6.1	100	0	0	0	0	0	0	0	0	0	0	0	0	0
12.1	100	0	0	0	0	0	0	0	0	0	0	0	0	0
18.3	29.3	23.9	70.7	23.9	0	0	0	0	0	0	0	0	0	0
24.2	5.2	3.3	94.8	3.3	0	0	0	0	0	0	0	0	0	0
30.1	6.6	4.4	93.4	4.4	0	0	0	0	0	0	0	0	0	0
39.2	0	0	12.5	21.7	87.5	21.7	0	0	0	0	0	0	0	0
48.3	0	0	0	0	100	0	0	0	0	0	0	0	0	0
57.1	0	0	0	0	84.8	22.5	15.2	22.5	0	0	0	0	0	0
66.1	0	0	0	0	23.8	10.6	76.2	10.6	0	0	0	0	0	0
75.1	0	0	0	0	0	0	100	0	0	0	0	0	0	0
88.1	0	0	0	0	0	0	100	0	0	0	0	0	0	0
101.0	0	0	0	0	0	0	100	0	0	0	0	0	0	0
114.1	0	0	0	0	0	0	18.8	15.3	81.2	15.3	0	0	0	0
127.1	0	0	0	0	0	0	2.8	4.8	97.2	4.8	0	0	0	0
140.3	0	0	0	0	0	0	1.6	2.7	98.4	2.7	0	0	0	0
164.3	0	0	0	0	0	0	0	0	59.9	24.1	40.1	24.1	0	0
188.1	0	0	0	0	0	0	0	0	9.2	6.1	90.8	6.1	0	0
212.1	0	0	0	0	0	0	0	0	5.4	5.9	94.6	5.9	0	0
236.0	0	0	0	0	0	0	0	0	0	0	100	0	0	0
260.0	0	0	0	0	0	0	0	0	3.1	5.4	96.9	5.4	0	0
316.0	0	0	0	0	0	0	0	0	0	0	100	0	0	0
372.0	0	0	0	0	0	0	0	0	0	0	46.6	24.5	53.4	24.5
428.0	0	0	0	0	0	0	0	0	0	0	3.1	3.1	96.9	3.1
484.0	0	0	0	0	0	0	0	0	0	0	6.9	8.8	93.1	8.8
540.0	0	0	0	0	0	0	0	0	0	0	0	0	100	0
540.0	0	0	0	0	0	0	0	0	0	0	0	0	100	0
540.0	0	0	0	0	0	0	0	0	0	0	0	0	100	0
540.0	0	0	0	0	0	0	0	0	0	0	0	0	100	0
540.0	0	0	0	0	0	0	0	0	0	0	0	0	100	0
540.0	0	0	0	0	0	0	0	0	0	0	0	0	100	0

Gaussian

	L1	L2	L3	L3m	P
**Best-fit values**					
Amplitude	107	112	118.4	109.1	116.9
Mean	25.86	48.76	86	138.4	269.3
SD	6.825	9.867	17.02	21.81	70.5
**Std. Error**					
Amplitude	3.563	4.864	5.608	5.564	6.253
Mean	0.2986	0.4977	0.8739	1.383	4.487
SD	0.301	0.4869	0.88	1.287	4.066
**95% Confidence Intervals**					
Amplitude	99.70 to 114.3	102.1 to 122.0	107.0 to 129.9	97.75 to 120.5	104.0 to 129.7
Mean	25.24 to 26.47	47.74 to 49.78	84.21 to 87.79	135.5 to 141.2	260.1 to 278.5
SD	6.209 to 7.442	8.869 to 10.86	15.22 to 18.82	19.17 to 24.44	62.17 to 78.83
**Goodness of Fit**					
df	28	28	28	28	28
R square	0.9764	0.9613	0.9547	0.9456	0.9375
Absolute SS	482.8	874.8	1399	1380	2474
Sy.x	4.152	5.59	7.067	7.02	9.401
**Constraints**					
SD	SD > 0.0	SD > 0.0	SD > 0.0	SD > 0.0	SD > 0.0
Number of points					
Analyzed	31	31	31	31	31

Modified Gaussian

	L1	L2	L3	L3m	P
	Ambiguous	Ambiguous			
**Best-fit values**					
a	94.15	93.49	101.7	97.81	98.01
b	27.01	49.58	86.15	137.8	269.2
c	9.584	14	22.45	26.61	98.6
d	5.795	6.173	5.401	8.376	9.427
**Std. Error**					
a	0.1379	1.546	0.7683	1.522	0.8625
b	0.1274	0.277	0.1174	2.121	0.3694
c	0.07418	0.3318	0.1886	2.213	0.6682
d	0.4336	0.7666	0.1951	12.26	1.012
**95% Confidence Intervals**					
a	93.87 to 94.44	90.32 to 96.66	100.2 to 103.3	94.69 to 100.9	96.24 to 99.78
b	26.74 to 27.27	49.01 to 50.15	85.91 to 86.39	133.4 to 142.1	268.4 to 269.9
c	9.432 to 9.736	13.32 to 14.68	22.06 to 22.83	22.07 to 31.15	97.23 to 99.97
d	4.905 to 6.685	4.600 to 7.746	5.001 to 5.801	1.000 to 33.53	7.350 to 11.50
**Goodness of Fit**					
df	27	27	27	27	27
R square	1	0.9953	0.999	0.9951	0.9981
Absolute SS	0.9612	106.9	32.11	124.1	76.43
Sy.x	0.1887	1.99	1.091	2.144	1.682
**Constraints**					
c	c > 0.0	c > 0.0	c > 0.0	c > 0.0	c > 0.0
d	d > 1.000	d > 1.000	d > 1.000	d > 1.000	d > 1.000
Number of points					
Analyzed	31	31	31	31	31

Cumulative Gaussian

	A
**Best-fit values**	
Mean	370.1
SD	24.86
**Std. Error**	
Mean	0.8794
SD	3.293
**95% Confidence Intervals**	
Mean	368.3 to 371.9
SD	18.13 to 31.60
**Goodness of Fit**	
df	29
R square	0.9991
Absolute SS	54.35
Sy.x	1.369
Number of points	
Analyzed	31

Reversed Cumulative Gaussian

	egg
**Best-fit values**	
Mean	17.3
SD	1.84
**Std. Error**	
Mean	0.6314
SD	1.153
**95% Confidence Intervals**	
Mean	16.01 to 18.59
SD	-0.5183 to 4.19[illegible]
**Goodness of Fit**	
Degrees of Freedom	29
R square	0.9974
Absolute SS	70.56
Sy.x	1.56
**Runs test**	
Points above curve	4
Points below curve	1
Number of runs	3
P value (runs test)	1
Deviation from Model	Not Significant
Number of points	
Analyzed	31

L. sericata 27.5° C.

L. sericata 27.5° C

Time	egg Mean	egg SE	L1 Mean	L1 SE	L2 Mean	L2 SE	L3f Mean	L3f SE	L3m Mean	L3m SE	P Mean	P SE	A Mean	A SE
0	100	0	0	0	0	0	0	0	0	0	0	0	0	0
5.1	100	0	0	0	0	0	0	0	0	0	0	0	0	0
10.1	51.1	30.2	48.9	30.2	0	0	0	0	0	0	0	0	0	0
15.3	29.9	36	70.1	36	0	0	0	0	0	0	0	0	0	0
20.3	33.1	25.5	66.9	25.5	0	0	0	0	0	0	0	0	0	0
25.2	14.6	9.6	85.4	9.6	0	0	0	0	0	0	0	0	0	0
33.2	0	0	10.7	5.5	89.3	5.5	0	0	0	0	0	0	0	0
41.2	0	0	3.9	4.2	96.1	4.2	0	0	0	0	0	0	0	0
49.2	0	0	0	0	1.5	2.5	98.5	2.5	0	0	0	0	0	0
57.1	0	0	0	0	8.3	10.2	91.7	10.2	0	0	0	0	0	0
65.1	0	0	0	0	1.6	2.7	98.4	2.7	0	0	0	0	0	0
76.1	0	0	0	0	2.8	4.8	97.2	4.8	0	0	0	0	0	0
87.1	0	0	0	0	0	0	100	0	0	0	0	0	0	0
98.1	0	0	0	0	0	0	100	0	0	0	0	0	0	0
110.1	0	0	0	0	0	0	100	0	0	0	0	0	0	0
121	0	0	0	0	0	0	62.5	41.5	37.5	41.5	0	0	0	0
141.1	0	0	0	0	0	0	6.3	10.8	90.6	10.4	3.1	5.4	0	0
161.1	0	0	0	0	0	0	0	0	35	23.7	65	23.7	0	0
181	0	0	0	0	0	0	4.2	7.2	8.3	14.4	87.5	21.7	0	0
201	0	0	0	0	0	0	0	0	0	0	100	0	0	0
221	0	0	0	0	0	0	0	0	0	0	100	0	0	0
269.1	0	0	0	0	0	0	0	0	0	0	100	0	0	0
317	0	0	0	0	0	0	0	0	0	0	75	30.6	25	30.6
365	0	0	0	0	0	0	0	0	16.7	23.6	16.7	23.6	66.7	47.1
413	0	0	0	0	0	0	0	0	0	0	0	0	100	0
461	0	0	0	0	0	0	0	0	0	0	0	0	100	0
461	0	0	0	0	0	0	0	0	0	0	0	0	100	0
461	0	0	0	0	0	0	0	0	0	0	0	0	100	0
461	0	0	0	0	0	0	0	0	0	0	0	0	100	0
461	0	0	0	0	0	0	0	0	0	0	0	0	100	0
461	0	0	0	0	0	0	0	0	0	0	0	0	100	0

Gaussian

	L1	L2	L3	L3m	P
Best-fit values					
Amplitude	83.21	167.4	117	91.35	120.3
Mean	20.28	37.32	85.61	141.3	245.5
SD	7.864	3.679	28.77	14.33	61.29
Std. Error					
Amplitude	4.436	30.27	8.105	3.789	6.507
Mean	0.5489	0.05786	2.326	0.7781	3.581
SD	0.5365	0.5615	2.338	0.5912	3.446
95% Confidence Intervals					
Amplitude	74.13 to 92.29	105.4 to 229.4	100.4 to 133.6	83.58 to 99.11	106.9 to 133.6
Mean	19.16 to 21.41	37.21 to 37.44	80.84 to 90.37	139.7 to 142.9	238.2 to 252.8
SD	6.765 to 8.962	2.529 to 4.829	23.98 to 33.56	13.12 to 15.54	54.24 to 68.35
Goodness of Fit					
df	28	28	28	28	28
R square	0.9384	0.995	0.8913	0.9592	0.9411
Absolute SS	1021	80.17	5719	409.5	2248
Sy.x	6.039	1.692	14.29	3.824	8.96
Constraints					
SD	SD > 0.0	SD > 0.0	SD > 0.0	SD > 0.0	SD > 0.0
Number of points					
Analyzed	31	31	31	31	31

Modified Gaussian

	L1	L2	L3	L3m	P
	Ambiguous	Ambiguous			
Best-fit values					
a	74.16	~ 962.6	97.97	90.6	98.24
b	20.7	37.26	~ 81.52	~ 140.9	241.4
c	10.81	~ 0.8543	~ 39.51	~ 18.37	81.36
d	~ 9.333	~ 1.000	~ 177.8	~ 6.915	8.464
Std. Error					
a	2.385	~ 60920	1.222	3.589	1.921
b	11.66	0.6686	~ 3.324e+014	~ 5216	1.071
c	9.344	~ 44.73	~ 3.137e+014	~ 76857	1.103
d	~ 115.6		~ 1.420e+017	~ 330532	1.113
95% Confidence Intervals					
a	69.27 to 79.05	(Very wide)	95.46 to 100.5	83.24 to 97.96	94.30 to 102.2
b	-3.218 to 44.62	35.89 to 38.63	(Very wide)	(Very wide)	239.2 to 243.6
c	0.0 to 29.98	(Very wide)	(Very wide)	(Very wide)	79.10 to 83.62
d	(Very wide)		(Very wide)	(Very wide)	6.181 to 10.75
Goodness of Fit					
df	27	27	27	27	27
R square	0.9873	0.995	0.9979	0.9654	0.9909
Absolute SS	210.5	80.2	110.1	347.8	345.1
Sy.x	2.792	1.724	2.019	3.589	3.575
Constraints					
c	c > 0.0	c > 0.0	c > 0.0	c > 0.0	c > 0.0
d	d > 1.000	d > 1.000	d > 1.000	d > 1.000	d > 1.000
Number of points					
Analyzed	31	31	31	31	31

Cumulative Gaussian

	A
Best-fit values	
Mean	345.7
SD	38.65
Std. Error	
Mean	0.8633
SD	1.195
95% Confidence Intervals	
Mean	343.9 to 347.4
SD	36.21 to 41.10
Goodness of Fit	
df	29
R square	0.9994
Absolute SS	32.79
Sy.x	1.063
Number of points	
Analyzed	31

Reversed Cumulative Gaussian

	egg
Best-fit values	
Mean	13.34
SD	8.528
Std. Error	
Mean	0.6601
SD	0.9593
95% Confidence Intervals	
Mean	11.99 to 14.69
SD	6.567 to 10.49
Goodness of Fit	
df	29
R square	0.9617
Absolute SS	817.4
Sy.x	5.309
Runs test	
Points above curve	4
Points below curve	8
Number of runs	4
P value (runs test)	0.1091
Deviation from Model	Not Significant
Number of points	
Analyzed	31

L. sericata 30.0° C.

L. sericata 30.0° C														
Dataset														
Time	egg		L1		L2		L3f		L3m		P		A	
	Mean	SE	Mean	SE	Mean	SE	Mean	SE	Mean	SE	Mean	SE	Mean	SE
0.0	100	0	0	0	0	0	0	0	0	0	0	0	0	0
4.1	100	0	0	0	0	0	0	0	0	0	0	0	0	0
8.1	86.5	15.6	13.5	15.6	0	0	0	0	0	0	0	0	0	0
12.1	29.7	20.3	70.3	20.3	0	0	0	0	0	0	0	0	0	0
15.3	37.1	15.5	62.9	15.5	0	0	0	0	0	0	0	0	0	0
20.2	54.4	13.8	45.6	13.8	0	0	0	0	0	0	0	0	0	0
27.2	0	0	0	0	100	0	0	0	0	0	0	0	0	0
34.2	0	0	0	0	100	0	0	0	0	0	0	0	0	0
41.3	0	0	0	0	49.6	43.6	50.4	43.6	0	0	0	0	0	0
48.2	0	0	0	0	15	22.3	85	22.3	0	0	0	0	0	0
55.2	0	0	0	0	0	0	100	0	0	0	0	0	0	0
65.1	0	0	0	0	0	0	100	0	0	0	0	0	0	0
75.2	0	0	0	0	0	0	97.4	3.6	2.6	3.6	0	0	0	0
85.1	0	0	0	0	0	0	77.3	39.4	22.7	39.4	0	0	0	0
95.1	0	0	0	0	0	0	0	0	100	0	0	0	0	0
101.1	0	0	0	0	0	0	3.3	5.8	96.7	5.8	0	0	0	0
123.0	0	0	0	0	0	0	0	0	86.5	15.4	13.5	15.4	0	0
141.1	0	0	0	0	0	0	0	0	46.7	31.3	53.3	31.3	0	0
159.3	0	0	0	0	0	0	1.7	2.9	4.5	7.9	93.8	7.4	0	0
177.0	0	0	0	0	0	0	0	0	3.2	3.2	96.8	3.2	0	0
195.0	0	0	0	0	0	0	0	0	1.7	2.9	98.3	2.9	0	0
237.0	0	0	0	0	0	0	0	0	0	0	100	0	0	0
279.0	0	0	0	0	0	0	0	0	0	0	88.7	12.1	11.3	12.1
322.0	0	0	0	0	0	0	0	0	0	0	6.3	10.8	93.8	10.8
363.0	0	0	0	0	0	0	0	0	0	0	18.4	7.8	81.6	7.8
405.0	0	0	0	0	0	0	0	0	0	0	0	0	100	0
405.0	0	0	0	0	0	0	0	0	0	0	0	0	100	0
405.0	0	0	0	0	0	0	0	0	0	0	0	0	100	0
405.0	0	0	0	0	0	0	0	0	0	0	0	0	100	0
405.0	0	0	0	0	0	0	0	0	0	0	0	0	100	0
405.0	0	0	0	0	0	0	0	0	0	0	0	0	100	0
Gaussian						Modified Gaussian					Cumulative Gaussian		Reversed Cumulative Gaussian	
	L1	L2	L3	L3m	P		L1	L2	L3	L3m		A		egg
							Ambiguous	Ambiguous						
Best-fit values						Best-fit values					Best-fit values		Best-fit values	
Amplitude	72.83	115.8	116.7	119	119.9	a	66.6	100	97.92	94.4	Mean	297.9	Mean	14.33
Mean	15.25	32.23	64.77	111.8	218.8	b	~ 14.42	~ 31.45	64.92	~ 113.4	SD	15.65	SD	9.125
SD	4.603	6.444	16.43	17.93	56.47	c	~ 5.882	~ 9.729	22.43	~ 27.40	Std. Error		Std. Error	
Std. Error						d	~ 16.22	~ 26.58	6.59	~ 31.94	Mean	2.477	Mean	1.038
Amplitude	3.116	5.373	6.105	7.008	6.776	Std. Error					SD	1.886	SD	1.629
Mean	0.2472	0.3465	0.9292	0.9427	3.453	a	1.007	2.887	0.9499	1.606	95% Confidence Intervals		95% Confidence Intervals	
SD	0.2527	0.3402	0.9256	1.093	3.32	b	~ 2388	~ 8.079e+006	0.1622	~ 501.7	Mean	292.9 to 303.0	Mean	12.20 to 16.45
95% Confidence Intervals						c	~ 1531	~ 9.121e+006	0.1795	~ 972.1	SD	11.80 to 19.51	SD	5.793 to 12.46
Amplitude	66.45 to 79.21	104.8 to 126.8	104.2 to 129.2	104.6 to 133.3	106.0 to 133.8	d	~ 144794	~ 2.829e+008	0.3966	~ 51806	Goodness of Fit		Goodness of Fit	
Mean	14.75 to 15.76	31.52 to 32.94	62.87 to 66.67	109.9 to 113.7	211.7 to 225.8	95% Confidence Intervals					df	29	df	29
SD	4.085 to 5.120	5.748 to 7.141	14.53 to 18.32	15.70 to 20.17	49.67 to 63.27	a	64.53 to 68.67	94.08 to 105.9	95.97 to 99.87	91.11 to 97.69	R square	0.9939	R square	0.9204
Goodness of Fit						b	(Very wide)	(Very wide)	64.59 to 65.25	(Very wide)	Absolute SS	338.5	Absolute SS	2176
df	28	28	28	28	28	c	(Very wide)	(Very wide)	22.07 to 22.80	(Very wide)	Sy.x	3.416	Sy.x	8.662
R square	0.9627	0.9566	0.9423	0.9465	0.9346	d	(Very wide)	(Very wide)	5.776 to 7.404	(Very wide)			Runs test	
Absolute SS	371.9	885.5	2115	1352	2530	Goodness of Fit					Number of points		Points above curve	4
Sy.x	3.645	5.624	8.691	6.949	9.505	df	27	27	27	27	Analyzed	31	Points below curve	10
Constraints						R square	0.9973	0.989	0.9979	0.9945			Number of runs	4
SD	SD > 0.0	SD > 0.0	SD > 0.0	SD > 0.0	SD > 0.0	Absolute SS	27.38	225	76.8	139.2			P value (runs test)	0.0679
						Sy.x	1.007	2.887	1.687	2.271			Deviation from Model	Not Significant
Number of points						Constraints								
Analyzed	31	31	31	31	31	c	c > 0.0	c > 0.0	c > 0.0	c > 0.0			Number of points	
						d	d > 1.000	d > 1.000	d > 1.000	d > 1.000			Analyzed	31
						Number of points								
						Analyzed	31	31	31	31				

L. sericata 32.5° C.

L. sericata 32.5° C

Time	egg Mean	egg SE	L1 Mean	L1 SE	L2 Mean	L2 SE	L3f Mean	L3f SE	L3m Mean	L3m SE	P Mean	P SE	A Mean	A SE
0	100	0	0	0	0	0	0	0	0	0	0	0	0	0
5.1	100	0	0	0	0	0	0	0	0	0	0	0	0	0
10.1	51.1	30.2	48.9	30.2	0	0	0	0	0	0	0	0	0	0
15.3	29.9	36	70.1	36	0	0	0	0	0	0	0	0	0	0
20.3	33.1	25.5	66.9	25.5	0	0	0	0	0	0	0	0	0	0
25.2	14.6	9.6	85.4	9.6	0	0	0	0	0	0	0	0	0	0
33.2	0	0	10.7	5.5	89.3	5.5	0	0	0	0	0	0	0	0
41.2	0	0	3.9	4.2	96.1	4.2	0	0	0	0	0	0	0	0
49.2	0	0	0	0	1.5	2.5	98.5	2.5	0	0	0	0	0	0
57.1	0	0	0	0	8.3	10.2	91.7	10.2	0	0	0	0	0	0
65.1	0	0	0	0	1.6	2.7	98.4	2.7	0	0	0	0	0	0
76.1	0	0	0	0	2.8	4.8	97.2	4.8	0	0	0	0	0	0
87.1	0	0	0	0	0	0	100	0	0	0	0	0	0	0
98.1	0	0	0	0	0	0	100	0	0	0	0	0	0	0
110.1	0	0	0	0	0	0	100	0	0	0	0	0	0	0
121	0	0	0	0	0	0	62.5	41.5	37.5	41.5	0	0	0	0
141.1	0	0	0	0	0	0	6.3	10.8	90.6	10.4	3.1	5.4	0	0
161.1	0	0	0	0	0	0	0	0	35	23.7	65	23.7	0	0
181	0	0	0	0	0	0	4.2	7.2	8.3	14.4	87.5	21.7	0	0
201	0	0	0	0	0	0	0	0	0	0	100	0	0	0
221	0	0	0	0	0	0	0	0	0	0	100	0	0	0
269.1	0	0	0	0	0	0	0	0	0	0	100	0	0	0
317	0	0	0	0	0	0	0	0	0	0	75	30.6	25	30.6
365	0	0	0	0	0	0	0	0	16.7	23.6	16.7	23.6	66.7	47.1
413	0	0	0	0	0	0	0	0	0	0	0	0	100	0
461	0	0	0	0	0	0	0	0	0	0	0	0	100	0
461	0	0	0	0	0	0	0	0	0	0	0	0	100	0
461	0	0	0	0	0	0	0	0	0	0	0	0	100	0
461	0	0	0	0	0	0	0	0	0	0	0	0	100	0
461	0	0	0	0	0	0	0	0	0	0	0	0	100	0
461	0	0	0	0	0	0	0	0	0	0	0	0	100	0

Gaussian

	L1	L2	L3	L3m	P
**Best-fit values**					
Amplitude	80.22	107.7	109.6	102.3	90.26
Mean	18.74	29.64	65.21	131.6	263.9
SD	5.087	4.25	21.25	28.73	70.33
**Std. Error**					
Amplitude	1.648	5.769	7.562	7.385	5.277
Mean	0.122	0.325	1.657	2.233	5.088
SD	0.1204	0.3562	1.684	2.263	4.604
**95% Confidence Intervals**					
Amplitude	76.84 to 83.59	95.85 to 119.5	94.10 to 125.1	87.14 to 117.4	79.46 to 101.1
Mean	18.49 to 18.99	28.98 to 30.31	61.82 to 68.61	127.0 to 136.2	253.5 to 274.3
SD	4.840 to 5.334	3.521 to 4.979	17.80 to 24.70	24.10 to 33.37	60.90 to 79.76
**Goodness of Fit**					
df	28	28	28	28	28
R square	0.9914	0.9526	0.8938	0.8888	0.9202
Absolute SS	99.29	630.7	4598	3059	2063
Sy.x	1.883	4.746	12.82	10.45	8.583
**Constraints**					
SD	SD > 0.0	SD > 0.0	SD > 0.0	SD > 0.0	SD > 0.0
Number of points					
Analyzed	31	31	31	31	31

Modified Gaussian

	L1	L2	L3	L3m	P
	Ambiguous	Ambiguous			
**Best-fit values**					
a	78.98	221.8	85.64	96.9	74.75
b	18.75	28.89	~ 65.08	131.6	265.7
c	5.271	1.457	~ 31.60	31.66	101.1
d	2.159	~ 1.000	~ 57.73	2.517	7.427
**Std. Error**					
a	1.87	657.4	3.952	9.432	3.322
b	0.1218	0.8872	~ 2.390e+007	2.236	5.671
c	0.1889	6.018	~ 2.873e+007	4.289	5.601
d	0.1582		~ 5.303e+008	0.7728	1.875
**95% Confidence Intervals**					
a	75.14 to 82.82	-1127 to 1571	77.53 to 93.75	77.54 to 116.2	67.93 to 81.57
b	18.50 to 19.00	27.07 to 30.71	(Very wide)	127.0 to 136.2	254.1 to 277.3
c	4.883 to 5.659	0.0 to 13.81	(Very wide)	22.86 to 40.46	89.65 to 112.6
d	1.834 to 2.484		(Very wide)	1.000 to 4.103	3.580 to 11.27
**Goodness of Fit**					
df	27	27	27	27	27
R square	0.9917	0.9512	0.9513	0.8905	0.955
Absolute SS	95.44	650	2108	3012	1164
Sy.x	1.88	4.907	8.836	10.56	6.567
**Constraints**					
c	c > 0.0	c > 0.0	c > 0.0	c > 0.0	c > 0.0
d	d > 1.000	d > 1.000	d > 1.000	d > 1.000	d > 1.000
Number of points					
Analyzed	31	31	31	31	31

Cumulative Gaussian

	A
**Best-fit values**	
Mean	350.2
SD	49.48
Std. Error	
Mean	5.126
SD	7.146
**95% Confidence Intervals**	
Mean	339.7 to 360.6
SD	34.86 to 64.09
**Goodness of Fit**	
df	29
R square	0.978
Absolute SS	905.4
Sy.x	5.587
Number of points	
Analyzed	31

Reversed Cumulative Gaussian

	egg
**Best-fit values**	
Mean	14.1
SD	5.046
Std. Error	
Mean	0.1485
SD	0.2359
**95% Confidence Intervals**	
Mean	13.80 to 14.40
SD	4.563 to 5.528
**Goodness of Fit**	
df	29
R square	0.9966
Absolute SS	98.4
Sy.x	1.842
**Runs test**	
Points above curve	5
Points below curve	6
Number of runs	4
P value (runs test)	0.1104
Deviation from Model	Not Significant
Number of points	
Analyzed	31

Supplementary Materials S3

Datasets and statistical analysis summary of *Lucilia sericata* nonlinear regressions for 50% stage transition by temperature. Regression equation:

y = 100/(1+10^((LogEC50-x)*HillSlope))

where x = time (log time) and y = percent in stage. Where "interrupted" is indicated on a regression, data were insufficient to fit a curve (typically this means we obtained too few points in measuring portions of the transition curve). Where "ambiguous" is indicated on a regression, the program could not reach a final solution by iterative (numerical) methods.. The analysis is from GraphPad Prism 6.0 which uses the Marquardt and Levenberg approach for non-linear regression.

L. sericata 10° C.

***L. sericata* 10.0° C**	L1	L2	L3f	L3m	P	A
log(agonist) vs. normalized response -- Variable slope				Interrupted		
Best-fit values						
LogEC50	2.19	2.501	2.655	2.835	3.14	3.58
HillSlope	2.414	4.076	5.869	214.8	6.86	9.414
EC50	155	317.3	451.5	683.8	1380	3798
Std. Error						
LogEC50	0.02321	0.01958	0.02237		0.02109	0.01581
HillSlope	0.3905	0.8776	1.748		2.587	3.207
95% Confidence Intervals						
LogEC50	2.126 to 2.255	2.454 to 2.549	2.605 to 2.705		3.096 to 3.184	3.547 to 3.612
HillSlope	1.330 to 3.498	1.928 to 6.223	1.976 to 9.763		1.424 to 12.30	2.796 to 16.03
EC50	133.7 to 179.8	284.2 to 354.3	402.6 to 506.4		1246 to 1528	3524 to 4095
Goodness of Fit						
Degrees of Freedom	4	6	10		18	24
R square	0.9634	0.9327	0.8665		0.6914	0.7114
Absolute Sum of Squares	140.3	244.8	1717		3656	3395
Sy.x	5.922	6.387	13.1		14.25	11.89
Runs test						
Points above curve	3	2	3		2	3
Points below curve	3	6	9		18	23
Number of runs	4	3	4		4	6
P value (runs test)	0.7	0.2857	0.2		0.2842	0.4077
Deviation from Model	Not Significant	Not Significant	Not Significant		Not Significant	Not Significant
Number of points analyzed	6	8	12	15	20	26
	LogEC50		HillSlope		EC50	
	Value	SE	Value	SE	Value	SE
L1	2.190	0.023	2.414	0.391	155.047	
L2	2.501	0.020	4.076	0.878	317.305	
L3f	2.655	0.022	5.869	1.748	451.532	
L3m	2.835		214.846		683.849	
P	3.140	0.021	6.860	2.587	1380.160	
A	3.580	0.016	9.414	3.207	3798.426	

**Dataset**						
* starred and italicized data points were excluded from final regression						
log(time)	L1	L2	L3f	L3m	P	A
1.545359	0	0	0	0	0	0
1.846131	15.5	0	0	0	0	0
2.022291	33.7	0	0	0	0	0
2.146916	35.3	0	0	0	0	0
2.243462	56.9	0	0	0	0	0
2.36398	76.9	23.1	0	0	0	0
2.458172		50	0	0	0	0
2.535384		50	0	0	0	0
2.601109			50	0	0	0
2.658011			61.9	0	0	0
2.727541			45	0	0	0
2.787475			100	0	0	0
2.840158				92.9	0	0
2.887106				100	0	0
2.929462				100	0	0
2.995196					0	0
3.052309					50	0
3.102777					33.3	0
3.147985					12.5	0
3.188928					100	0
3.274166						20
3.34539						0
3.460155						0
3.507861						50
3.573689						11.1
3.630733						100

L. sericata 12.5° C.

***L. sericata* 12.5° C**	L1	L2	L3f	L3m	P	A
log(agonist) vs. normalized response – Variable slope				Ambiguous		
Best-fit values						
LogEC50	1.51	2.134	2.363	~ 2.635	3.295	3.461
HillSlope	2.42	4.088	8.158	~ 182.5	6.14	35.54
EC50	32.35	136.1	230.5	~ 431.3	1971	2891
Std. Error						
LogEC50	0.1559	0.02671	0.01772	~ 2.490e-006	0.004932	0.002677
HillSlope	1.574	1.065	2.296	~ 2.273	0.4259	11.44
95% Confidence Intervals						
LogEC50	-0.4711 to 3.491	2.060 to 2.208	2.321 to 2.405	(Very wide)	3.284 to 3.305	3.456 to 3.467
HillSlope	-17.58 to 22.42	1.132 to 7.045	2.727 to 13.59	(Very wide)	5.241 to 7.039	11.92 to 59.16
EC50	0.3380 to 3096	114.7 to 161.4	209.3 to 253.9	(Very wide)	1924 to 2018	2854 to 2928
Goodness of Fit						
Degrees of Freedom	1	4	7	10	17	24
R square	0.933	0.9443	0.9527	1	0.9815	0.9701
Absolute Sum of Squares	269.5	338.8	646.4	7.423E-19	103.5	571.9
Sy.x	16.42	9.203	9.609	2.725E-10	2.467	4.881
Runs test						
Points above curve	1	2	1	2	3	6
Points below curve	2	4	8	9	16	20
Number of runs	3	3	3	2	4	4
P value (runs test)	1	0.4	1	0.0364	0.0815	0.0009
Deviation from Model	Not Significant	Not Significant	Not Significant	Significant	Not Significant	Significant
Number of points						
Analyzed	3	6	9	12	19	26
	LogEC50		HillSlope		EC50	
	Value	SE	Value	SE	Value	SE
L1	1.509841	0.1559078	2.419713	1.573807	32.34751	
L2	2.13381	0.02671435	4.088431	1.064884	136.0847	
L3f	2.362672	0.01772014	8.158384	2.296432	230.5008	
L3m	2.634765	2.49036E-06	182.5313	2.273092	431.2857	
P	3.294597	0.004932407	6.140238	0.4259485	1970.594	
A	3.461043	0.002676541	35.54379	11.444	2890.963	

**Dataset**						
* starred and italicized data points were excluded from final regression						
log(time)	L1	L2	L3f	L3m	P	A
1.042871	0	0	0	0	0	0
1.664916	77.9	0	0	0	0	0
1.908999	77.4	0	0	0	0	0
2.065113		37.2	0	0	0	0
2.179384		68.8	0	0	0	0
2.316259		72.7	25	0	0	0
2.42019			87.3	0	0	0
2.503932			72.5	0	0	0
2.574147			93.8	0	0	0
2.634565				47.9	0	0
2.707645				100	0	0
2.770115				100	0	0
2.824825					0	0
2.873381					0	0
2.917006					_4.1*_	0
2.984578					_14.0*_	0
3.042969					_14.2*_	0
3.094471					3.5	0
3.140508					10.3	0
3.182129					23.3	5.4
3.26861					34.2	1.4
3.340662					69.4	16.5
3.402433					_73.0*_	16.7
3.456518						39.7
3.504607						100
3.504607						100

L. sericata 15.0° C.

***L. sericata* 15.0° C**	L1	L2	L3f	L3m	P	A
log(agonist) vs. normalized response -- Variable slope		Interrupted				
Best-fit values						
LogEC50	1.713	2.138	2.253	2.497	2.877	3.233
HillSlope	9.842	122.8	17.59	78.1	5.488	17.9
EC50	51.66	137.6	179.1	313.8	754	1709
Std. Error						
LogEC50	0.001607		0.0008488	0.0004665	0.01293	0.008174
HillSlope	0.5343		0.4598	5.118	0.7987	4.254
95% Confidence Intervals						
LogEC50	1.709 to 1.718		2.251 to 2.255	2.496 to 2.498	2.850 to 2.905	3.216 to 3.249
HillSlope	8.359 to 11.32		16.55 to 18.63	67.05 to 89.16	3.803 to 7.173	9.150 to 26.64
EC50	51.13 to 52.19		178.3 to 179.9	313.1 to 314.5	708.1 to 802.9	1644 to 1776
Goodness of Fit						
Degrees of Freedom	4		9	13	17	26
R square	0.9997		0.9997	0.9987	0.9199	0.9322
Absolute Sum of Squares	3.48		5.88	25	197.2	1642
Sy.x	0.9327		0.8083	1.387	3.406	7.947
Runs test						
Points above curve	3		3	2	1	7
Points below curve	3		8	13	18	21
Number of runs	4		4	5	3	4
P value (runs test)	0.7		0.2364	1	1	0.0002
Deviation from Model	Not Significant		Not Significant	Not Significant	Not Significant	Significant
Number of points						
Analyzed	6	7	11	15	19	28
	LogEC50		HillSlope		EC50	
	Value	SE	Value	SE	Value	SE
L1	1.713144	0.001607007	9.841871	0.5342584	51.65881	
L2	2.138497		122.8396		137.5615	
L3f	2.253009	0.000848848	17.59011	0.4598216	179.0644	
L3m	2.496633	0.00046653	78.10342	5.118027	313.7855	
P	2.877389	0.0129326	5.487879	0.7987151	754.0303	
A	3.232617	0.008174426	17.89533	4.253516	1708.507	

**Dataset**						
* starred and italicized data points were excluded from final regression						
log(time)	L1	L2	L3f	L3m	P	A
1.236054	0	0	0	0	0	0
1.533867	0	0	0	0	0	0
1.709482	48.2	0	0	0	0	0
1.833864	93.2	0	0	0	0	0
1.98802	100	0	0	0	0	0
2.053974	100	0	0	0	0	0
2.149553		95.8	0	0	0	0
2.228101			27.1	0	0	0
2.294751			83.8	0	0	0
2.352303			100	0	0	0
2.421796			100	0	0	0
2.481622				6.3	0	0
2.502478				74.1	0	0
2.581053				100	0	0
2.647473				95	5	0
2.691148					3	0
2.751722					16.3	0
2.801432					38.5	0
2.847614					34.3	0
2.889302					*5.0**	0
2.974162					*15.1**	28.6
3.044899					*34.9**	26.6
3.106384					*56.2**	11.2
3.159266					*64.1**	5.3
3.206861						24.6
3.272534						84.4
3.329401						100
3.379864						100

L. sericata 17.5° C.

***L. sericata* 17.5° C**	L1	L2	L3f	L3m	P	A
log(agonist) vs. normalized response -- Variable slope						
Best-fit values						
LogEC50	1.659	1.932	2.135	2.375	2.514	2.881
HillSlope	3.5	30.37	28.04	18.39	13.65	15.91
EC50	45.63	85.43	136.6	237.2	326.4	760.8
Std. Error						
LogEC50	0.03999	0.00945	0.001194	0.0004714	0.006797	0.001571
HillSlope	1.169	8.3	4.781	0.3376	2.473	0.8792
95% Confidence Intervals						
LogEC50	1.532 to 1.786	1.908 to 1.955	2.133 to 2.138	2.374 to 2.376	2.499 to 2.528	2.878 to 2.884
HillSlope	-0.2189 to 7.219	10.06 to 50.68	17.22 to 38.85	17.66 to 19.12	8.433 to 18.87	14.09 to 17.72
EC50	34.04 to 61.16	81.00 to 90.10	135.7 to 137.4	236.7 to 237.8	315.8 to 337.3	755.1 to 766.5
Goodness of Fit						
Degrees of Freedom	3	6	9	13	17	24
R square	0.9333	0.9972	0.998	0.9997	0.9691	0.9981
Absolute Sum of Squares	386.3	38.36	32.9	4.36	660.4	60.32
Sy.x	11.35	2.528	1.912	0.5791	6.233	1.585
Runs test						
Points above curve	2	1	3	2	1	3
Points below curve	3	7	8	13	18	23
Number of runs	4	3	4	4	3	6
P value (runs test)	0.9	1	0.2364	0.3714	1	0.4077
Deviation from Model	Not Significant	Not Significant	Not Significant	Not Significant	Not Significant	Not Significant
Number of points						
Analyzed	5	8	11	15	19	26
	LogEC50		HillSlope		EC50	
	Value	SE	Value	SE	Value	SE
L1	1.65922	0.03998688	3.499854	1.168679	45.62676	
L2	1.931607	0.009449857	30.36921	8.300381	85.42932	
L3f	2.135411	0.001194284	28.03586	4.781146	136.5873	
L3m	2.375128	0.000471389	18.39026	0.3376454	237.2071	
P	2.513714	0.006797008	13.65121	2.473212	326.373	
A	2.881255	0.001571311	15.90591	0.879162	760.7725	

**Dataset**						
* starred and italicized data points were excluded from final regression						
log(time)	L1	L2	L3f	L3m	P	A
1.081887	0	0	0	0	0	0
1.383217	0	0	0	0	0	0
1.558859	44.2	0	0	0	0	0
1.684022	45.2	0	0	0	0	0
1.779476		0	0	0	0	0
1.898176	91.2	8.8	0	0	0	0
1.99189		98.6	0	0	0	0
2.068371		93.8	6.3	0	0	0
2.133832			46.9	0	0	0
2.190588			100	0	0	0
2.257878			100	0	0	0
2.316198				6.3	0	0
2.36748				42.9	0	0
2.413481				82.5	0	0
2.4552				97.2	2.8	0
2.522575					69.6	0
2.581034					73.6	0
2.632525					93.8	0
2.67859					89.3	0
2.720221						0
2.802805						0
2.872212						44.3
2.931958						83.3
2.98455						100
3.031408						96.4
3.103176						100

L. sericata 20.0° C.

***L. sericata* 20.0° C**	L1	L2	L3f	L3m	P	A
log(agonist) vs. normalized response -- Variable slope	Interrupted	Interrupted			Interrupted	Ambiguous
Best-fit values						
LogEC50	1.447	1.763	1.964	2.277	2.336	~ 2.743
HillSlope	33.46	98.41	36.98	75.89	215.3	~ 127.2
EC50	28.01	57.92	92.11	189.3	217	~ 553.4
Std. Error						
LogEC50			0.00001851	0.0207		~ 259.2
HillSlope			0.0264	120.9		~ 4.395e+006
95% Confidence Intervals						
LogEC50			1.964 to 1.964	2.232 to 2.322		(Very wide)
HillSlope			36.92 to 37.04	-185.3 to 337.1		(Very wide)
EC50			92.10 to 92.12	170.8 to 209.8		(Very wide)
Goodness of Fit						
Degrees of Freedom			9	13		23
R square			1	0.9886		0.9996
Absolute Sum of Squares			0.00115	156.2		13.69
Sy.x			0.01131	3.466		0.7715
Runs test						
Points above curve			3	1		2
Points below curve			8	14		22
Number of runs			4	3		3
P value (runs test)			0.2364	1		0.087
Deviation from Model			Not Significant	Not Significant		Not Significant
Number of points						
Analyzed	3	7	11	15	17	25
	LogEC50		HillSlope		EC50	
	Value	SE	Value	SE	Value	SE
L1	1.447243		33.45631		28.00549	
L2	1.762846		98.41061		57.92239	
L3f	1.964321	1.85109E-05	36.97882	0.02640347	92.11302	
L3m	2.277081	0.02070029	75.88952	120.9227	189.2694	
P	2.336397		215.3207		216.9686	
A	2.743006	259.2079	127.1961	4394835	553.3578	

**Dataset**						
* starred and italicized data points were excluded from final regression						
log(time)	L1	L2	L3f	L3m	P	A
0.956649	0	0	0	0	0	0
1.257278	0	0	0	0	0	0
1.435367	28.6	0	0	0	0	0
1.557607		0	0	0	0	0
1.655018		0	0	0	0	0
1.771434		87.5	0	0	0	0
1.864783		100	0	0	0	0
1.940122			11.3	0	0	0
2.004751			96.9	0	0	0
2.060934			100	0	0	0
2.130602			100	0	0	0
2.190495				0	0	0
2.243172				0	0	0
2.290035				90.6	0	0
2.332472				87.5	12.5	0
2.399703					100	0
2.457882					100	0
2.509225						0
2.555094						0
2.596597						0
2.680336						0
2.750508						90
2.810904						100
2.863917						96.3
2.911158						100

L. sericata 22.5 C.

L. sericata 22.5° C	L1	L2	L3f	L3m	P	A
log(agonist) vs. normalized response -- Variable slope		Interrupted				
Best-fit values						
LogEC50	1.345	1.66	1.889	2.15	2.306	2.628
HillSlope	13.31	104.8	30.03	12.03	26.05	77.39
EC50	22.13	45.74	77.51	141.1	202.4	424.6
Std. Error						
LogEC50	0.009957		0.001529	0.006895	0.0002226	0.2165
HillSlope	5.194		4.503	2.114	0.4109	884
95% Confidence Intervals						
LogEC50	1.313 to 1.377		1.886 to 1.893	2.135 to 2.165	2.306 to 2.307	2.180 to 3.076
HillSlope	-3.220 to 29.84		20.00 to 40.07	7.465 to 16.60	25.18 to 26.91	-1752 to 1906
EC50	20.57 to 23.81		76.91 to 78.12	136.4 to 146.1	202.2 to 202.6	151.3 to 1191
Goodness of Fit						
Degrees of Freedom	3		10	13	17	23
R square	0.9897		0.9991	0.9532	0.9999	0.9985
Absolute Sum of Squares	89.51		21.02	573	1.302	48.89
Sy.x	5.462		1.45	6.639	0.2768	1.458
Runs test						
Points above curve	2		3	1	3	2
Points below curve	3		9	14	16	23
Number of runs	5		4	3	4	4
P value (runs test)	1		0.2	1	0.0815	0.23
Deviation from Model	Not Significant		Not Significant	Not Significant	Not Significant	Not Significant
Number of points						
Analyzed	5	8	12	15	19	25
	LogEC50		HillSlope		EC50	
	Value	SE	Value	SE	Value	SE
L1	1.34501	0.009957033	13.30862	5.194256	22.13144	
L2	1.6603		104.7518		45.7404	
L3f	1.889368	0.001528542	30.03393	4.502685	77.51189	
L3m	2.149634	0.006894524	12.03136	2.113842	141.1347	
P	2.306204	0.000222566	26.04704	0.4108769	202.397	
A	2.62794	0.2165114	77.39233	884.0189	424.5608	

**Dataset**						
* starred and italicized data points were excluded from final regression						
log(time)	L1	L2	L3f	L3m	P	A
0.848446	0	0	0	0	0	0
1.151906	5	0	0	0	0	0
1.325652	35.4	0	0	0	0	0
1.450313	97.5	0	0	0	0	0
1.545256	91.7	0	0	0	0	0
1.664093		71.4	0	0	0	0
1.756953		100	0	0	0	0
1.832961		100	0	0	0	0
1.897902			64.7	0	0	0
1.954665			94.8	0	0	0
2.025562			100	0	0	0
2.086745			100	0	0	0
2.140167				59.4	0	0
2.188026				66.7	0	0
2.230609				85	0	0
2.296894					36.5	0
2.354229					94.4	0
2.404834					100	0
2.450275					100	0
2.491385						0
2.576341						0
2.646894						96.7
2.708421						98
2.761928						93.3
2.813581						100

L. sericata 25.0° C.

***L. sericata* 25.0° C**	L1	L2	L3f	L3m	P	A
log(agonist) vs. normalized response -- Variable slope	Interrupted					
Best-fit values						
LogEC50	1.228	1.585	1.794	2.047	2.225	2.568
HillSlope	11.19	102	20.2	60.17	20.13	25.89
EC50	16.92	38.44	62.3	111.3	167.8	370.2
Std. Error						
LogEC50	0.0144		0.0007994	0.003992	0.001102	0.001036
HillSlope	4.173		0.5171	22.69	1.094	3.598
95% Confidence Intervals						
LogEC50	1.183 to 1.274		1.793 to 1.796	2.038 to 2.055	2.222 to 2.227	2.566 to 2.571
HillSlope	-2.088 to 24.47		19.03 to 21.37	10.72 to 109.6	17.82 to 22.44	18.45 to 33.34
EC50	15.23 to 18.81		62.04 to 62.56	109.1 to 113.6	166.9 to 168.7	368.3 to 372.0
Goodness of Fit						
Degrees of Freedom	3		9	12	17	23
R square	0.9937		0.9997	0.9994	0.9988	0.9981
Absolute Sum of Squares	58.91		5.836	7.735	27.88	49.69
Sy.x	4.431		0.8053	0.8028	1.281	1.47
Runs test						
Points above curve	1		3	1	2	2
Points below curve	4		8	13	17	23
Number of runs	3		4	3	4	4
P value (runs test)	1		0.2364	1	0.2982	0.23
Deviation from Model	Not Significant		Not Significant	Not Significant	Not Significant	Not Significant
Number of points						
Analyzed	5	7	11	14	19	25
	LogEC50		HillSlope		EC50	
	Value	SE	Value	SE	Value	SE
L1	1.228471	0.01440128	11.18917	4.172692	16.92274	
L2	1.584817		102.0207		38.44302	
L3f	1.794457	0.00079943	20.20193	0.5170847	62.29551	
L3m	2.046651	0.003991658	60.16519	22.68968	111.3398	
P	2.224674	0.001101964	20.12869	1.09356	167.7542	
A	2.568397	0.001036366	25.8913	3.598356	370.1664	

**Dataset**						
* starred and italicized data points were excluded from final regression						
log(time)	L1	L2	L3f	L3m	P	A
0.785626	0	0	0	0	0	0
1.083682	0	0	0	0	0	0
1.26156	70.7	0	0	0	0	0
1.383666	94.8	0	0	0	0	0
1.479167	93.4	0	0	0	0	0
1.593101		87.5	0	0	0	0
1.683947		100	0	0	0	0
1.756826			15.2	0	0	0
1.820119			76.2	0	0	0
1.875592			100	0	0	0
1.944996			100	0	0	0
2.004518				0	0	0
2.057206				81.2	0	0
2.104259				97.2	0	0
2.146967					0	0
2.215571					40.1	0
2.274341					90.8	0
2.326583					94.6	0
2.372912					100	0
2.414973						0
2.499687						0
2.570543						53.4
2.631461						96.9
2.684845						93.1
2.732394						100

L. sericata 27.5° C.

***L. sericata* 27.5° C**	L1	L2	L3f	L3m	P	A
log(agonist) vs. normalized response – Variable slope						
Best-fit values						
LogEC50	1.073	1.489	1.667	2.094	2.197	2.537
HillSlope	2.373	29.23	74.36	22.77	23.07	14.99
EC50	11.83	30.87	46.5	124.3	157.6	344.5
Std. Error						
LogEC50	0.05188	0.0267	0.4354	0.001935	0.00195	0.001775
HillSlope	0.6716	24.71	1324	2.179	2.435	0.7553
95% Confidence Intervals						
LogEC50	0.9080 to 1.238	1.421 to 1.558	0.6635 to 2.671	2.090 to 2.099	2.193 to 2.202	2.533 to 2.541
HillSlope	0.2357 to 4.510	-34.31 to 92.77	-2980 to 3128	18.10 to 27.44	17.93 to 28.21	13.43 to 16.56
EC50	8.092 to 17.31	26.35 to 36.15	4.608 to 469.3	123.1 to 125.5	156.1 to 159.1	341.6 to 347.4
Goodness of Fit						
Degrees of Freedom	3	5	8	14	17	23
R square	0.9174	0.9988	0.9963	0.9932	0.9947	0.9969
Absolute Sum of Squares	359.6	15.11	71.45	58.13	97.76	66.53
Sy.x	10.95	1.738	2.989	2.038	2.398	1.701
Runs test						
Points above curve	2	1	1	1	2	3
Points below curve	3	6	9	15	17	22
Number of runs	3	3	3	3	4	4
P value (runs test)	0.5	1	1	1	0.2982	0.0474
Deviation from Model	Not Significant	Not Significant	Not Significant	Not Significant	Not Significant	Significant
Number of points						
Analyzed	5	7	10	16	19	25
	LogEC50		HillSlope		EC50	
	Value	SE	Value	SE	Value	SE
L1	1.073115	0.05187581	2.372677	0.6715761	11.83355	
L2	1.489477	0.0266954	29.23228	24.71381	30.86573	
L3f	1.667488	0.4353856	74.35593	1324.423	46.50373	
L3m	2.094481	0.001934841	22.77118	2.178688	124.3028	
P	2.197497	0.001950332	23.07309	2.435186	157.5786	
A	2.537146	0.001775475	14.99456	0.7553464	344.4659	

**Dataset**						
* starred and italicized data points were excluded from final regression						
log(time)	L1	L2	L3f	L3m	P	A
0.704722	0	0	0	0	0	0
1.005931	48.9	0	0	0	0	0
1.18327	70.1	0	0	0	0	0
1.306514	66.9	0	0	0	0	0
1.400754	85.4	0	0	0	0	0
1.520975		89.3	0	0	0	0
1.615117		96.1	0	0	0	0
1.691928			98.5	0	0	0
1.756699			91.7	0	0	0
1.813581			98.4	0	0	0
1.881504				0	0	0
1.93981				0	0	0
1.991595				0	0	0
2.041787				0	0	0
2.082785				37.5	0	0
2.149591				90.6	3.1	0
2.207051					65	0
2.257679					87.5	0
2.303196					100	0
2.344392						0
2.42984						0
2.501059						25
2.562293						66.7
2.61595						100
2.663701						100

L. sericata 30.0° C.

L. sericata **30.0° C**	L1	L2	L3f	L3m	P	A
log(agonist) vs. normalized response -- Variable slope		Ambiguous				
Best-fit values						
LogEC50	1.058	~ 1.391	1.619	1.938	2.144	2.473
HillSlope	3.885	~ 360.1	14.4	67.55	16.63	32.25
EC50	11.44	~ 24.60	41.56	86.67	139.3	297.2
Std. Error						
LogEC50	0.04688	~	0.002951	0.006475	0.001078	0.004462
HillSlope	1.806	~	1.466	55.44	0.644	4.831
95% Confidence Intervals						
LogEC50	0.8566 to 1.260	(Very wide)	1.612 to 1.625	1.924 to 1.952	2.142 to 2.146	2.464 to 2.482
HillSlope	-3.887 to 11.66	(Very wide)	11.09 to 17.72	-52.21 to 187.3	15.28 to 17.98	22.25 to 42.25
EC50	7.188 to 18.20	(Very wide)	40.93 to 42.21	83.93 to 89.51	138.5 to 140.0	290.9 to 303.6
Goodness of Fit						
Degrees of Freedom	2	5	9	13	19	23
R square	0.8853	1	0.9967	0.9989	0.9992	0.985
Absolute Sum of Squares	424.3	0	63.53	17.63	25.17	333.5
Sy.x	14.57	5.942E-30	2.657	1.165	1.151	3.808
Runs test						
Points above curve	1	1	3	2	3	2
Points below curve	3	5	8	13	18	23
Number of runs	3	2	4	5	6	4
P value (runs test)	1	0.3333	0.2364	1	0.4887	0.23
Deviation from Model	Not Significant	Not Significant	Not Significant	Not Significant	Not Significant	Not Significant
Number of points						
Analyzed	4	7	11	15	21	25
	LogEC50		HillSlope		EC50	
	Value	SE	Value	SE	Value	SE
L1	1.058351	0.04687951	3.884845	1.806252	11.43803	
L2	1.390995		360.1489		24.60338	
L3f	1.61869	0.002950681	14.40494	1.466204	41.56142	
L3m	1.937892	0.006474931	67.54924	55.44376	86.67464	
P	2.143842	0.00107768	16.62763	0.6440066	139.2652	
A	2.473039	0.00446208	32.24966	4.831073	297.1935	

**Dataset**						
* starred and italicized data points were excluded from final regression						
log(time)	L1	L2	L3f	L3m	P	A
0.613225	0	0	0	0	0	0
0.909601	13.5	0	0	0	0	0
1.084427	70.3	0	0	0	0	0
1.183507	62.9	0	0	0	0	0
1.305262		0	0	0	0	0
1.43397		100	0	0	0	0
1.534079		100	0	0	0	0
1.615775			50.4	0	0	0
1.68331			85	0	0	0
1.741644			100	0	0	0
1.813803			100	0	0	0
1.87629				2.6	0	0
1.930015				22.7	0	0
1.978181				100	0	0
2.004608				96.7	0	0
2.089905					13.5	0
2.149412					53.3	0
2.202159					93.8	0
2.247973					96.8	0
2.290035					98.3	0
2.374809					100	0
2.445604						11.3
2.507856						93.8
2.559907						81.6
2.607455						100

L. sericata 32.5° C.

L. sericata **32.5° C**	L1	L2	L3f	L3m	P	A	**Dataset**						
log(agonist) vs. normalized response -- Variable slope	Interrupted						* starred and italicized data points were excluded from final regression						
Best-fit values							log(time)	L1	L2	L3f	L3m	P	A
LogEC50	1.139	1.4	1.619	1.978	2.252	2.543	0.611458	0	0	0	0	0	0
HillSlope	4.308	110.3	14.19	10.99	6.571	11.48	0.910491	5.8	0	0	0	0	0
EC50	13.77	25.11	41.59	95.09	178.6	349.1	1.094442	43.3	0	0	0	0	0
Std. Error							1.182795	57.7	0	0	0	0	0
LogEC50	0.009731		0.003305	0.01157	0.008096	0.007449	1.285932	81.3	0	0	0	0	0
HillSlope	0.4771		1.079	3.094	0.7149	2.115	1.402046		63.7	0	0	0	0
95% Confidence Intervals							1.494039		100	0	0	0	0
LogEC50	1.108 to 1.170		1.611 to 1.627	1.953 to 2.003	2.235 to 2.269	2.528 to 2.558	1.592732			90.0*	0	0	0
HillSlope	2.789 to 5.826		11.71 to 16.68	4.350 to 17.62	5.084 to 8.058	7.119 to 15.85	1.655178			77.2	0	0	0
EC50	12.82 to 14.79		40.86 to 42.32	89.81 to 100.7	171.8 to 185.6	337.0 to 361.7	1.708633			93.1	0	0	0
Goodness of Fit							1.779356			100	0	0	0
Degrees of Freedom	3		8	14	21	24	1.840603				0	0	0
R square	0.9917		0.9996	0.8745	0.9719	0.9137	1.892813				0	0	0
Absolute Sum of Squares	39.59		6.807	1458	575.4	996.9	1.940641				52.9	0	0
Sy.x	3.633		0.9224	10.2	5.235	6.445	1.983043				30.3	0	0
Runs test							2.049735				100	0	0
Points above curve	2		2	2	3	2	2.092165					0	0
Points below curve	3		8	14	20	24	2.158928					11.1	0
Number of runs	4		4	4	4	4	2.20438					36.2	0
P value (runs test)	0.9		0.5333	0.35	0.0559	0.2215	2.245831					63.1	0
Deviation from Model	Not Significant		Not Significant	Not Significant	Not Significant	Not Significant	2.328677					66	0
							2.398099					82.8	0
Number of points							2.439484					96.7	0
Analyzed	5	7	10	16	23	26	2.495035						41.7
	LogEC50		HillSlope		EC50		2.542825						30.9
	Value	SE	Value	SE	Value	SE	2.61066						100
L1	1.139	0.009731171	4.307749	0.4771413	13.77211								
L2	1.399831		110.2617		25.10909								
L3f	1.618942	0.003305214	14.19396	1.079273	41.58552								
L3m	1.978156	0.01156718	10.98604	3.093692	95.09459								
P	2.251843	0.008095888	6.571046	0.7148767	178.5843								
A	2.542994	0.007449316	11.48417	2.114877	349.1358								

Supplementary Materials S4

Development data for *Phormia regina* (from Excel).

Transitions at 50% by Dose Analysis							
Temp		Hours to Stage Transition					
Mean	SE	E-L1	E-L2	E-L3f	E-L3m	E-P	E-A
10.2	0.16						
12.5	0.13	70.2	354.3	564.3	612.5	843.8	1451.0
15.0	0.15	61.4	154.1	230.7	445.9	468.6	965.9
17.5	0.09	40.0	126.9	217.6	277.4	318.6	622.5
20.0	0.1	24.3	74.6	122.7	202.4	244.8	447.2
22.4	0.90	15.5	49.8	91.6	134.0	178.3	356.1
25.0	0.08	15.2	43.3	71.3	123.2	154.8	283.4
27.5	0.07	13.8	33.9	56.6	95.3	122.1	239.2
30.0	0.23	10.3	27.3	46.6	75.6	106.7	217.8
32.5	0.07	10.1	25.1	42.0	78.2	105.9	205.6

Temp		Hours in Stage					
Mean	SE	Egg	L1	L2	L3f	L3m	P
10.2	0.16						
12.5	0.13	70.2	284.1	210.0	48.2	231.4	607.1
15.0	0.15	61.4	92.7	76.6	215.2	22.7	497.3
17.5	0.09	40.0	86.9	90.7	59.8	41.2	303.9
20.0	0.1	24.3	50.4	48.1	79.7	42.4	202.4
22.4	0.90	15.5	34.3	41.8	42.4	44.3	177.8
25.0	0.08	15.2	28.2	28.0	51.9	31.6	128.6
27.5	0.07	13.8	20.0	22.7	38.7	26.7	117.2
30.0	0.23	10.3	17.1	19.3	28.9	31.1	111.1
32.5	0.07	10.1	15.1	16.9	36.2	27.7	99.7

Temp		Days to Stage Transition					
Mean	SE	E-L1	E-L2	E-L3f	E-L3m	E-P	E-A
10.2	0.16	0.0					
12.5	0.13	2.9	14.8	23.5	25.5	35.2	60.5
15.0	0.15	2.6	6.4	9.6	18.6	19.5	40.2
17.5	0.09	1.7	5.3	9.1	11.6	13.3	25.9
20.0	0.1	1.0	3.1	5.1	8.4	10.2	18.6
22.4	0.90	0.6	2.1	3.8	5.6	7.4	14.8
25.0	0.08	0.6	1.8	3.0	5.1	6.4	11.8
27.5	0.07	0.6	1.4	2.4	4.0	5.1	10.0
30.0	0.23	0.4	1.1	1.9	3.1	4.4	9.1
32.5	0.07	0.4	1.0	1.8	3.3	4.4	8.6

Temp		Days in Stage					
Mean	SE	Egg	L1	L2	L3f	L3m	P
10.2	0.16	0.0					
12.5	0.13	2.9	11.8	8.7	2.0	9.6	25.3
15.0	0.15	2.6	3.9	3.2	9.0	0.9	20.7
17.5	0.09	1.7	3.6	3.8	2.5	1.7	12.7
20.0	0.1	1.0	2.1	2.0	3.3	1.8	8.4
22.4	0.90	0.6	1.4	1.7	1.8	1.8	7.4
25.0	0.08	0.6	1.2	1.2	2.2	1.3	5.4
27.5	0.07	0.6	0.8	0.9	1.6	1.1	4.9
30.0	0.23	0.4	0.7	0.8	1.2	1.3	4.6
32.5	0.07	0.4	0.6	0.7	1.5	1.2	4.2

Temp		1/Days to Stage Transition					
Mean	SE	E-L1	E-L2	E-L3f	E-L3m	E-P	E-A
10.2	0.51						
12.5	0.18	0.342	0.068	0.043	0.039	0.028	0.017
15.0	0.14	0.391	0.156	0.104	0.054	0.051	0.025
17.5	0.12	0.600	0.189	0.110	0.087	0.075	0.039
20.0	0.09	0.989	0.322	0.196	0.119	0.098	0.054
22.4	0.10	1.548	0.482	0.262	0.179	0.135	0.067
25.0	0.06	1.584	0.554	0.337	0.195	0.155	0.085
27.5	0.06	1.738	0.709	0.424	0.252	0.197	0.100
30.0	0.05	2.336	0.878	0.515	0.318	0.225	0.110
32.5	0.02	2.387	0.956	0.571	0.307	0.227	0.117

Temp		1/Days in Stage					
Mean	SE	Egg	L1	L2	L3f	L3m	P
10.2	0.51						
12.5	0.18	0.342	0.084	0.114	0.498	0.104	0.040
15.0	0.14	0.391	0.259	0.313	0.112	1.058	0.048
17.5	0.12	0.600	0.276	0.265	0.401	0.583	0.079
20.0	0.09	0.989	0.477	0.499	0.301	0.566	0.119
22.4	0.10	1.548	0.699	0.574	0.566	0.542	0.135
25.0	0.06	1.584	0.852	0.858	0.462	0.760	0.187
27.5	0.06	1.738	1.198	1.055	0.620	0.898	0.205
30.0	0.05	2.336	1.406	1.245	0.829	0.771	0.216
32.5	0.02	2.387	1.594	1.420	0.663	0.866	0.241

	Linear Regression Results (from Graph Pad Prism)					
	1/Days to Stage Transition					
	E-L1	E-L2	E-L3f	E-L3m	E-P	E-A
Dev Min (x-intercept):	11.5	12.9	12.8	11.9	10.5	10.2
ADD (1/slope):	8.4	20.4	34.7	62.9	91.4	181.9
Range min:	15.0	15.0	15.0	15.0	15.0	15.0
Range max:	32.5	32.5	32.5	32.5	32.5	32.5

	1/Days in Stage					
	Egg	L1	L2	L3f	L3m	P
Dev Min (x-intercept):	11.5	13.5	12.5	8.2	2.3	10.5
ADD (1/slope):	8.4	12.1	14.4	29.3	30.2	84.9
Range min:	15.0	15.0	15.0	17.5	17.5	15.0
Range max:	32.5	32.5	32.5	30.0	27.5	32.5

Comparison of Transition vs. Stage Results				
	trans	by stage	diff (t-s)	% diff (d/t)
**Egg-L1**	8.4	11.5	-3.1	-36.8%
**L1-L2**	20.4	25.0	-4.6	-22.4%
**L2-L3f**	34.7	39.4	-4.7	-13.5%
**L3f-L3m**	62.9	68.7	-5.8	-9.3%
**L3m-P**	91.4	98.9	-7.5	-8.2%
**P-A**	181.9	183.8	-1.9	-1.1%

From Prism (Linear Regressions): missing values represent non-linearity							
Temp		Transition ADD by 1/Day					
Mean	SE	E-L1	E-L2	E-L3f	E-L3m	E-P	E-A
10.2	0.51						
12.5	0.18						
15.0	0.14	8.9	13.7	21.3	58.8	89.4	195.9
17.5	0.12	10.0	24.6	42.8	65.6	94.0	191.2
20.0	0.09	8.6	22.2	37.0	69.0	97.8	184.1
22.4	0.10	7.0	19.7	36.6	58.8	88.7	181.5
25.0	0.06	8.5	21.9	36.2	67.5	93.9	175.3
27.5	0.06	9.2	20.6	34.6	62.1	86.7	172.8
30.0	0.05	7.9	19.5	33.4	57.1	86.8	179.9
32.5	0.02	8.8	20.5	34.5	67.3	97.4	191.5

Temp		Stage ADD by 1/Days					
Mean	SE	Egg	L1	L2	L3f	L3m	P
10.2	0.51						
12.5	0.18						
15.0	0.14	8.9	6.0	7.9	61.3	12.0	94.0
17.5	0.12	10.0	14.7	18.9	23.3	26.2	89.2
20.0	0.09	8.6	13.8	15.0	39.3	31.4	80.5
22.4	0.10	7.0	12.7	17.2	25.1	37.1	88.2
25.0	0.06	8.5	13.5	14.5	36.4	29.9	77.8
27.5	0.06	9.2	11.7	14.2	31.2	28.1	83.0
30.0	0.05	7.9	11.7	14.0	26.3	35.9	90.2
32.5	0.02	8.8	11.9	14.1	36.7	34.9	91.5

Temp (mean)	Transition ADD by 1/Day					
	E-L1	E-L2	E-L3f	E-L3m	E-P	E-A
10.2						
12.5						
15.0	8.9	13.7	21.3	58.8		
17.5	10.0	24.6	42.8	65.6	94.0	191.2
20.0	8.6	22.2	37.0	69.0	97.8	184.1
22.4	7.0	19.7	36.6	58.8	88.7	181.5
25.0	8.5	21.9	36.2	67.5	93.9	175.3
27.5	9.2	20.6	34.6	62.1	86.7	172.8
30.0	7.9	19.5	33.4	57.1	86.8	179.9
32.5	8.8	20.5	34.5	67.3		
**mean**	8.6	20.3	34.6	63.3	91.3	180.8
**SE**	0.8	2.9	5.7	4.4	4.2	6.0
**n**	8	8	8	8	6	6
**Regression ADD**	8	20	35	63	91	182
**% deviation**	2.1%	-0.5%	-0.4%	0.6%	-0.1%	-0.6%
**ADD Range min:**	15.0	15.0	15.0	15.0	15.0	15.0
**ADD Range max:**	32.5	32.5	32.5	32.5	32.5	32.5

Temp (mean)	Stage ADD by 1/Days					
	Egg	L1	L2	L3f	L3m	P
10.2						
12.5						
15.0	8.9	6.0	7.9			
17.5	10.0	14.7	18.9	23.3	26.2	89.2
20.0	8.6	13.8	15.0	39.3	31.4	80.5
22.4	7.0	12.7	17.2	25.1	37.1	88.2
25.0	8.5	13.5	14.5	36.4	29.9	77.8
27.5	9.2	11.7	14.2	31.2	28.1	83.0
30.0	7.9	11.7	14.0	26.3	35.9	90.2
32.5	8.8	11.9	14.1			91.5
**mean**	8.6	12.0	14.5	30.2	31.4	85.8
**SE**	0.8	2.5	3.0	5.9	4.0	4.9
**n**	8	8	8	6	6	7
**Regression ADD**	8	12	14	29	30	85
**% deviation**	2.1%	-1.0%	0.7%	3.1%	4.2%	1.0%
**ADD Range min:**	15.0	15.0	15.0	17.5	20.0	15.0
**ADD Range max:**	32.5	32.5	32.5	32.5	32.5	32.5

Temp	% Time in Stage					
	Egg	L1	L2	L3f	L3m	P
10.2						
12.5	5.1%	20.6%	15.2%	3.5%	16.8%	44.0%
15.0	6.8%	10.2%	8.5%	23.8%	2.5%	55.0%
17.5	6.9%	14.9%	15.6%	10.3%	7.1%	52.2%
20.0	5.7%	11.9%	11.4%	18.8%	10.0%	47.9%
22.4	4.6%	10.1%	12.3%	12.4%	13.0%	52.2%
25.0	5.6%	10.5%	10.4%	19.4%	11.8%	48.0%
27.5	6.1%	8.9%	10.1%	17.2%	11.9%	52.0%
30.0	5.0%	8.2%	9.3%	13.9%	15.0%	53.5%
32.5	5.1%	7.7%	8.6%	18.5%	14.2%	51.0%
**Mean**	5.65%	11.45%	11.26%	15.31%	11.35%	50.62%
**SE**	0.77%	3.81%	2.49%	5.67%	4.11%	3.22%
**CV**	13.55%	33.30%	22.11%	37.05%	36.20%	6.36%

Temp		% Total Development					
Mean	SE	E-L1	E-L2	E-L3f	E-L3m	E-P	E-A
10.2	0.51						
12.5	0.18	1.80%	9.09%	14.48%	15.72%	21.66%	37.24%
15.0	0.14	2.64%	6.62%	9.92%	19.17%	20.14%	41.52%
17.5	0.12	2.50%	7.92%	13.58%	17.31%	19.87%	38.83%
20.0	0.09	2.17%	6.69%	11.00%	18.14%	21.94%	40.07%
22.4	0.10	1.88%	6.04%	11.10%	16.24%	21.60%	43.15%
25.0	0.06	2.19%	6.27%	10.32%	17.83%	22.39%	41.00%
27.5	0.06	2.46%	6.04%	10.09%	17.00%	21.76%	42.65%
30.0	0.05	2.12%	5.65%	9.63%	15.60%	22.03%	44.97%
32.5	0.02	2.15%	5.38%	9.00%	16.75%	22.69%	44.04%
	**Mean**	2.21%	6.63%	11.01%	17.08%	21.57%	41.50%
	**SE**	0.26%	1.11%	1.74%	1.10%	0.90%	2.36%
	**CV**	11.85%	16.75%	15.77%	6.45%	4.16%	5.68%

Temp		ADD Temp * Days to Stage Transition					
Mean	SE	E-L1	E-L2	E-L3f	E-L3m	E-P	E-A
10.2	0.51	0.0					
12.5	0.18	29.7	2.7	4.3	4.7	6.5	11.1
15.0	0.14	26.0	0.9	1.3	2.5	2.7	5.5
17.5	0.12	16.9	0.6	1.1	1.4	1.6	3.1
20.0	0.09	10.3	0.3	0.5	0.8	0.9	1.7
22.4	0.10	6.6	0.2	0.4	0.5	0.7	1.4
25.0	0.06	6.4	0.1	0.2	0.3	0.4	0.8
27.5	0.06	5.8	0.1	0.1	0.2	0.3	0.6
30.0	0.05	4.4	0.1	0.1	0.2	0.2	0.5
32.5	0.02	4.3	0.0	0.0	0.1	0.1	0.1

Temp		ADD Temp * Days in Stage					
Mean	SE	Egg	L1	L2	L3f	L3m	P
10.2	0.51						
12.5	0.18	0.5	2.2	1.6	0.4	1.8	4.6
15.0	0.14	0.3	0.5	0.4	1.2	0.1	2.8
17.5	0.12	0.2	0.4	0.5	0.3	0.2	1.5
20.0	0.09	0.1	0.2	0.2	0.3	0.2	0.8
22.4	0.10	0.1	0.1	0.2	0.2	0.2	0.7
25.0	0.06	0.0	0.1	0.1	0.1	0.1	0.3
27.5	0.06	0.0	0.0	0.1	0.1	0.1	0.3
30.0	0.05	0.0	0.0	0.0	0.1	0.1	0.3
32.5	0.02	0.0	0.0	0.0	0.0	0.0	0.1

For Deming Regressions

Temp		Temp * Days to Stage Transition					
Mean	SE	E-L1	E-L2	E-L3f	E-L3m	E-P	E-A
10.2	0.51	0.0	0.0	0.0	0.0	0.0	0.0
12.5	0.18	0.5	2.7	4.3	4.7	6.5	11.1
15.0	0.14	0.3	0.9	1.3	2.5	2.7	5.5
17.5	0.12	0.2	0.6	1.1	1.4	1.6	3.1
20.0	0.09	0.1	0.3	0.5	0.8	0.9	1.7
22.4	0.10	0.1	0.2	0.4	0.5	0.7	1.4
25.0	0.06	0.0	0.1	0.2	0.3	0.4	0.8
27.5	0.06	0.0	0.1	0.1	0.2	0.3	0.6
30.0	0.05	0.0	0.1	0.1	0.2	0.2	0.5
32.5	0.02	0.0	0.0	0.0	0.1	0.1	0.1

Temp		Temp * Days in Stage					
Mean	SE	Egg	L1	L2	L3f	L3m	P
10.2	0.51	0.0	0.0	0.0	0.0	0.0	0.0
12.5	0.18	0.5	2.2	1.6	0.4	1.8	4.6
15.0	0.14	0.3	0.5	0.4	1.2	0.1	2.8
17.5	0.12	0.2	0.4	0.5	0.3	0.2	1.5
20.0	0.09	0.1	0.2	0.2	0.3	0.2	0.8
22.4	0.10	0.1	0.1	0.2	0.2	0.2	0.7
25.0	0.06	0.0	0.1	0.1	0.1	0.1	0.3
27.5	0.06	0.0	0.0	0.1	0.1	0.1	0.3
30.0	0.05	0.0	0.0	0.0	0.1	0.1	0.3
32.5	0.02	0.0	0.0	0.0	0.0	0.0	0.1

Data for Prism polynomial fit for egg development

Temp	Egg
10.2	0.155
12.5	0.742
15.0	0.465
17.5	0.526
20.0	0.857
22.4	1.084
25.0	1.418
27.5	2.028
30.0	2.098
32.5	1.743

Supplementary Materials S5

Datasets and statistical analysis summary of *Phormia regina* nonlinear regressions for percent in stage by temperature. Regression equations are :

Gaussian equation (a standard normal curve):

$$y = a \exp\left[-\frac{1}{2}\left(\frac{x-b}{c}\right)^2\right]$$

Modified Gaussian equation (a form of Gaussian curve with a plateau at 100%):

$$y = a \exp\left[-\frac{1}{2}\left(\frac{|x-b|}{c}\right)^d\right]$$

Cumulative Gaussian equation (a form of the Gaussian curve used for adults, to model a sigmoidal increase to a plateau)

$$y = \frac{a}{2}\left[1 + erf\left(\frac{x-b}{\sqrt{2}c}\right)\right]$$

Reversed cumulative Gaussian equation (a form of the cumulative Gaussian equation used for eggs, to model a sigmoidal decrease from a plateau)

$$y = \frac{a}{2}\left[1 - erf\left(\frac{x-b}{\sqrt{2}c}\right)\right]$$

where x = development time (hours) and y = percent in stage. Where "interrupted" is indicated on a regression, data were insufficient to fit a curve (typically this means we obtained too few points in measuring portions of the transition curve). Where "ambiguous" is indicated on a regression, the program could not reach a final solution by iterative (numerical) methods. The analysis is from GraphPad Prism 6.0 which uses the Marquardt and Levenberg approach for non-linear regression.

P. regina 10.0° C.

P. regina 10.0° C														
Dataset														
Time	egg		L1		L2		L3f		L3m		P		A	
	Mean	SE	Mean	SE	Mean	SE	Mean	SE	Mean	SE	Mean	SE	Mean	SE
0.0	100	0	0	0	0	0	0	0	0	0	0	0	0	0
17.5	100	0	0	0	0	0	0	0	0	0	0	0	0	0
32.1	100	0	0	0	0	0	0	0	0	0	0	0	0	0
48.2	100	0	0	0	0	0	0	0	0	0	0	0	0	0
64.1	100	0	0	0	0	0	0	0	0	0	0	0	0	0
80.1	100	0	0	0	0	0	0	0	0	0	0	0	0	0
124.3	72.8	35.1	27.2	35.1	0	0	0	0	0	0	0	0	0	0
168.1	36.7	32.6	63.3	32.6	0	0	0	0	0	0	0	0	0	0
212.1	35	40.9	65	40.9	0	0	0	0	0	0	0	0	0	0
256.1	47.5	47.6	52.5	47.6	0	0	0	0	0	0	0	0	0	0
292.0	16.7	23.6	83.3	23.6	0	0	0	0	0	0	0	0	0	0
363.1	0	0	100	0	0	0	0	0	0	0	0	0	0	0
426.1	0	0	100	0	0	0	0	0	0	0	0	0	0	0

Gaussian						
		L1	L2	L3f	L3m	P
			Ambiguous	Ambiguous	Ambiguous	Ambiguous
Best-fit values						
Amplitude		101.4	0	0	0	0
Mean		381.9	~ 152.0	~ 0.0	~ 0.0	~ 0.0
SD		149.8	~ 42.57	~ 42.61	~ 42.61	~ 42.61
Std. Error						
Amplitude		8.271				
Mean		33.11				
SD		25.22				
95% Confidence Intervals						
Amplitude		82.99 to 119.8				
Mean		308.1 to 455.7				
SD		93.65 to 206.0				
Goodness of Fit						
df		10	10	10	10	10
R square		0.9165	1	1	1	1
Absolute SS		1678	0	0	0	0
Sy.x		12.96				
Constraints						
SD		SD > 0.0	SD > 0.0	SD > 0.0	SD > 0.0	SD > 0.0
Number of points						
Analyzed		13	13	13	13	13

Modified Gaussian					
	L1	L2	L3	L3m	P
	Not converged	Ambiguous	Ambiguous	Ambiguous	Ambiguous
Best-fit values					
a		0	0	0	0
b		~ 0.0	~ 0.0	~ 0.0	~ 0.0
c		~ 1.000	~ 1.000	~ 1.000	~ 1.000
d		~ 1.000	~ 1.000	~ 1.000	~ 1.000
Std. Error					
a					
b					
c					
d					
95% Confidence Intervals					
a					
b					
c					
d					
Goodness of Fit					
df		9	9	9	9
R square		1	1	1	1
Absolute SS		0	0	0	0
Sy.x					
Constraints					
c	c > 0.0	c > 0.0	c > 0.0	c > 0.0	c > 0.0
d	d > 1.000	d > 1.000	d > 1.000	d > 1.000	d > 1.000
Number of points					
Analyzed	13	13	13	13	13

Cumulative Gaussian	
	A
	Ambiguous
Best-fit values	
Mean	~ 631947
SD	~ 22871
Std. Error	
Mean	
SD	
95% Confidence Intervals	
Mean	
SD	
Goodness of Fit	
Degrees of Freedom	11
R square	1
Absolute Sum of Squ	0
Sy.x	
Number of points	
Analyzed	13

Reversed Cumulative Gaussian	
	egg
Best-fit values	
	192.2
	94.74
	13.46
	17.55
95% Confidence Intervals	
	162.5 to 221.8
	56.11 to 133.4
Goodness of Fit	
	11
	0.9293
	1421
	11.36
Runs	
	8
	5
Number of runs	4
P value (runs test)	0.0536
Deviation from Model	Not Significant
Number of points	
Analyzed	13

P. *regina* 12.5° C.

P. regina 12.5° C														
Dataset														
Time	egg		L1		L2		L3f		L3m		P		A	
	Mean	SE	Mean	SE	Mean	SE	Mean	SE	Mean	SE	Mean	SE	Mean	SE
0.0	100	0	0	0	0	0	0	0	0	0	0	0	0	0
16.2	100	0	0	0	0	0	0	0	0	0	0	0	0	0
32.6	100	0	0	0	0	0	0	0	0	0	0	0	0	0
48.0	86.3	23.8	13.8	23.8	0	0	0	0	0	0	0	0	0	0
64.0	56.8	22.9	43.2	22.9	0	0	0	0	0	0	0	0	0	0
80.2	40.1	32.2	59.9	32.2	0	0	0	0	0	0	0	0	0	0
124.0	0	0	100	0	0	0	0	0	0	0	0	0	0	0
168.4	0	0	100	0	0	0	0	0	0	0	0	0	0	0
212.8	0	0	100	0	0	0	0	0	0	0	0	0	0	0
256.5	0	0	66.7	47.1	33.3	47.1	0	0	0	0	0	0	0	0
324.3	0	0	100	0	0.000*	0.000*	0	0	0	0	0	0	0	0
387.1	0	0	0	0	100	0	0	0	0	0	0	0	0	0
426.2	0	0	0	0	100	0	0	0	0	0	0	0	0	0
465.0	0	0	0	0	100	0	0	0	0	0	0	0	0	0
552.1	0	0	0	0	0	0	100	0	0	0	0	0	0	0
615.1	0	0	0	0	0	0	17.5	9.2	72.5	19.2	10	10	0	0
726.0	0	0	0	0	0	0	100	0	0	0	0	0	0	0
837.0	0	0	0	0	0	0	47.2	41	36.1	45.3	16.7	23.6	0	0
948.0	0	0	0	0	0	0	0	0	0	0	100	0	0	0
1059.0	0	0	0	0	0	0	0	0	0	0	100	0	0	0
1170.0	0	0	0	0	0	0	0	0	0	0	100	0	0	0
1451.0	0	0	0	0	0	0	0	0	0	0	50	0	50	0
1732.0	0	0	0	0	0	0	0	0	0	0	0	0	100	0
1732.0	0	0	0	0	0	0	0	0	0	0	0	0	100	0
1732.0	0	0	0	0	0	0	0	0	0	0	0	0	100	0
1732.0	0	0	0	0	0	0	0	0	0	0	0	0	100	0
1732.0	0	0	0	0	0	0	0	0	0	0	0	0	100	0
1732.0	0	0	0	0	0	0	0	0	0	0	0	0	100	0
1732.0	0	0	0	0	0	0	0	0	0	0	0	0	100	0
1732.0	0	0	0	0	0	0	0	0	0	0	0	0	100	0

Gaussian					
	L1	L2	L3f	L3m	P
				Ambiguous	
Best-fit values					
Amplitude	108	109.4	84.52	~ 216.0	112
Mean	208	403.8	700.1	~ 632.9	1148
SD	97.57	80.24	127.1	~ 12.07	209
Std. Error					
Amplitude	8.848	4.544	13.07	~ 4.196e+012	6.262
Mean	9.435	5.969	21.09	~ 1.938e+011	15.77
SD	8.329	4.76	20.83	~ 2.370e+010	12.71
95% Confidence Intervals					
Amplitude	89.88 to 126.2	100.0 to 118.7	57.70 to 111.3	(Very wide)	99.14 to 124.8
Mean	188.6 to 227.3	391.6 to 416.1	656.9 to 743.4	(Very wide)	1115 to 1180
SD	80.48 to 114.7	70.46 to 90.03	84.39 to 169.9	(Very wide)	182.9 to 235.0
Goodness of Fit					
df	27	26	27	27	27
R square	0.8578	0.9624	0.6759	0.7887	0.9359
Absolute SS	5510	1026	6546	1303	1804
Sy.x	14.29	6.283	15.57	6.947	8.175
Constraints					
SD	SD > 0.0	SD > 0.0	SD > 0.0	SD > 0.0	SD > 0.0
Number of points					
Analyzed	30	29	30	30	30

Modified Gaussian					
	L1	L2	L3	L3m	P
		Not converged	Ambiguous	Ambiguous	Ambiguous
Best-fit values					
a	= 100.0		= 100.0	= 100.0	= 100.0
b	214.4		~ 552.1	~ 615.8	~ 948.0
c	138.6		~ 1.000	~ 1.004	~ 1.000
d	8.05		~ 1.000	~ 1.040	~ 1.000
Std. Error					
a					
b	4.117		~ 7.945e+016	~ 3.571e+026	~ 5.224e+023
c	4.615		~ 4.895e+017	~ 4.958e+026	~ 2.018e+024
d	1.389		~ 1.184e+017	~ 1.223e+026	~ 4.285e+023
95% Confidence Intervals					
a	205.9 to 222.8		(Very wide)	(Very wide)	(Very wide)
b	129.1 to 148.1		(Very wide)	(Very wide)	(Very wide)
c	5.201 to 10.90		(Very wide)	(Very wide)	(Very wide)
d					
Goodness of Fit	27		27	27	27
df	0.9666		0.3795	0.7887	0.1872
R square	1294		12534	1303	22879
Absolute SS	6.922		21.55	6.947	29.11
Sy.x					
Constraints	a = 100.0	a = 100.0	a = 100.0	a = 100.0	a = 100.0
c	c > 0.0	c > 0.0	c > 0.0	c > 0.0	c > 0.0
d	d > 1.000	d > 1.000	d > 1.000	d > 1.000	d > 1.000
Number of points					
Analyzed	30	29	30	30	30

Cumulative Gaussian	
	A
	Perfect fit
Best-fit values	
Mean	1451
SD	10.16
Std. Error	
Mean	
SD	
95% Confidence Intervals	
Mean	
SD	
Goodness of Fit	
Degrees of Freedo	28
R square	1
Absolute Sum of S	0
Sy.x	
Number of points	
Analyzed	30

Reversed Cumulative Gaussian	
	egg
Best-fit values	
	71.83
	23.41
	0.7247
	1.198
95% Confidence Intervals	
	70.34 to 73.31
	20.96 to 25.86
Goodness of Fit	
	28
	0.9976
	83.68
	1.729
Runs	
	5
	5
Number of runs	4
P value (runs test)	0.1667
Deviation from Model	Not Significant
Number of points	
Analyzed	30

P. regina 15.0° C.

**P. regina 15.0° C**														
Dataset														
Time	egg		L1		L2		L3f		L3m		P		A	
	Mean	SE	Mean	SE	Mean	SE	Mean	SE	Mean	SE	Mean	SE	Mean	SE
0.0	100	0	0	0	0	0	0	0	0	0	0	0	0	0
8.1	100	0	0	0	0	0	0	0	0	0	0	0	0	0
16.1	100	0	0	0	0	0	0	0	0	0	0	0	0	0
24.1	100	0	0	0	0	0	0	0	0	0	0	0	0	0
32.1	100	0	0	0	0	0	0	0	0	0	0	0	0	0
16.2	89.1	18.9	10.9	18.9	0	0	0	0	0	0	0	0	0	0
62.2	48.3	24.4	51.7	24.4	0	0	0	0	0	0	0	0	0	0
85.3	10	17.3	90	17.3	0	0	0	0	0	0	0	0	0	0
104.4	13.6	11.9	86.4	11.9	0	0	0	0	0	0	0	0	0	0
129.2	0	0	100	0	0	0	0	0	0	0	0	0	0	0
151.2	0	0	94.4	9.6	5.6	9.6	0	0	0	0	0	0	0	0
181.1	0	0	0	0	100	0	0	0	0	0	0	0	0	0
212.1	0	0	0	0	73.3	28.3	26.7	28.3	0	0	0	0	0	0
243.0	0	0	0	0	27.9	24.5	72.1	24.5	0	0	0	0	0	0
274.2	0	0	0	0	25	25	75	25	0	0	0	0	0	0
305.1	0	0	0	0	0	0	100	0	0	0	0	0	0	0
383.9	0	0	0	0	0	0	84.4	27.1	15.6	27.1	0	0	0	0
411.1	0	0	0	0	0	0	50	40.8	27.8	20.8	22.2	31.4	0	0
470.1	0	0	0	0	0	0	15	16.6	32.5	34.2	52.5	47.6	0	0
525.0	0	0	0	0	0	0	0	0	22.9	30.8	77.1	30.8	0	0
580.1	0	0	0	0	0	0	0	0	7.1	12.4	92.9	12.4	0	0
721.0	0	0	0	0	0	0	0	0	5.6	5.6	94.4	5.6	0	0
865.5	0	0	0	0	0	0	0	0	0	0	37.2	38.5	62.8	38.5
1005.0	0	0	0	0	0	0	0	0	0	0	64.4	22	35.6	22
1146.0	0	0	0	0	0	0	0	0	0	0	39.7	2.7	60.3	2.7
1295.5	0	0	0	0	0	0	0	0	0	0	0	0	100	0
1295.5	0	0	0	0	0	0	0	0	0	0	0	0	100	0
1295.5	0	0	0	0	0	0	0	0	0	0	0	0	100	0
1295.5	0	0	0	0	0	0	0	0	0	0	0	0	100	0
1295.5	0	0	0	0	0	0	0	0	0	0	0	0	100	0
1295.5	0	0	0	0	0	0	0	0	0	0	0	0	100	0

Gaussian					
	L1	L2	L3f	L3m	P
Best-fit values					
Amplitude	109.2	111.8	109	34.36	94.93
Mean	115.4	194.1	327.2	462.7	719.2
SD	38.19	22.08	70.6	66.07	205.4
Std. Error					
Amplitude	5.953	8.079	4.193	1.134	9.204
Mean	2.488	1.52	2.408	2.272	20.33
SD	2.511	1.962	2.724	2.601	19.07
95% Confidence Intervals					
Amplitude	96.97 to 121.3	95.22 to 128.3	100.4 to 117.5	32.04 to 36.68	76.08 to 113.8
Mean	110.3 to 120.5	191.0 to 197.2	322.3 to 332.2	458.0 to 467.3	677.5 to 760.8
SD	33.04 to 43.33	18.07 to 26.10	65.02 to 76.18	60.74 to 71.39	166.3 to 244.4
Goodness of Fit					
df	28	28	28	28	28
R square	0.9367	0.9227	0.9743	0.9795	0.825
Absolute SS	1976	1165	659	46.72	4620
Sy.x	8.4	6.451	4.851	1.292	12.85
Constraints					
SD	SD > 0.0	SD > 0.0	SD > 0.0	SD > 0.0	SD > 0.0
Number of points					
Analyzed	31	31	31	31	31

Modified Gaussian					
	L1	L2	L3	L3m	P
	Ambiguous	Ambiguous		Ambiguous	Not converged
Best-fit values					
a	92.7	~ 100.0	95.65	~ 32.50	
b	~ 118.2	~ 181.1	324.1	~ 470.1	
c	~ 55.61	~ 1.002	84.62	~ 1.000	
d	~ 23.61	~ 1.000	3.115	~ 1.000	
Std. Error					
a	1.979	~ 7.401e+006	3.724	~ 1.195e+012	
b	~ 3357	~ 1.100e+009	2.056	~ 1.518e+017	
c	~ 3689	~ 6.843e+009	3.515	~ 2.997e+017	
d	~ 22841		0.3569	~ 7.413e+016	
95% Confidence Intervals					
a	88.64 to 96.76	(Very wide)	88.00 to 103.3	(Very wide)	
b	(Very wide)	(Very wide)	319.9 to 328.4	(Very wide)	
c	(Very wide)	(Very wide)	77.41 to 91.84	(Very wide)	
d	(Very wide)		2.382 to 3.847	(Very wide)	
Goodness of Fit					
df	27	27	27	27	
R square	0.9929	0.5484	0.9824	0.2877	
Absolute SS	222	6808	451.7	1622	
Sy.x	2.867	15.88	4.09	7.752	
Constraints					
c	c > 0.0	c > 0.0	c > 0.0	c > 0.0	c > 0.0
d	d > 1.000	d > 1.000	d > 1.000	d > 1.000	d > 1.000
Number of points					
Analyzed	31	31	31	31	31

Cumulative Gaussian	
	A
Best-fit values	
Mean	972.1
SD	214.5
Std. Error	
Mean	27.09
SD	30.54
95% Confidence Intervals	
Mean	916.7 to 1028
SD	152.0 to 277.0
Goodness of Fit	
Degrees of Freedc	29
R square	0.956
Absolute Sum of S	2210
Sy.x	8.731
Number of points	
Analyzed	31

Reversed Cumulative Gaussian	
	egg
Best-fit values	
	62.25
	18.64
	1.31
	1.87
95% Confidence Intervals	
	59.57 to 64.93
	14.81 to 22.46
Goodness of Fit	
	29
	0.9936
	295.1
	3.19
Runs	
	6
	7
Number of runs	6
P value (runs test)	0.296
Deviation from Model	Not Significant
Number of points	
Analyzed	31

P. regina 17.5° C.

P. regina 17.5° C														
Dataset														
Time	egg		L1		L2		L3f		L3m		P		A	
	Mean	SE	Mean	SE	Mean	SE	Mean	SE	Mean	SE	Mean	SE	Mean	SE
0.0	100	0	0	0	0	0	0	0	0	0	0	0	0	0
5.1	100	0	0	0	0	0	0	0	0	0	0	0	0	0
10.1	100	0	0	0	0	0	0	0	0	0	0	0	0	0
15.1	100	0	0	0	0	0	0	0	0	0	0	0	0	0
20.0	100	0	0	0	0	0	0	0	0	0	0	0	0	0
25.1	100	0	0	0	0	0	0	0	0	0	0	0	0	0
40.3	43.8	19.3	56.2	19.3	0	0	0	0	0	0	0	0	0	0
51.2	25.8	12.6	74.2	12.6	0	0	0	0	0	0	0	0	0	0
70.2	0	0	100	0	0	0	0	0	0	0	0	0	0	0
85.3	0	0	100	0	0	0	0	0	0	0	0	0	0	0
100.3	0	0	94.2	10	5.8	10	0	0	0	0	0	0	0	0
121.5	0	0	54.4	41.5	45.6	41.5	0	0	0	0	0	0	0	0
142.2	0	0	34.4	38.9	65.6	38.9	0	0	0	0	0	0	0	0
163.1	0	0	0	0	100	0	0	0	0	0	0	0	0	0
184.3	0	0	0	0	100	0	0	0	0	0	0	0	0	0
205.2	0	0	0	0	83.7	10.7	16.3	10.7	0	0	0	0	0	0
242.1	0	0	0	0	5	8.7	95	8.7	0	0	0	0	0	0
279.1	0	0	0	0	0	0	26.9	31.5	73.1	31.5	0	0	0	0
316.2	0	0	0	0	0	0	26.8	23.9	57.8	18.5	15.4	20.5	0	0
353.0	0	0	0	0	0	0	0	0	0	0	100	0	0	0
390.1	0	0	0	0	0	0	0	0	0	0	100	0	0	0
484.1	0	0	0	0	0	0	0	0	0	0	91.3	15	8.7	15
578.0	0	0	0	0	0	0	0	0	0	0	78.6	21.4	21.4	21.4
671.5	0	0	0	0	0	0	0	0	1.5	2.5	13.8	8.3	84.8	7.2
766.0	0	0	0	0	0	0	0	0	0	0	28.1	27.9	71.9	27.9
859.0	0	0	0	0	0	0	0	0	0	0	0	0	100	0
859.0	0	0	0	0	0	0	0	0	0	0	0	0	100	0
859.0	0	0	0	0	0	0	0	0	0	0	0	0	100	0
859.0	0	0	0	0	0	0	0	0	0	0	0	0	100	0
859.0	0	0	0	0	0	0	0	0	0	0	0	0	100	0
859.0	0	0	0	0	0	0	0	0	0	0	0	0	100	0

Gaussian					
	L1	L2	L3	L3m	P
				Ambiguous	
Best-fit values					
Amplitude	108.8	107.5	95.82	~ 245.4	115.9
Mean	84.47	174.9	245.4	~ 296.8	466.3
SD	31.51	35.06	21.49	~ 11.39	103.3
Std. Error					
Amplitude	4.504	2.922	5.213	~ 3224	10.35
Mean	1.602	1.213	2.012	~ 8.153	9.41
SD	1.425	1.237	1.304	~ 56.54	9.224
95% Confidence Intervals					
Amplitude	99.57 to 118.0	101.5 to 113.5	85.15 to 106.5	(Very wide)	94.66 to 137.1
Mean	81.19 to 87.75	172.4 to 177.4	241.3 to 249.5	(Very wide)	447.0 to 485.6
SD	28.59 to 34.43	32.53 to 37.59	18.82 to 24.16	(Very wide)	84.39 to 122.2
Goodness of Fit					
df	28	28	28	28	28
R square	0.9586	0.9835	0.929	0.9997	0.8603
Absolute SS	1374	463.8	700.1	2.25	4169
Sy.x	7.005	4.07	5.001	0.2835	12.2
Constraints					
SD	SD > 0.0	SD > 0.0	SD > 0.0	SD > 0.0	SD > 0.0
Number of points					
Analyzed	31	31	31	31	31

Modified Gaussian					
	L1	L2	L3	L3m	P
			Ambiguous	Ambiguous	Ambiguous
Best-fit values					
a	98.55	99.94	~ 95.00	~ 73.10	92.48
b	84.91	174.4	~ 242.1	~ 279.1	~ 493.8
c	39.44	40.6	~ 1.001	~ 1.000	~ 175.0
d	3.317	2.767	~ 1.000	~ 1.000	~ 86.57
Std. Error					
a	3.485	2.752	~ 2.024e+006	~ 2.441e+009	3.68
b	1.155	1.083	~ 1.128e+009	~ 2.352e+012	~ 72339
c	1.702	1.691	~ 8.151e+010	~ 1.699e+014	~ 3.135e+006
d	0.3956	0.2535		~ 4.703e+013	~ 1.028e+008
95% Confidence Intervals					
a	91.40 to 105.7	94.29 to 105.6	(Very wide)	(Very wide)	84.92 to 100.0
b	82.54 to 87.28	172.2 to 176.6	(Very wide)	(Very wide)	(Very wide)
c	35.94 to 42.93	37.13 to 44.07	(Very wide)	(Very wide)	(Very wide)
d	2.505 to 4.129	2.247 to 3.287		(Very wide)	(Very wide)
Goodness of Fit					
df	27	27	27	27	27
R square	0.9759	0.9887	0.8267	0.5884	0.9633
Absolute SS	799.6	318.4	1708	3343	1097
Sy.x	5.442	3.434	7.952	11.13	6.373
Constraints					
c	c > 0.0	c > 0.0	c > 0.0	c > 0.0	c > 0.0
d	d > 1.000	d > 1.000	d > 1.000	d > 1.000	d > 1.000
Number of points					
Analyzed	31	31	31	31	31

Cumulative Gaussian	
	A
Best-fit values	
Mean	625.5
SD	93.19
Std. Error	
Mean	9.254
SD	12.71
95% Confidence Intervals	
Mean	606.6 to 644.4
SD	67.21 to 119.2
Goodness of Fit	
df	29
R square	0.9848
Absolute SS	805.8
Sy.x	5.271
Number of points	
Analyzed	31

Reversed Cumulative Gaussian	
	egg
Best-fit values	
Mean	40.97
SD	11.17
Std. Error	
Mean	0.5917
SD	0.8495
95% Confidence Intervals	
Mean	39.76 to 42.18
SD	9.431 to 12.91
Goodness of Fit	
df	29
R square	0.9957
Absolute SS	205.7
Sy.x	2.663
Runs test	
Points above curve	7
Points below curve	5
Number of runs	4
P value (runs test)	0.0758
Deviation from Model	Not Significant
Number of points	
Analyzed	31

P. regina 20.0° C.

P. regina 20.0° C

Dataset

Time	egg Mean	egg SE	L1 Mean	L1 SE	L2 Mean	L2 SE	L3f Mean	L3f SE	L3m Mean	L3m SE	P Mean	P SE	A Mean	A SE
0.0	100	0	0	0	0	0	0	0	*0*	*0*	0	0	0	0
4.0	100	0	0	0	0	0	0	0	*0*	*0*	0	0	0	0
8.1	100	0	0	0	0	0	0	0	*0*	*0*	0	0	0	0
12.1	100	0	0	0	0	0	0	0	*0*	*0*	0	0	0	0
16.0	100	0	0	0	0	0	0	0	*0*	*0*	0	0	0	0
20.2	95	8.7	5	8.7	0	0	0	0	*0*	*0*	0	0	0	0
31.1	1.9	3.3	98.1	3.3	0	0	0	0	*0*	*0*	0	0	0	0
42.2	0	0	100	0	0	0	0	0	*0*	*0*	0	0	0	0
53.2	0	0	100	0	0	0	0	0	*0*	*0*	0	0	0	0
64.8	0	0	100	0	0	0	0	0	*0*	*0*	0	0	0	0
75.1	0	0	44.2	42.7	55.8	42.7	0	0	*0*	*0*	0	0	0	0
91.2	0	0	3.6	3.7	96.4	3.7	0	0	*0*	*0*	0	0	0	0
107.2	0	0	0	0	100	0	0	0	*0*	*0*	0	0	0	0
123.2	0	0	0	0	31.4	40	68.6	40	0	0	0	0	0	0
139.0	0	0	0	0	25	43.3	75	43.3	0	0	0	0	0	0
155.2	0	0	0	0	23.8	35.2	76.2	35.2	0	0	0	0	0	0
183.0	0	0	0	0	0	0	76.3	41	23.7	41	0	0	0	0
211.2	0	0	0	0	0	0	37.7	38.1	62.3	38.1	0	0	0	0
215.0	0	0	0	0	0	0	25	43.3	*8.6*	*8.7*	66.4	39	0	0
267.0	0	0	0	0	0	0	4	4.6	*38.3*	*29.4*	57.7	33.7	0	0
295.0	0	0	0	0	0	0	17.3	26.8	*82.7*	*26.8*	0.000*	0	0	0
365.0	0	0	0	0	0	0	0	0	*3.6*	*6.2*	96.4	6.2	0	0
435.0	0	0	0	0	0	0	0	0	*0*	*0*	30.6	41.1	69.4	41.1
505.0	0	0	0	0	0	0	0	0	*2.1*	*3.6*	34.6	33.7	63.3	37.3
575.0	0	0	0	0	0	0	0	0	*0*	*0*	33.5	39	66.5	39
648.6	0	0	0	0	0	0	0	0	*0*	*0*	0	0	100	0
648.6	0	0	0	0	0	0	0	0	*0*	*0*	0	0	100	0
648.6	0	0	0	0	0	0	0	0	*0*	*0*	0	0	100	0
648.6	0	0	0	0	0	0	0	0	*0*	*0*	0	0	100	0
648.6	0	0	0	0	0	0	0	0	*0*	*0*	0	0	100	0
648.6	0	0	0	0	0	0	0	0	*0*	*0*	0	0	100	0

Gaussian

	L1	L2	L3	L3m	P
Best-fit values					
Amplitude	118.5	109.4	89.98	62.12	93.32
Mean	50.84	98.6	163	276.3	341.9
SD	17.35	17.35	33.66	55.17	81.41
Std. Error					
Amplitude	7.08	6.144	5.969	7.64	12.29
Mean	1.189	1.106	2.275	9.62	10.15
SD	1.122	1.056	2.311	8.167	9.383
95% Confidence Intervals					
Amplitude	104.0 to 133.0	96.84 to 122.0	77.75 to 102.2	46.47 to 77.77	68.09 to 118.5
Mean	48.41 to 53.28	96.33 to 100.9	158.3 to 167.6	256.6 to 296.0	321.1 to 362.8
SD	15.05 to 19.65	15.19 to 19.52	28.92 to 38.39	38.44 to 71.89	62.15 to 100.7
Goodness of Fit					
df	28	28	28	28	27
R square	0.9248	0.9343	0.9143	0.7325	0.7447
Absolute SS	2635	1381	1686	3013	4311
Sy.x	9.701	7.023	7.759	10.37	12.64
Constraints					
SD	SD > 0.0	SD > 0.0	SD > 0.0	SD > 0.0	SD > 0.0
Number of points					
Analyzed	31	31	31	31	30

Modified Gaussian

	L1	L2	L3	L3m	P
				Ambiguous	Ambiguous
Best-fit values					
a	100.1	131.1	76.14	~ 82.70	~ 96.40
b	49.31	99.29	163.8	~ 295.0	~ 365.0
c	24.63	12.31	46.47	~ 1.001	~ 1.000
d	10.71	1.298	9.566	~ 1.000	~ 1.000
Std. Error					
a	0.4256	30	2.109	~ 2.106e+014	~ 1.159e+016
b	0.1376	0.927	0.7686	~ 2.954e+016	~ 3.935e+021
c	0.2234	5.271	0.8956	~ 6.432e+015	~ 3.653e+022
d	0.8617	0.4452	1.499		~ 8.612e+021
95% Confidence Intervals					
a	99.23 to 101.0	69.57 to 192.7	71.81 to 80.47	(Very wide)	(Very wide)
b	49.03 to 49.60	97.39 to 101.2	162.2 to 165.3	(Very wide)	(Very wide)
c	24.17 to 25.09	1.491 to 23.12	44.63 to 48.30	(Very wide)	(Very wide)
d	8.937 to 12.47	1.000 to 2.212	6.491 to 12.64		(Very wide)
Goodness of Fit					
df	27	27	27	27	26
R square	0.9996	0.9358	0.9819	0.4671	0.3491
Absolute SS	13.04	1350	356.6	6001	10994
Sy.x	0.695	7.072	3.634	14.91	20.56
Constraints					
c	c > 0.0	c > 0.0	c > 0.0	c > 0.0	c > 0.0
d	d > 1.000	d > 1.000	d > 1.000	d > 1.000	d > 1.000
Number of points					
Analyzed	31	31	31	31	30

Cumulative Gaussian

	A
Best-fit values	
Mean	456.2
SD	101.6
Std. Error	
Mean	11.77
SD	14.18
95% Confidence Intervals	
Mean	432.1 to 480.3
SD	72.64 to 130.6
Goodness of Fit	
df	29
R square	0.968
Absolute SS	1684
Sy.x	7.621
Number of points	
Analyzed	31

Reversed Cumulative Gaussian

	egg
Best-fit values	
Mean	25.02
SD	2.929
Std. Error	
Mean	0.006234
SD	0.003609
95% Confidence Intervals	
Mean	25.01 to 25.03
SD	2.921 to 2.936
Goodness of Fit	
df	29
R square	1
Absolute SS	0.01089
Sy.x	0.01937
Runs test	
Points above curve	5
Points below curve	2
Number of runs	4
P value (runs test)	0.7143
Deviation from Model	Not Significant
Number of points	
Analyzed	31

P. regina 22.5 C.

P. regina 22.5° C

Dataset														
Time	egg		L1		L2		L3f		L3m		P		A	
	Mean	SE	Mean	SE	Mean	SE	Mean	SE	Mean	SE	Mean	SE	Mean	SE
0.0	100	0	0	0	0	0	0	0	0	0	0	0	0	0
3.0	100	0	0	0	0	0	0	0	0	0	0	0	0	0
6.0	100	0	0	0	0	0	0	0	0	0	0	0	0	0
9.1	100	0	0	0	0	0	0	0	0	0	0	0	0	0
12.1	100	0	0	0	0	0	0	0	0	0	0	0	0	0
15.3	72.2	24.6	27.8	24.6	0	0	0	0	0	0	0	0	0	0
24.3	0	0	100	0	0	0	0	0	0	0	0	0	0	0
32.8	0	0	100	0	0	0	0	0	0	0	0	0	0	0
42.1	0	0	100	0	0	0	0	0	0	0	0	0	0	0
51.2	0	0	30.1	40.6	69.9	40.6	0	0	0	0	0	0	0	0
60.2	0	0	1.5	2.5	98.5	2.5	0	0	0	0	0	0	0	0
73.0	0	0	0	0	100	0	0	0	0	0	0	0	0	0
86.1	0	0	0	0	54.7	39.3	45.3	39.3	0	0	0	0	0	0
99.0	0	0	0	0	41.1	39.2	58.9	39.2	0	0	0	0	0	0
112.1	0	0	0	0	0	0	94.9	5.1	5.1	5.1	0	0	0	0
125.1	0	0	0	0	0	0	91.3	10.2	8.8	10.2	0	0	0	0
147.2	0	0	0	0	0	0	3.9	6.8	96.1	6.8	0	0	0	0
169.0	0	0	0	0	0	0	14.1	24.4	54	15.7	31.9	23.5	0	0
191.1	0	0	0	0	0	0	3.1	5.4	23.4	37.1	73.4	42.5	0	0
213.1	0	0	0	0	0	0	0	0	3.1	3.1	96.9	3.1	0	0
235.1	0	0	0	0	0	0	0	0	1.4	2.4	98.6	2.4	0	0
291.0	0	0	0	0	0	0	0	0	0	0	100	0	0	0
347.0	0	0	0	0	0	0	0	0	0	0	61.3	38.9	38.7	38.9
402.0	0	0	0	0	0	0	0	0	3	3	7.1	2	89.9	2.7
458.9	0	0	0	0	0	0	0	0	0	0	2.1	3.6	97.9	3.6
523.0	0	0	0	0	0	0	0	0	0	0	0	0	100	0
523.0	0	0	0	0	0	0	0	0	0	0	0	0	100	0
523.0	0	0	0	0	0	0	0	0	0	0	0	0	100	0
523.0	0	0	0	0	0	0	0	0	0	0	0	0	100	0
523.0	0	0	0	0	0	0	0	0	0	0	0	0	100	0
523.0	0	0	0	0	0	0	0	0	0	0	0	0	100	0

Gaussian					
	L1	L2	L3	L3m	P
Best-fit values					
Amplitude	118.9	109.1	98.7	103.7	119.4
Mean	33.89	69.44	114.2	153.5	264.9
SD	10.78	15.78	17.82	14.25	61.9
Std. Error					
Amplitude	5.403	5.99	4.473	5.283	5.277
Mean	0.5593	0.941	1.084	0.8066	2.854
SD	0.51	0.9512	1.063	0.8896	2.827
95% Confidence Intervals					
Amplitude	107.8 to 130.0	96.78 to 121.3	89.54 to 107.9	92.83 to 114.5	108.6 to 130.2
Mean	32.74 to 35.03	67.51 to 71.37	112.0 to 116.4	151.9 to 155.2	259.1 to 270.8
SD	9.733 to 11.82	13.83 to 17.73	15.64 to 20.00	12.42 to 16.07	56.12 to 67.69
Goodness of Fit					
df	28	28	28	28	28
R square	0.9559	0.9403	0.9549	0.958	0.9624
Absolute SS	1214	1491	900.6	486.9	1210
Sy.x	6.586	7.297	5.671	4.17	6.574
Constraints					
SD	SD > 0.0	SD > 0.0	SD > 0.0	SD > 0.0	SD > 0.0
Number of points					
Analyzed	31	31	31	31	31

Modified Gaussian					
	L1	L2	L3	L3m	P
	Ambiguous			Hit constraint	
Best-fit values					
a	100	103	94.9	174.3	99.74
b	~ 33.29	69.89	113.8	154.8	265.3
c	~ 16.97	17.65	19.34	6.273	82.13
d	~ 16.24	2.501	2.364	~ 1.000	5.427
Std. Error					
a	0.2828	6.964	5.679	106	1.048
b	~ 0.7545	0.9257	1.159	0.999	0.5366
c	~ 20.22	1.61	2.008	6.026	0.6642
d	~ 345.9	0.4813	0.4405		0.2939
95% Confidence Intervals					
a	99.42 to 100.6	88.71 to 117.3	83.25 to 106.6	-43.33 to 391.8	97.59 to 101.9
b	(Very wide)	67.99 to 71.79	111.4 to 116.2	152.8 to 156.9	264.2 to 266.4
c	(Very wide)	14.35 to 20.96	15.22 to 23.46	0.0 to 18.64	80.77 to 83.50
d	(Very wide)	1.513 to 3.489	1.460 to 3.268		4.824 to 6.030
Goodness of Fit					
df	27	27	27	27	27
R square	0.9999	0.9433	0.9559	0.9768	0.9979
Absolute SS	2.25	1417	880.1	268.9	68.59
Sy.x	0.2887	7.244	5.709	3.156	1.594
Constraints					
c	c > 0.0	c > 0.0	c > 0.0	c > 0.0	c > 0.0
d	d > 1.000	d > 1.000	d > 1.000	d > 1.000	d > 1.000
Number of points					
Analyzed	31	31	31	31	31

Cumulative Gaussian	
	A
Best-fit values	
Mean	357.4
SD	33.79
Std. Error	
Mean	0.5036
SD	0.7702
95% Confidence Intervals	
Mean	356.3 to 358.4
SD	32.22 to 35.37
Goodness of Fit	
df	29
R square	0.9998
Absolute SS	11.17
Sy.x	0.6205
Number of points	
Analyzed	31

Reversed Cumulative Gaussian	
	egg
	Interrupted
Best-fit values	
Mean	15.6
SD	0.5076
Std. Error	
Mean	
SD	
95% Confidence Intervals	
Mean	
SD	
Goodness of Fit	
df	
R square	
Absolute SS	
Sy.x	
Runs test	
Points above curve	
Points below curve	
Number of runs	
P value (runs test)	
Deviation from Model	
Number of points	
Analyzed	31

P. regina 25.0° C.

***P. regina* 25.0° C**														
Dataset														
Time	egg		L1		L2		L3f		L3m		P		A	
	Mean	SE	Mean	SE	Mean	SE	Mean	SE	Mean	SE	Mean	SE	Mean	SE
0.0	100	0	0	0	0	0	0	0	0	0	0	0	0	0
3.1	100	0	0	0	0	0	0	0	0	0	0	0	0	0
6.0	100	0	0	0	0	0	0	0	0	0	0	0	0	0
9.1	100	0	0	0	0	0	0	0	0	0	0	0	0	0
12.1	100	0	0	0	0	0	0	0	0	0	0	0	0	0
15.1	55.3	31.4	44.7	31.4	0	0	0	0	0	0	0	0	0	0
22.2	0	0	100	0	0	0	0	0	0	0	0	0	0	0
25.2	0	0	100	0	0	0	0	0	0	0	0	0	0	0
36.1	0	0	100	0	0	0	0	0	0	0	0	0	0	0
43.1	0	0	53.5	29.9	46.5	29.9	0	0	0	0	0	0	0	0
50.1	0	0	2.1	3.6	97.9	3.6	0	0	0	0	0	0	0	0
60.0	0	0	0	0	100	0	0	0	0	0	0	0	0	0
70.1	0	0	0	0	48.4	14.9	51.6	14.9	0	0	0	0	0	0
80.3	0	0	0	0	23.6	33.1	76.4	33.1	0	0	0	0	0	0
90.1	0	0	0	0	5	8.7	95	8.7	0	0	0	0	0	0
100.1	0	0	0	0	0	0	96.3	6.5	3.8	6.5	0	0	0	0
118.1	0	0	0	0	0	0	70.1	30.9	29.9	30.9	0	0	0	0
136.1	0	0	0	0	0	0	10.7	18.6	88	17.9	1.3	2.3	0	0
150.6	0	0	0	0	0	0	45	45.6	25.9	32.1	29.1	27.8	0	0
173.2	0	0	0	0	0	0	0	0	2.5	4.3	97.5	4.3	0	0
191.1	0	0	0	0	0	0	0	0	0	0	100	0	0	0
238.2	0	0	0	0	0	0	0	0	0	0	100	0	0	0
285.0	0	0	0	0	0	0	0	0	0	0	30.4	41.1	69.6	41.1
332.0	0	0	0	0	0	0	0	0	0	0	0	0	100	0
379.5	0	0	0	0	0	0	0	0	0	0	0	0	100	0
379.5	0	0	0	0	0	0	0	0	0	0	0	0	100	0
379.5	0	0	0	0	0	0	0	0	0	0	0	0	100	0
379.5	0	0	0	0	0	0	0	0	0	0	0	0	100	0
379.5	0	0	0	0	0	0	0	0	0	0	0	0	100	0
379.5	0	0	0	0	0	0	0	0	0	0	0	0	100	0
379.5	0	0	0	0	0	0	0	0	0	0	0	0	100	0

Gaussian					
	L1	L2	L3	L3m	P
Best-fit values					
Amplitude	127.5	110.8	102.2	89.91	128
Mean	30.08	56.87	98.91	133.8	212.8
SD	9.054	10.39	21.43	10.64	38.54
Std. Error					
Amplitude	5.614	4.059	6.421	0.8225	5.996
Mean	0.3567	0.4117	1.638	0.1326	1.741
SD	0.3896	0.4196	1.552	0.09909	1.883
95% Confidence Intervals					
Amplitude	116.0 to 139.0	102.5 to 119.1	89.00 to 115.3	88.23 to 91.59	115.7 to 140.2
Mean	29.35 to 30.81	56.03 to 57.72	95.56 to 102.3	133.6 to 134.1	209.2 to 216.4
SD	8.256 to 9.852	9.532 to 11.25	18.25 to 24.60	10.44 to 10.85	34.68 to 42.40
Goodness of Fit					
df	28	28	28	28	28
R square	0.9671	0.9732	0.9149	0.9981	0.9635
Absolute SS	976	571.7	2337	16.15	991.2
Sy.x	5.904	4.519	9.136	0.7594	5.95
Constraints					
SD	SD > 0.0	SD > 0.0	SD > 0.0	SD > 0.0	SD > 0.0
Number of points					
Analyzed	31	31	31	31	31

Modified Gaussian					
	L1	L2	L3	L3m	P
	Ambiguous				
Best-fit values					
a	100	106.2	94.36	97.55	100
b	~ 29.22	57	97.59	133.7	217.9
c	~ 13.67	11.28	24.46	8.088	61.14
d	~ 14.88	2.448	3.533	1.317	9.378
Std. Error					
a	0.392	4.937	6.45	0.8062	0.1728
b	~ 2.515	0.3957	1.478	0.06143	0.03397
c	~ 6.840	0.7363	2.039	0.1588	0.1775
d	~ 315.1	0.3591	0.8644	0.02587	0.277
95% Confidence Intervals					
a	99.20 to 100.8	96.04 to 116.3	81.13 to 107.6	95.89 to 99.20	99.69 to 100.4
b	(Very wide)	56.19 to 57.81	94.56 to 100.6	133.5 to 133.8	217.9 to 218.0
c	(Very wide)	9.767 to 12.79	20.28 to 28.65	7.762 to 8.414	60.78 to 61.51
d	(Very wide)	1.711 to 3.185	1.760 to 5.307	1.264 to 1.370	8.810 to 9.947
Goodness of Fit					
df	27	27	27	27	27
R square	0.9999	0.9746	0.9195	0.9998	0.9999
Absolute SS	4.41	542.3	2211	1.682	1.583
Sy.x	0.4041	4.482	9.049	0.2496	0.2421
Constraints					
c	c > 0.0	c > 0.0	c > 0.0	c > 0.0	c > 0.0
d	d > 1.000	d > 1.000	d > 1.000	d > 1.000	d > 1.000
Number of points					
Analyzed	31	31	31	31	31

Cumulative Gaussian	
	A
	Interrupted
Best-fit values	
Mean	281.8
SD	6.312
Std. Error	
Mean	
SD	
95% Confidence Intervals	
Mean	
SD	
Goodness of Fit	
df	
R square	
Absolute SS	
Sy.x	
Number of points	
Analyzed	31

Reversed Cumulative Gaussian	
	egg
	Ambiguous
Best-fit values	
Mean	~ 15.16
SD	~ 0.4440
Std. Error	
Mean	
SD	
95% Confidence Intervals	
Mean	
SD	
Goodness of Fit	
Degrees of Freedom	29
R square	1
Absolute SS	0
Sy.x	
Runs test	
Points above curve	
Points below curve	
Number of runs	
P value (runs test)	
Deviation from Model	
Number of points	
Analyzed	31

P. regina 27.5° C.

***P. regina* 27.5° C**														
Dataset														
Time	egg		L1		L2		L3f		L3m		P		A	
	Mean	SE	Mean	SE	Mean	SE	Mean	SE	Mean	SE	Mean	SE	Mean	SE
0	100	0	0	0	0	0	0	0	0	0	0	0	0	0
2.0	100	0	0	0	0	0	0	0	0	0	0	0	0	0
4.0	100	0	0	0	0	0	0	0	0	0	0	0	0	0
6.1	100	0	0	0	0	0	0	0	0	0	0	0	0	0
8.1	100	0	0	0	0	0	0	0	0	0	0	0	0	0
10.1	100	0	0	0	0	0	0	0	0	0	0	0	0	0
16.2	0	0	100	0	0	0	0	0	0	0	0	0	0	0
22.2	0	0	100	0	0	0	0	0	0	0	0	0	0	0
29.1	0	0	100	0	0	0	0	0	0	0	0	0	0	0
34.2	0	0	17.7	12.7	82.3	12.7	0	0	0	0	0	0	0	0
40.1	0	0	0	0	100	0	0	0	0	0	0	0	0	0
49.1	0	0	0	0	100	0	0	0	0	0	0	0	0	0
58.0	0	0	0	0	1.3	2.3	98.7	2.3	0	0	0	0	0	0
67.2	0	0	0	0	0	0	100	0	0	0	0	0	0	0
76.3	0	0	0	0	1.9	2.6	98.1	2.6	0	0	0	0	0	0
85.1	0	0	0	0	0	0	55.3	44.7	44.7	44.7	0	0	0	0
101.1	0	0	0	0	0	0	40.9	38	59.1	38	0	0	0	0
117.1	0	0	0	0	0	0	0	0	79.5	25	20.5	25	0	0
133.1	0	0	0	0	0	0	0	0	5.6	5.7	94.4	5.7	0	0
164.8	0	0	0	0	0	0	0	0	0	0	100	0	0	0
205.1	0	0	0	0	0	0	0	0	0	0	100	0	0	0
245.0	0	0	0	0	0	0	0	0	0	0	33.3	30.4	66.7	30.4
285.0	0	0	0	0	0	0	0	0	0	0	3.1	5.4	96.9	5.4
325.0	0	0	0	0	0	0	0	0	0	0	0	0	100	0
325.0	0	0	0	0	0	0	0	0	0	0	0	0	100	0
325.0	0	0	0	0	0	0	0	0	0	0	0	0	100	0
325.0	0	0	0	0	0	0	0	0	0	0	0	0	100	0
325.0	0	0	0	0	0	0	0	0	0	0	0	0	100	0
325.0	0	0	0	0	0	0	0	0	0	0	0	0	100	0
325.0	0	0	0	0	0	0	0	0	0	0	0	0	100	0
461.0	0	0	0	0	0	0	0	0	0	0	0	0	100	0

Gaussian					
	L1	L2	L3	L3m	P
	Interrupted	Ambiguous		Ambiguous	Not converged
Best-fit values					
Amplitude	100	100	99.05	69.3	
Mean	22.55	~ 44.42	68.94	~ 107.7	
SD	11.3	~ 11.14	15.97	~ 22.83	
Std. Error	40.43	~ 10.91	13.02	~ 15.35	
Amplitude					
Mean		0.4781	5.692	2.843	
SD		~ 19.91	18.7	~ 8276	
95% Confidence Intervals		~ 10.17	17.45	~ 7159	
Amplitude		~ 131.0	100.5	~ 93344	
Mean					
SD		99.02 to 101.0	87.35 to 110.8	63.45 to 75.15	
Goodness of Fit		(Very wide)	30.50 to 107.4	(Very wide)	
df		(Very wide)	0.0 to 51.84	(Very wide)	
R square		(Very wide)	1.000 to 219.7	(Very wide)	
Absolute SS					
Sy.x		26	26	26	
Constraints		0.9999	0.9422	0.9805	
SD		3.61	1675	208.1	
		0.3726	8.025	2.829	
Number of points					
Analyzed	c > 0.0	c > 0.0	c > 0.0	c > 0.0	c > 0.0
	d > 1.000	d > 1.000	d > 1.000	d > 1.000	d > 1.000
	30	30	30	30	30

Modified Gaussian					
	L1	L2	L3	L3m	P
	Interrupted	Ambiguous		Ambiguous	
Best-fit values					
a	100	100	99.05	69.3	100
b	22.55	~ 44.42	68.94	~ 107.7	182
c	11.3	~ 11.14	15.97	~ 22.83	58.86
d	40.82	~ 10.91	13.03	~ 15.35	11.72
Std. Error					
a		0.4778	5.692	2.843	0.43
b		~ 19.84	18.75	~ 8276	0.07342
c		~ 10.14	17.49	~ 7159	0.2079
d		~ 130.5	100.8	~ 93345	0.4683
95% Confidence Intervals					
a		99.02 to 101.0	87.35 to 110.8	63.45 to 75.15	99.12 to 100.9
b		(Very wide)	30.40 to 107.5	(Very wide)	181.9 to 182.2
c		(Very wide)	0.0 to 51.93	(Very wide)	58.43 to 59.29
d		(Very wide)	1.000 to 220.2	(Very wide)	10.75 to 12.68
Goodness of Fit					
df		26	26	26	26
R square		0.9999	0.9422	0.9805	0.9996
Absolute SS		3.61	1675	208.1	9.61
Sy.x		0.3726	8.025	2.829	0.608
Constraints					
c	c > 0.0	c > 0.0	c > 0.0	c > 0.0	c > 0.0
d	d > 1.000	d > 1.000	d > 1.000	d > 1.000	d > 1.000
Number of points					
Analyzed	30	30	30	30	30

Cumulative Gaussian	
	A
Best-fit values	
Mean	239.9
SD	11.83
Std. Error	
Mean	1.678
SD	3.869
95% Confidence Intervals	
Mean	236.5 to 243.3
SD	3.908 to 19.76
Goodness of Fit	
df	28
R square	0.9998
Absolute SS	9.594
Sy.x	0.5854
Number of points	
Analyzed	30

Reversed Cumulative Gaussian	
	egg
	Ambiguous
Best-fit values	
Mean	~ 13.33
SD	~ 0.3643
Std. Error	
Mean	~
SD	~
95% Confidence Intervals	
Mean	(Very wide)
SD	(Very wide)
Goodness of Fit	
df	28
R square	1
Absolute SS	4.93E-26
Sy.x	4.196E-14
Runs test	
Points above curve	0
Points below curve	1
Number of runs	1
P value (runs test)	
Deviation from Model	Significant
Number of points	
Analyzed	30

P. regina 30.0° C.

***P. regina* 30.0° C**														
Dataset														
Time	egg		L1		L2		L3f		L3m		P		A	
	Mean	SE	Mean	SE	Mean	SE	Mean	SE	Mean	SE	Mean	SE	Mean	SE
0.0	100	0	0	0	0	0	0	0	0	0	0	0	0	0
2.1	100	0	0	0	0	0	0	0	0	0	0	0	0	0
4.0	100	0	0	0	0	0	0	0	0	0	0	0	0	0
6.1	100	0	0	0	0	0	0	0	0	0	0	0	0	0
8.1	100	0	0	0	0	0	0	0	0	0	0	0	0	0
10.1	86.2	8.9	13.8	8.9	0	0	0	0	0	0	0	0	0	0
16.2	0	0	100	0	0	0	0	0	0	0	0	0	0	0
22.1	0	0	100	0	0	0	0	0	0	0	0	0	0	0
29.1	0	0	21.9	14.6	78.1	14.6	0	0	0	0	0	0	0	0
34.1	0	0	4.3	4.8	95.7	4.8	0	0	0	0	0	0	0	0
40.3	0	0	0	0	100	0	0	0	0	0	0	0	0	0
49.1	0	0	0	0	12.9	16.3	87.1	16.3	0	0	0	0	0	0
56.1	0	0	0	0	2.9	5.1	97.1	5.1	0	0	0	0	0	0
64.1	0	0	0	0	0	0	100	0	0	0	0	0	0	0
72.1	0	0	0	0	0	0	70.2	35.7	29.8	35.7	0	0	0	0
80.1	0	0	0	0	0	0	25	43.3	75	43.3	0	0	0	0
94.0	0	0	0	0	0	0	0	0	100	0	0	0	0	0
108.1	0	0	0	0	0	0	1.4	2.4	35.4	28.2	63.2	27.4	0	0
122.0	0	0	0	0	0	0	0	0	1.3	2.3	98.7	2.3	0	0
136.1	0	0	0	0	0	0	0	0	1.3	2.3	98.7	2.3	0	0
149.1	0	0	0	0	0	0	0	0	0	0	100	0	0	0
185.1	0	0	0	0	0	0	0	0	0	0	100	0	0	0
220.1	0	0	0	0	0	0	0	0	0	0	16.9	9.8	83.1	9.8
255.0	0	0	0	0	0	0	0	0	0	0	0	0	100	0
255.0	0	0	0	0	0	0	0	0	0	0	0	0	100	0
255.0	0	0	0	0	0	0	0	0	0	0	0	0	100	0
255.0	0	0	0	0	0	0	0	0	0	0	0	0	100	0
255.0	0	0	0	0	0	0	0	0	0	0	0	0	100	0
255.0	0	0	0	0	0	0	0	0	0	0	0	0	100	0
255.0	0	0	0	0	0	0	0	0	0	0	0	0	100	0
255.0	0	0	0	0	0	0	0	0	0	0	0	0	100	0

Gaussian					
	L1	L2	L3	L3m	P
Best-fit values					
Amplitude	121.6	112.3	111.8	107.5	121.8
Mean	19.43	36.2	60.49	90.41	156.4
SD	4.711	6.763	11.1	11.47	34.9
Std. Error					
Amplitude	2.503	3.86	4.78	1.782	6.904
Mean	0.1108	0.2918	0.5481	0.208	2.225
SD	0.1215	0.3106	0.5632	0.1981	2.048
95% Confidence Intervals					
Amplitude	116.5 to 126.8	104.4 to 120.2	102.0 to 121.5	103.9 to 111.2	107.7 to 135.9
Mean	19.20 to 19.66	35.61 to 36.80	59.37 to 61.61	89.98 to 90.83	151.8 to 160.9
SD	4.462 to 4.959	6.126 to 7.399	9.944 to 12.25	11.06 to 11.87	30.71 to 39.10
Goodness of Fit					
df	28	28	28	28	28
R square	0.992	0.976	0.962	0.9945	0.9378
Absolute SS	151.1	545.4	1061	87.53	2264
Sy.x	2.323	4.414	6.155	1.768	8.993
Constraints					
SD	SD > 0.0	SD > 0.0	SD > 0.0	SD > 0.0	SD > 0.0
Number of points					
Analyzed	31	31	31	31	31

Modified Gaussian					
	L1	L2	L3	L3m	P
	Ambiguous	Ambiguous			
Best-fit values					
a	100	97.89	98.42	100.5	99.57
b	~ 19.66	~ 37.45	60.67	90.79	161.2
c	~ 8.977	~ 9.421	14.13	13.25	53.46
d	~ 21.97	~ 6.594	4.09	2.732	12.99
Std. Error					
a	0.828	11.62	2.712	0.9207	0.1182
b	~ 76011	~ 84.11	0.3202	0.09176	0.2136
c	~ 673399	~ 36.42	0.4659	0.1669	0.191
d	~ 2.911e+007	~ 344.2	0.4018	0.08943	0.9493
95% Confidence Intervals					
a	98.30 to 101.7	74.05 to 121.7	92.86 to 104.0	98.64 to 102.4	99.33 to 99.81
b	(Very wide)	(Very wide)	60.01 to 61.33	90.60 to 90.98	160.7 to 161.6
c	(Very wide)	(Very wide)	13.18 to 15.09	12.90 to 13.59	53.07 to 53.85
d	(Very wide)	(Very wide)	3.266 to 4.915	2.549 to 2.916	11.04 to 14.93
Goodness of Fit					
df	27	27	27	27	27
R square	0.999	0.9992	0.987	0.9988	1
Absolute SS	18.49	17.5	363.5	18.74	1.123
Sy.x	0.8275	0.8052	3.669	0.8332	0.204
Constraints					
c	c > 0.0	c > 0.0	c > 0.0	c > 0.0	c > 0.0
d	d > 1.000	d > 1.000	d > 1.000	d > 1.000	d > 1.000
Number of points					
Analyzed	31	31	31	31	31

Cumulative Gaussian	
	A
	Ambiguous
Best-fit values	
Mean	~ 215.8
SD	~ 4.456
Std. Error	
Mean	~ 0.01449
SD	~ 0.01512
95% Confidence Intervals	
Mean	(Very wide)
SD	(Very wide)
Goodness of Fit	
df	29
R square	1
Absolute SS	9.098E-20
Sy.x	5.601E-11
Number of points	
Analyzed	31

Reversed Cumulative Gaussian	
	egg
	Interrupted
Best-fit values	
Mean	10.48
SD	0.3454
Std. Error	
Mean	
SD	
95% Confidence Intervals	
Mean	
SD	
Goodness of Fit	
df	
R square	
Absolute SS	
Sy.x	
Runs test	
Points above curve	
Points below curve	
Number of runs	
P value (runs test)	
Deviation from Model	
Number of points	
Analyzed	31

P. regina 32.5° C.

P. regina 32.5° C														
Dataset														
Time	egg		L1		L2		L3f		L3m		P		A	
	Mean	SE	Mean	SE	Mean	SE	Mean	SE	Mean	SE	Mean	SE	Mean	SE
0.0	100	0	0	0	0	0	0	0	0	0	0	0	0	0
2.1	100	0	0	0	0	0	0	0	0	0	0	0	0	0
4.1	100	0	0	0	0	0	0	0	0	0	0	0	0	0
6.1	100	0	0	0	0	0	0	0	0	0	0	0	0	0
8.1	100	0	0	0	0	0	0	0	0	0	0	0	0	0
10.1	33.7	35.7	66.3	35.7	0	0	0	0	0	0	0	0	0	0
15.1	0	0	100	0	0	0	0	0	0	0	0	0	0	0
20.1	0	0	100	0	0	0	0	0	0	0	0	0	0	0
25.2	0	0	43.7	24.5	56.3	24.5	0	0	0	0	0	0	0	0
30.3	0	0	0	0	100	0	0	0	0	0	0	0	0	0
35.3	0	0	0	0	100	0	0	0	0	0	0	0	0	0
42.2	0	0	0	0	34.9	37.6	65.1	37.6	0	0	0	0	0	0
49.1	0	0	0	0	0	0	100	0	0	0	0	0	0	0
56.1	0	0	0	0	0	0	100	0	0	0	0	0	0	0
63.1	0	0	0	0	0	0	100	0	0	0	0	0	0	0
70.0	0	0	0	0	0	0	92.6	6	7.4	6	0	0	0	0
82.1	0	0	0	0	0	0	24.5	39.2	75.5	39.2	0	0	0	0
94.1	0	0	0	0	0	0	0	0	100	0	0	0	0	0
106.1	0	0	0	0	0	0	0	0	39.9	28.1	60.1	28.1	0	0
118.0	0	0	0	0	0	0	0	0	0	0	100	0	0	0
130.1	0	0	0	0	0	0	0	0	0	0	100	0	0	0
161.1	0	0	0	0	0	0	0	0	0	0	100	0	0	0
192.1	0	0	0	0	0	0	0	0	0	0	94.8	6.3	5.2	6.3
223.0	0	0	0	0	0	0	0	0	0	0	3	3	97	3
254.0	0	0	0	0	0	0	0	0	0	0	0	0	100	0
254.0	0	0	0	0	0	0	0	0	0	0	0	0	100	0
254.0	0	0	0	0	0	0	0	0	0	0	0	0	100	0
254.0	0	0	0	0	0	0	0	0	0	0	0	0	100	0
254.0	0	0	0	0	0	0	0	0	0	0	0	0	100	0
254.0	0	0	0	0	0	0	0	0	0	0	0	0	100	0
254.0	0	0	0	0	0	0	0	0	0	0	0	0	100	0

Gaussian	L1	L2	L3	L3m	P
Best-fit values					
Amplitude	115.6	110.5	114.2	106.9	126
Mean	17.51	32.79	59.11	91.16	154
SD	5.174	5.996	13.19	10.23	34.84
Std. Error					
Amplitude	6.046	2.272	4.761	1.59	9.053
Mean	0.3043	0.1579	0.7035	0.1764	2.534
SD	0.2741	0.1499	0.6963	0.1738	2.436
95% Confidence Intervals					
Amplitude	103.3 to 128.0	105.9 to 115.2	104.5 to 124.0	103.7 to 110.2	107.4 to 144.5
Mean	16.89 to 18.13	32.47 to 33.11	57.67 to 60.55	90.80 to 91.52	148.9 to 159.2
SD	4.613 to 5.736	5.689 to 6.303	11.76 to 14.61	9.872 to 10.58	29.85 to 39.83
Goodness of Fit					
df	28	28	28	28	28
R square	0.9438	0.9906	0.9607	0.9954	0.9058
Absolute SS	1304	203.4	1411	71.88	3375
Sy.x	6.825	2.695	7.099	1.602	10.98
Constraints					
SD	SD > 0.0	SD > 0.0	SD > 0.0	SD > 0.0	SD > 0.0
Number of points					
Analyzed	31	31	31	31	31

Modified Gaussian	L1	L2	L3	L3m	P
	Interrupted	Interrupted	Ambiguous		Ambiguous
Best-fit values					
a	100	100	98.15	100.8	98.7
b	17.51	33.51	~ 61.70	91.53	~ 163.6
c	7.486	8.228	~ 19.65	11.58	~ 57.48
d	18.4	13.7	~ 27.26	2.691	~ 58.49
Std. Error					
a			0.7121	0.228	0.5004
b			~ 201.2	0.02418	~ 1171
c			~ 138.5	0.0345	~ 1161
d			~ 12315	0.02116	~ 70222
95% Confidence Intervals					
a			96.69 to 99.61	100.4 to 101.3	97.67 to 99.73
b			(Very wide)	91.48 to 91.58	(Very wide)
c			(Very wide)	11.51 to 11.65	(Very wide)
d			(Very wide)	2.647 to 2.734	(Very wide)
Goodness of Fit					
df			27	27	27
R square			0.9989	0.9999	0.9994
Absolute SS			41.07	1.219	20.28
Sy.x			1.233	0.2125	0.8667
Constraints					
c	c > 0.0	c > 0.0	c > 0.0	c > 0.0	c > 0.0
d	d > 1.000	d > 1.000	d > 1.000	d > 1.000	d > 1.000
Number of points					
Analyzed	31	31	31	31	31

Cumulative Gaussian	A
Best-fit values	
Mean	206.4
SD	8.81
Std. Error	
Mean	0.00000193
SD	0.000001133
95% Confidence Intervals	
Mean	206.4 to 206.4
SD	8.810 to 8.810
Goodness of Fit	
df	29
R square	1
Absolute SS	1.935E-10
Sy.x	0.000002583
Number of points	
Analyzed	31

Reversed Cumulative Gaussian	egg
	Ambiguous
Best-fit values	
Mean	~ 9.984
SD	~ 0.2760
Std. Error	
Mean	
SD	
95% Confidence Intervals	
Mean	
SD	
Goodness of Fit	
df	29
R square	1
Absolute SS	0
Sy.x	
Runs test	
Points above curve	
Points below curve	
Number of runs	
P value (runs test)	
Deviation from Model	
Number of points	
Analyzed	31

Supplementary Materials S6

Datasets and statistical analysis summary of *Phormia regina* nonlinear regressions for 50% stage transition by temperature. Regression equation:

y = 100/(1+10^((LogEC50-x)*HillSlope))

where x = time (log time) and y = percent in stage. Where “interrupted” is indicated on a regression, data were insufficient to fit a curve (typically this means we obtained too few points in measuring portions of the transition curve). Where “ambiguous” is indicated on a regression, the program could not reach a final solution by iterative (numerical) methods.. The analysis is from GraphPad Prism 6.0 which uses the Marquardt and Levenberg approach for non-linear regression.

P. regina 10° C.

***P. regina* 10.0° C**	L1	L2	L3f	L3m	P	A
log(agonist) vs. normalized response -- Variable slope						
Best-fit values						
LogEC50	2.239					
HillSlope	3.016					
EC50	173.3					
Std. Error						
LogEC50	0.03211					
HillSlope	0.6061					
95% Confidence Intervals						
LogEC50	2.167 to 2.310					
HillSlope	1.666 to 4.367					
EC50	147.0 to 204.4					
Goodness of Fit						
Degrees of Freedom	10					
R square	0.9431					
Absolute Sum of Squares	1056					
Sy.x	10.28					
Runs test						
Points above curve	6					
Points below curve	6					
Number of runs	4					
P value (runs test)	0.0671					
Deviation from Model	Not Significant					
Number of points						
Analyzed	12					
	LogEC50		HillSlope		EC50	
	Value	SE	Value	SE	Value	SE
L1	2.23884	0.0321147	3.016366	0.6061186	173.3166	

**Dataset**						
* starred and italicized data points were excluded from final regression						
log(time)	L1	L2	L3f	L3m	P	A
1.242106	0					
1.506449	0					
1.682897	0					
1.806858	0					
1.903633	0					
2.094355	27.2					
2.225643	63.3					
2.326626	65					
2.40848	52.5					
2.465383	83.3					
2.560056	100					
2.629495	100					

P. regina 12.5° C.

***P. regina* 12.5° C**	L1	L2	L3f	L3m	P	A
log(agonist) vs. normalized response -- Variable slope				Interrupted	Ambiguous	Ambiguous
Best-fit values						
LogEC50	1.846	2.549	2.752	2.787	~ 2.926	3.162
HillSlope	4.816	154.6	7.75	225.4	~ 197.2	~ 1668
EC50	70.17	354.3	564.3	612.5	~ 843.8	1451
Std. Error						
LogEC50	0.01104	183.4	0.0292		~ 32135	
HillSlope	0.6154	738186	3.742		~ 1.790e+009	
95% Confidence Intervals						
LogEC50	1.818 to 1.875	-406.2 to 411.3	2.689 to 2.814		(Very wide)	
HillSlope	3.234 to 6.398	-1.645e+006 to	-0.2765 to 15.78		(Very wide)	
EC50	65.73 to 74.91	0.0 to +infinity	488.5 to 651.8		(Very wide)	
Goodness of Fit						
Degrees of Freedom	5	10	14		17	21
R square	0.9917	0.9331	0.6585		0.9943	1
Absolute Sum of Squares	93.58	1109	5924		100	0
Sy.x	4.326	10.53	20.57		2.425	
Runs test						
Points above curve	3	3	2		2	
Points below curve	4	9	14		15	
Number of runs	4	4	4		4	
P value (runs test)	0.5429	0.2	0.35		0.3309	
Deviation from Model	Not Significant	Not Significant	Not Significant		Not Significant	
Number of points						
Analyzed	7	12	16	15	19	23
	LogEC50		HillSlope		EC50	
	Value	SE	Value	SE	Value	SE
L1	1.846138	0.01103809	4.81592	0.6153861	70.16783	
L2	2.549373	183.4421	154.6458	738186.2	354.3018	
L3f	2.751508	0.02919853	7.750142	3.742032	564.298	
L3m	2.787087		225.4046		612.4733	
P	2.926265	32135.05	197.1646	1789906000	843.8491	
A	3.161667		1668.087		1450.999	

**Dataset**						
* starred and italicized data points were excluded from final regression						
log(time)	L1	L2	L3f	L3m	P	A
	0	0	0	0	0	0
	0	0	0	0	0	0
	13.8	0	0	0	0	0
	43.2	0	0	0	0	0
	59.9	0	0	0	0	0
	100	0	0	0	0	0
	100	0	0	0	0	0
		0	0	0	0	0
		33.3	0	0	0	0
		0	0	0	0	0
		100	0	0	0	0
		100	0	0	0	0
			0	0	0	0
			100	0	0	0
			17.5	72.5	10	0
			100		0	0
					16.7	0
					100	0
					100	0
						0
						50
						100
						100

P. regina 15.0° C.

***P. regina* 15.0° C**	L1	L2	L3f	L3m	P	A
log(agonist) vs. normalized response -- Variable slope		Interrupted				
Best-fit values						
LogEC50	1.788	2.188	2.363	2.649	2.671	2.985
HillSlope	5.514	149.3	11.39	11.57	11.83	6.461
EC50	61.37	154.1	230.7	445.9	468.6	965.9
Std. Error						
LogEC50	0.01726		0.00533	0.001803	0.00275	0.01478
HillSlope	1.192		1.404	0.4474	0.7589	1.27
95% Confidence Intervals						
LogEC50	1.747 to 1.829		2.352 to 2.375	2.645 to 2.653	2.665 to 2.677	2.954 to 3.015
HillSlope	2.694 to 8.333		8.356 to 14.42	10.62 to 12.52	10.25 to 13.42	3.841 to 9.082
EC50	55.87 to 67.42		224.7 to 236.9	442.0 to 449.9	462.4 to 474.9	900.4 to 1036
Goodness of Fit						
Degrees of Freedom	7		13	15	19	24
R square	0.9861		0.9834	0.9977	0.9939	0.9134
Absolute Sum of Squares	216.3		274.9	2.042	129.3	2071
Sy.x	5.559		4.599	0.369	2.608	9.289
Runs test						
Points above curve	3		2	1	3	3
Points below curve	6		13	16	18	23
Number of runs	6		4	3	5	4
P value (runs test)	0.881		0.3714	1	0.2842	0.0438
Deviation from Model	Not Significant		Not Significant	Not Significant	Not Significant	Significant
Number of points						
Analyzed	9	11	15	17	21	26
	LogEC50		HillSlope		EC50	
	Value	SE	Value	SE	Value	SE
L1	1.787964	0.01725509	5.513623	1.192334	61.37117	
L2	2.187732		149.3093		154.0751	
L3f	2.363065	0.005330136	11.38831	1.404045	230.7095	
L3m	2.649252	0.001802544	11.56888	0.4473526	445.9152	
P	2.670805	0.002750097	11.83429	0.7589026	468.6034	
A	2.984917	0.01477844	6.461477	1.269606	965.8667	

**Dataset**						
* starred and italicized data points were excluded from final regression						
log(time)	L1	L2	L3f	L3m	P	A
0.908485	0	0	0	0	0	0
1.206938	0	0	0	0	0	0
1.382692	0	0	0	0	0	0
1.506449	0	0	0	0	0	0
1.210185	10.9	0	0	0	0	0
1.793674	51.7	0	0	0	0	0
1.930949	90	0	0	0	0	0
2.018492	86.4	0	0	0	0	0
2.111375	100	0	0	0	0	0
2.179516		5.6	0	0	0	0
2.257878		100	0	0	0	0
2.326626			26.7	0	0	0
2.385606			72.1	0	0	0
2.438048			75	0	0	0
2.48446			100	0	0	0
2.584223				15.6	0	0
2.613947				27.8	22.2	0
2.672217					52.5	0
2.720173					77.1	0
2.763537					*92.9*	0
2.857935					*94.4*	0
2.937267						62.8
3.002166						35.6
3.059185						60.3
3.112437						100
3.112437						100

P. regina 17.5° C.

***P. regina* 17.5° C**	L1	L2	L3f	L3m	P	A	**Dataset**						
log(agonist) vs. normalized response -- Variable slope				Interrupted	Interrupted		* starred and italicized data points were excluded from final regression						
Best-fit values							log(time)	L1	L2	L3f	L3m	P	A
LogEC50	1.602	2.104	2.338	2.443	2.503	2.794	0.708279	0	0	0	0	0	0
HillSlope	5.903	9.675	27.91	170.5	225.5	12.23	1.003963	0	0	0	0	0	0
EC50	40.01	126.9	217.6	277.4	318.6	622.5	1.178737	0	0	0	0	0	0
Std. Error							1.300306	0	0	0	0	0	0
LogEC50	0.01112	0.005853	0.0003634			0.006332	1.400394	0	0	0	0	0	0
HillSlope	0.876	1.108	0.3524			1.833	1.605036	56.2	0	0	0	0	0
95% Confidence Intervals							1.709588	74.2	0	0	0	0	0
LogEC50	1.576 to 1.628	2.091 to 2.116	2.337 to 2.339			2.781 to 2.807	1.846492	100	0	0	0	0	0
HillSlope	3.831 to 7.975	7.260 to 12.09	27.16 to 28.67			8.444 to 16.01	1.930758	100	0	0	0	0	0
EC50	37.66 to 42.51	123.2 to 130.7	217.2 to 218.0			604.0 to 641.5	2.001211		5.8	0	0	0	0
Goodness of Fit							2.084576		45.6	0	0	0	0
Degrees of Freedom	7	12	14			24	2.1529		65.6	0	0	0	0
R square	0.9922	0.9889	0.9999			0.9748	2.212554		100	0	0	0	0
Absolute Sum of Squares	128.5	214	0.9392			684.5	2.265496		100	0	0	0	0
Sy.x	4.284	4.223	0.259			5.341	2.312151			16.3	0	0	0
Runs test							2.383965			95	0	0	0
Points above curve	3	3	1			4	2.445727				73.1	0	0
Points below curve	6	11	15			22	2.499973					15.4	0
Number of runs	4	4	3			6	2.547826					100	0
P value (runs test)	0.3452	0.1484	1			0.1408	2.591167					*100*	0
Deviation from Model	Not Significant	Not Significant	Not Significant			Not Significant	2.684898						8.7
							2.761928						21.4
Number of points							2.827046						84.8
Analyzed	9	14	16	17	20	26	2.884229						71.9
	LogEC50		HillSlope		EC50		2.933993						100
	Value	SE	Value	SE	Value	SE	2.933993						100
L1	1.602157	0.01111563	5.903074	0.8760046	40.00895								
L2	2.103514	0.005852587	9.674705	1.10837	126.9154								
L3f	2.337732	0.000363439	27.91256	0.3524276	217.6368								
L3m	2.443181		170.5046		277.4474								
P	2.503255		225.454		318.6065								
A	2.794117	0.006332428	12.22789	1.833339	622.4676								

P. regina 20.0° C.

***P. regina* 20.0° C**	L1	L2	L3f	L3m	P	A
log(agonist) vs. normalized response – Variable slope						
Best-fit values						
LogEC50	1.385	1.873	2.089	2.306	2.389	2.65
HillSlope	15.93	37.75	11.92	12.49	7.19	7.118
EC50	24.26	74.61	122.7	202.4	244.8	447.2
Std. Error						
LogEC50	0.0003807	0.001212	0.009542	0.001248	0.01971	0.01278
HillSlope	0.07176	15.56	2.813	0.4724	2.094	1.313
95% Confidence Intervals						
LogEC50	1.384 to 1.386	1.870 to 1.875	2.068 to 2.110	2.304 to 2.309	2.347 to 2.430	2.624 to 2.677
HillSlope	15.76 to 16.11	3.092 to 72.41	5.840 to 17.99	11.48 to 13.50	2.791 to 11.59	4.401 to 9.834
EC50	24.20 to 24.31	74.15 to 75.08	117.1 to 128.7	201.2 to 203.7	222.5 to 269.3	420.8 to 475.3
Goodness of Fit						
Degrees of Freedom	6	10	13	15	18	23
R square	1	0.9992	0.9262	0.996	0.8416	0.9454
Absolute Sum of Squares	0.01737	12.83	953.4	16.07	2313	1467
Sy.x	0.0538	1.133	8.564	1.035	11.34	7.986
Runs test						
Points above curve	3	2	1	1	2	3
Points below curve	5	10	14	16	18	22
Number of runs	4	4	3	3	4	4
P value (runs test)	0.4286	0.4545	1	1	0.2842	0.0474
Deviation from Model	Not Significant	Not Significant	Not Significant	Not Significant	Not Significant	Significant
Number of points						
Analyzed	8	12	15	17	20	25
	LogEC50		HillSlope		EC50	
	Value	SE	Value	SE	Value	SE
L1	1.384832	0.000380717	15.93346	0.07176174	24.2567	
L2	1.872798	0.001212337	37.75079	15.55602	74.61017	
L3f	2.088984	0.00954158	11.91675	2.813202	122.7394	
L3m	2.306236	0.001248195	12.49111	0.4723944	202.4119	
P	2.388817	0.01970522	7.190236	2.093775	244.8032	
A	2.650498	0.01277997	7.117673	1.312933	447.1963	

**Dataset**						
* starred and italicized data points were excluded from final regression						
log(time)	L1	L2	L3f	L3m	P	A
0.60206	0	0	0	0	0	0
0.910936	0	0	0	0	0	0
1.082187	0	0	0	0	0	0
1.203894	0	0	0	0	0	0
1.304544	5	0	0	0	0	0
1.492411	98.1	0	0	0	0	0
1.625613	100	0	0	0	0	0
1.72581	100	0	0	0	0	0
1.81177		0	0	0	0	0
1.875471		55.8	0	0	0	0
1.959836		96.4	0	0	0	0
2.030161		100	0	0	0	0
2.090772			68.6	0	0	0
2.143119			75	0	0	0
2.190798			76.2	0	0	0
2.262402				23.7	0	0
2.324668				62.3	0	0
2.332438					66.4	0
2.426511					57.7	0
2.562293					96.4	0
2.638489						69.4
2.703291						63.3
2.759668						66.5
2.811966						100
2.811966						100

P. regina 22.5 C.

***P. regina* 22.5° C**	L1	L2	L3f	L3m	P	A
log(agonist) vs. normalized response -- Variable slope	Interrupted					
Best-fit values						
LogEC50	1.19	1.697	1.962	2.127	2.251	2.552
HillSlope	94.11	31.42	9.846	33.7	16.2	18.5
EC50	15.5	49.81	91.63	134	178.3	356.1
Std. Error						
LogEC50		0.001553	0.006643	0.002154	0.00123	0.0005221
HillSlope		4.133	1.325	2.26	0.6253	0.4255
95% Confidence Intervals						
LogEC50		1.694 to 1.701	1.948 to 1.976	2.123 to 2.132	2.249 to 2.254	2.550 to 2.553
HillSlope		22.07 to 40.77	6.985 to 12.71	28.85 to 38.54	14.89 to 17.50	17.62 to 19.38
EC50		49.41 to 50.22	88.66 to 94.71	132.6 to 135.5	177.2 to 179.3	355.2 to 357.0
Goodness of Fit						
Degrees of Freedom		9	13	14	19	24
R square		0.9999	0.9797	0.9972	0.9988	0.9998
Absolute Sum of Squares		1.813	350.8	23.72	32.71	7.33
Sy.x		0.4489	5.195	1.302	1.312	0.5526
Runs test						
Points above curve		2	2	2	3	3
Points below curve		9	13	14	18	23
Number of runs		4	5	4	6	4
P value (runs test)		0.4909	1	0.35	0.4887	0.0438
Deviation from Model		Not Significant	Not Significant	Not Significant	Not Significant	Significant
Number of points						
Analyzed	7	11	15	16	21	26
	LogEC50		HillSlope		EC50	
	Value	SE	Value	SE	Value	SE
L1	1.190394		94.11095		15.50223	
L2	1.697359	0.001552688	31.41866	4.133398	49.81488	
L3f	1.962052	0.006642899	9.846122	1.324794	91.633	
L3m	2.127175	0.002153687	33.69727	2.259697	134.0216	
P	2.251125	0.001229658	16.19546	0.6253129	178.289	
A	2.551577	0.000522125	18.49973	0.4254735	356.104	

**Dataset**						
* starred and italicized data points were excluded from final regression						
log(time)	L1	L2	L3f	L3m	P	A
0.477121	0	0	0	0	0	0
0.781456	0	0	0	0	0	0
0.958046	0	0	0	0	0	0
1.082486	0	0	0	0	0	0
1.18599	27.8	0	0	0	0	0
1.384712	100	0	0	0	0	0
1.516039	100	0	0	0	0	0
1.624712		0	0	0	0	0
1.708987		69.9	0	0	0	0
1.779717		98.5	0	0	0	0
1.863595		100	0	0	0	0
1.935108			45.3	0	0	0
1.995507			58.9	0	0	0
2.04946			94.9	5.1	0	0
2.097416			91.3	8.8	0	0
2.167797				96.1	0	0
2.227972					31.9	0
2.281355					73.4	0
2.328677					96.9	0
2.371245					98.6	0
2.463893					100	0
2.540329						38.7
2.604226						89.9
2.661734						97.9
2.718502						100
2.718502						100

P. regina 25.0° C.

***P. regina* 25.0° C**	L1	L2	L3f	L3m	P	A
log(agonist) vs. normalized response – Variable slope	Interrupted					Interrupted
Best-fit values						
LogEC50	1.181	1.637	1.853	2.091	2.19	2.452
HillSlope	108.3	27.57	13.01	19.76	32.77	148.7
EC50	15.15	43.33	71.3	123.2	154.8	283.4
Std. Error						
LogEC50		0.0001632	0.004192	0.0005511	0.00004895	
HillSlope		0.83	1.47	0.415	0.1186	
95% Confidence Intervals						
LogEC50		1.636 to 1.637	1.844 to 1.862	2.089 to 2.092	2.190 to 2.190	
HillSlope		25.69 to 29.45	9.837 to 16.19	18.87 to 20.64	32.53 to 33.02	
EC50		43.29 to 43.37	69.83 to 72.80	122.9 to 123.6	154.7 to 154.8	
Goodness of Fit						
Degrees of Freedom		9	13	15	19	
R square		1	0.9906	0.9994	1	
Absolute Sum of Squares		0.5233	187	5.058	0.03495	
Sy.x		0.2411	3.793	0.5807	0.04289	
Runs test						
Points above curve		2	1	2	3	
Points below curve		9	14	15	18	
Number of runs		4	3	4	4	
P value (runs test)		0.4909	1	0.3309	0.0669	
Deviation from Model		Not Significant	Not Significant	Not Significant	Not Significant	
Number of points						
Analyzed	7	11	15	17	21	24
	LogEC50		HillSlope		EC50	
	Value	SE	Value	SE	Value	SE
L1	1.180549		108.2923		15.15475	
L2	1.636794	0.000163249	27.56866	0.8299522	43.33057	
L3f	1.853078	0.004192438	13.01129	1.469586	71.29813	
L3m	2.090673	0.000551138	19.75802	0.4150138	123.2176	
P	2.189708	4.89547E-05	32.77439	0.1186227	154.7777	
A	2.452426		148.7319		283.4172	

**Dataset**						
* starred and italicized data points were excluded from final regression						
log(time)	L1	L2	L3f	L3m	P	A
0.4931093	0	0	0	0	0	0
0.7805573	0	0	0	0	0	0
0.9584444	0	0	0	0	0	0
1.081137	0	0	0	0	0	0
1.179695	44.7	0	0	0	0	0
1.346353	100	0	0	0	0	0
1.401185	100	0	0	0	0	0
1.557858		0	0	0	0	0
1.634561		46.5	0	0	0	0
1.699693		97.9	0	0	0	0
1.778332		100	0	0	0	0
1.845641			51.6	0	0	0
1.904558			76.4	0	0	0
1.954685			95	0	0	0
2.000398			96.3	3.8	0	0
2.072311				29.9	0	0
2.133712				88	1.3	0
2.177897					29.1	0
2.238631					97.5	0
2.281166					100	0
2.376851					100	0
2.454845						69.6
2.521138						100
2.579212						100

P. regina 27.5° C.

***P. regina* 27.5° C**	L1	L2	L3f	L3m	P	A
log(agonist) vs. normalized response -- Variable slope	Ambiguous	Interrupted	Interrupted			
Best-fit values						
LogEC50	~ 1.140	1.53	1.753	1.979	2.087	2.379
HillSlope	~ 222.5	157.4	175.5	7.988	32.74	28.93
EC50	~ 13.81	33.85	56.59	95.32	122.1	239.2
Std. Error						
LogEC50	~ 5.381e+018			0.008796	0.00004783	0.0009988
HillSlope	~ 8.725e+021			1.139	0.07576	2.608
95% Confidence Intervals						
LogEC50	(Very wide)			1.960 to 1.998	2.086 to 2.087	2.377 to 2.381
HillSlope	(Very wide)			5.560 to 10.42	32.58 to 32.90	23.52 to 34.34
EC50	(Very wide)			91.30 to 99.53	122.0 to 122.1	238.1 to 240.4
Goodness of Fit						
Degrees of Freedom	5			15	18	22
R square	1			0.9469	1	0.9997
Absolute Sum of Squares	0			522.5	0.04262	7.456
Sy.x	1.327E-29			5.902	0.04866	0.5822
Runs test						
Points above curve	1			1	3	3
Points below curve	5			16	17	21
Number of runs	2			3	4	4
P value (runs test)	0.3333			1	0.0737	0.0514
Deviation from Model	Not Significant			Not Significant	Not Significant	Not Significant
Number of points						
Analyzed	7	11	13	17	20	24

	LogEC50		HillSlope		EC50	
	Value	SE	Value	SE	Value	SE
L1	1.140282	5.38112E+18	222.4792	8.72482E+21	13.8128	
L2	1.529575		157.4303		33.85126	
L3f	1.752776		175.505		56.59478	
L3m	1.979202	0.008796418	7.988264	1.139475	95.32393	
P	2.086572	4.78281E-05	32.73726	0.07575835	122.0597	
A	2.378826	0.00099881	28.93368	2.608432	239.2357	

**Dataset**						
* starred and italicized data points were excluded from final regression						
log(time)	L1	L2	L3f	L3m	P	A
0.2992167	0	0	0	0	0	0
0.6065605	0	0	0	0	0	0
0.7817554	0	0	0	0	0	0
0.9057959	0	0	0	0	0	0
1.003065	0	0	0	0	0	0
1.20985	100	0	0	0	0	0
1.346272	100	0	0	0	0	0
1.464266		0	0	0	0	0
1.533814		82.3	0	0	0	0
1.603595		100	0	0	0	0
1.690713		100	0	0	0	0
1.76349			98.7	0	0	0
1.827073			100	0	0	0
1.882335				0	0	0
1.929887				44.7	0	0
2.004554				59.1	0	0
2.068588				79.5	20.5	0
2.124069					94.4	0
2.216825					100	0
2.312001					100	0
2.389166						66.7
2.454845						96.9
2.511883						100
2.511883						100

P. regina 30.0° C.

***P. regina* 30.0° C**	L1	L2	L3f	L3m	P	A
log(agonist) vs. normalized response -- Variable slope	Interrupted					Interrupted
Best-fit values						
LogEC50	1.012	1.437	1.669	1.878	2.028	2.338
HillSlope	95.46	19.72	36.71	19.81	40.66	148.9
EC50	10.27	27.34	46.62	75.56	106.7	217.8
Std. Error						
LogEC50		0.004002	0.005726	0.0009341	0.0005932	
HillSlope		2.694	9.265	0.7456	4.012	
95% Confidence Intervals						
LogEC50		1.428 to 1.446	1.656 to 1.681	1.876 to 1.880	2.027 to 2.029	
HillSlope		13.50 to 25.93	16.32 to 57.10	18.21 to 21.40	32.26 to 49.05	
EC50		26.77 to 27.93	45.29 to 48.00	75.21 to 75.91	106.4 to 107.0	
Goodness of Fit						
Degrees of Freedom		8	11	14	19	
R square		0.9993	0.9996	0.9986	0.9999	
Absolute Sum of Squares		11.61	7.982	19.21	2.775	
Sy.x		1.205	0.8519	1.171	0.3821	
Runs test						
Points above curve		2	2	2	3	
Points below curve		8	11	14	18	
Number of runs		4	4	4	4	
P value (runs test)		0.5333	0.4231	0.35	0.0669	
Deviation from Model		Not Significant	Not Significant	Not Significant	Not Significant	
Number of points						
Analyzed	7	10	13	16	21	25
	LogEC50		HillSlope		EC50	
	Value	SE	Value	SE	Value	SE
L1	1.011759		95.46452		10.27447	
L2	1.436822	0.004001522	19.71729	2.694262	27.34145	
L3f	1.66861	0.00572557	36.71099	9.265081	46.62409	
L3m	1.878284	0.000934142	19.80556	0.7455657	75.55853	
P	2.02813	0.000593187	40.65754	4.011531	106.6916	
A	2.337973		148.8649		217.7576	

**Dataset**						
* starred and italicized data points were excluded from final regression						
log(time)	L1	L2	L3f	L3m	P	A
0.319626	0	0	0	0	0	0
0.606113	0	0	0	0	0	0
0.783248	0	0	0	0	0	0
0.907367	0	0	0	0	0	0
1.003425	13.8	0	0	0	0	0
1.209403	100	0	0	0	0	0
1.34431	100	0	0	0	0	0
1.46439		78.1	0	0	0	0
1.533073		95.7	0	0	0	0
1.604991		100	0	0	0	0
1.691155			87.1	0	0	0
1.748898			97.1	0	0	0
1.807027			100	0	0	0
1.85786				29.8	0	0
1.903633				75	0	0
1.973128				100	0	0
2.033893					63.2	0
2.086389					98.7	0
2.133792					98.7	0
2.173514					100	0
2.267406					100	0
2.34262						83.1
2.40654						100
2.40654						100
2.40654						100

P. regina 32.5° C.

***P. regina* 32.5° C**	L1	L2	L3f	L3m	P	A
log(agonist) vs. normalized response -- Variable slope		Interrupted				
Best-fit values						
LogEC50	1.139	1.4	1.619	1.978	2.252	2.543
HillSlope	4.308	110.3	14.19	10.99	6.571	11.48
EC50	13.77	25.11	41.59	95.09	178.6	349.1
Std. Error						
LogEC50	0.009731		0.003305	0.01157	0.008096	0.007449
HillSlope	0.4771		1.079	3.094	0.7149	2.115
95% Confidence Intervals						
LogEC50	1.108 to 1.170		1.611 to 1.627	1.953 to 2.003	2.235 to 2.269	2.528 to 2.558
HillSlope	2.789 to 5.826		11.71 to 16.68	4.350 to 17.62	5.084 to 8.058	7.119 to 15.85
EC50	12.82 to 14.79		40.86 to 42.32	89.81 to 100.7	171.8 to 185.6	337.0 to 361.7
Goodness of Fit						
Degrees of Freedom	3		8	14	21	24
R square	0.9917		0.9996	0.8745	0.9719	0.9137
Absolute Sum of Squares	39.59		6.807	1458	575.4	996.9
Sy.x	3.633		0.9224	10.2	5.235	6.445
Runs test						
Points above curve	2		2	2	3	2
Points below curve	3		8	14	20	24
Number of runs	4		4	4	4	4
P value (runs test)	0.9		0.5333	0.35	0.0559	0.2215
Deviation from Model	Not Significant		Not Significant	Not Significant	Not Significant	Not Significant
Number of points						
Analyzed	5	7	10	16	23	26
	LogEC50		HillSlope		EC50	
	Value	SE	Value	SE	Value	SE
L1	1.139	0.009731171	4.307749	0.4771413	13.77211	
L2	1.399831		110.2617		25.10909	
L3f	1.618942	0.003305214	14.19396	1.079273	41.58552	
L3m	1.978156	0.01156718	10.98604	3.093692	95.09459	
P	2.251843	0.008095888	6.571046	0.7148767	178.5843	
A	2.542994	0.007449316	11.48417	2.114877	349.1358	

**Dataset**						
* starred and italicized data points were excluded from final regression						
log(time)	L1	L2	L3f	L3m	P	A
0.611458	0	0	0	0	0	0
0.910491	5.8	0	0	0	0	0
1.094442	43.3	0	0	0	0	0
1.182795	57.7	0	0	0	0	0
1.285932	81.3	0	0	0	0	0
1.402046		63.7	0	0	0	0
1.494039		100	0	0	0	0
1.592732			*90.0**	0	0	0
1.655178			77.2	0	0	0
1.708633			93.1	0	0	0
1.779356			100	0	0	0
1.840603				0	0	0
1.892813				0	0	0
1.940641				52.9	0	0
1.983043				30.3	0	0
2.049735				100	0	0
2.092165					0	0
2.158928					11.1	0
2.20438					36.2	0
2.245831					63.1	0
2.328677					66	0
2.398099					82.8	0
2.439484					96.7	0
2.495035						41.7
2.542825						30.9
2.61066						100
